# To Develop Biomarkers for Diabetic Nephropathy Based on Genes Related to Fibrosis and Propionate Metabolism and Their Functional Validation

**DOI:** 10.1155/2024/9066326

**Published:** 2024-10-16

**Authors:** Sha Li, Jingshan Chen, Wenjing Zhou, Yonglan Liu, Di Zhang, Qian Yang, Yuerong Feng, Chunli Cha, Li Li, Guoyong He, Jun Li

**Affiliations:** ^1^Department of Nephrology, The First Affiliated Hospital of Kunming Medical University 650032, Kunming, China; ^2^Department of Nephrology, The Second People's Hospital of Yunnan Province 650021, Kunming, China; ^3^Department of Nephrology, Kunming First People's Hospital 650034, Kunming, China

## Abstract

Propionate metabolism is important in the development of diabetes, and fibrosis plays an important role in diabetic nephropathy (DN). However, there are no studies on biomarkers related to fibrosis and propionate metabolism in DN. Hence, the current research is aimed at evaluating biomarkers associated with fibrosis and propionate metabolism and to explore their effect on DN progression. The GSE96804 (DN : control = 41 : 20) and GSE104948 (DN : control = 7 : 18) DN-related datasets and 924 propionate metabolism-related genes (PMRGs) and 656 fibrosis-related genes (FRGs) were acquired from the public database. First, DN differentially expressed genes (DN-DEGs) between the DN and control samples were sifted out via differential expression analysis. The PMRG scores of the DN samples were calculated based on PMRGs. The samples were divided into the high and low PMRG score groups according to the median scores. The PM-DEGs between the two groups were screened out. Second, the intersection of DN-DEGs, PM-DEGs, and FRGs was taken to yield intersected genes. Random forest (RF) and recursive feature elimination (RFE) analyses of the intersected genes were performed to sift out biomarkers. Then, single gene set enrichment analysis was conducted. Finally, immunoinfiltrative analysis was performed, and the transcription factor (TF)–microRNA (miRNA)–mRNA regulatory network and the drug–gene interaction network were constructed. There were 2633 DN-DEGs between the DN and control samples and 515 PM-DEGs between the high and low PMRG score groups. In total, 10 intersected genes were gained after taking the intersection of DN-DEGs, PM-DEGs, and FRGs. Seven biomarkers, namely, SLC37A4, ACOX2, GPD1, angiotensin-converting enzyme 2 (ACE2), SLC9A3, AGT, and PLG, were acquired via RF and RFE analyses, and they were found to be involved in various mechanisms such as glomerulus development, fatty acid metabolism, and peroxisome. The seven biomarkers were positively correlated with neutrophils. Moreover, 8 TFs, 60 miRNAs, and 7 mRNAs formed the TF–miRNA–mRNA regulatory network, including USF1-hsa-mir-1296-5p-AGT and HIF1A-hsa-mir-449a-5p-ACE2. The drug–gene network contained UROKINASE–PLG, ATENOLOL–AGT, and other interaction relationship pairs. Via bioinformatic analyses, the risk of fibrosis and propionate metabolism-related biomarkers in DN were explored, thereby providing novel ideas for research related to DN diagnosis and treatment.

## 1. Introduction

Diabetic nephropathy (DN) is a common complications of DM, and a typical cause of end-stage renal disease [1]. To date, it is generally believed that the incidence and development of DN is related to genetics, metabolic disorders, oxidative stress, inflammatory response, and other factors. However, the pathogenesis of DN is still unclear [2]. Currently, at home and other countries, the main treatment methods for DN are lowering blood pressure and blood lipid levels and regulating blood lipid levels. These treatment strategies can delay disease progression. However, there is no specific reversal or eradication therapy for DN [3]. Therefore, its pathogenesis should be further explored, and effective biomarkers must be identified to facilitate early diagnosis and treatment.

Propionate, an organic acid derived from diet, gut microbiome metabolism, and branched-chain amino acids, is deemed safe by the FDA and is widely used in food processing and animal feed [4, 5]. Studies indicate that propionate is crucial for energy metabolism in type 2 diabetes, regulating appetite, glucose, lipid metabolism, and reducing inflammation by activating GPCRs. [6, 7]. Propionate can decrease weight and inhibit insulin resistance [8]. However, Tirosh et al. [9] found that exogenous propionic acid is associated with the rapid activation of the sympathetic nervous system, resulting in an increased expression of glucagon and FABP4. When added to human food, propionic acid may act as a metabolic disruptor in the postprandial state. Other studies have revealed that elevated levels of propionate alone may increase the risk of DN via various mechanisms. Propionate levels ≥ 4.4 *μ*g/mL exhibit a threshold effect, with levels surpassing this threshold correlating with a significantly increased risk of DN. Olfr78, a receptor of SCFAs, plays an important functional role in regulating glomerular filtration rate and renin release. Further, propionic acid may affect blood pressure by binding to the Olfr-78 receptor, thereby causing kidney damage [9, 10]. Despite its importance in diabetes, the specific role of propionate metabolism in DN remains unclear, necessitating further research.

Fibrosis is a process characterized by the excessive deposition of extracellular matrix, resulting in the replacement of functional parenchymal tissues by fibrotic tissues [11]. RRenal fibrosis is a common effect of DN progression and a common final pathway for several disease. The association between propionic acid metabolism and DN is extremely complex. Further studies should be performed to better understand the specific mechanisms of chronic and progressive kidney diseases [12]. Its main manifestations are excessive deposition of extracellular matrix, proliferation of numerous fibroblasts and myofibroblasts, mesenchymal transition of renal tubular epithelial cells, and proliferation of the blood vessel walls [13]. Renal fibrosis is a key pathological process in the development of DN. Therefore, it is essential to evaluate the mechanism of fibrosis in DN for DN treatment.

In this study, DN-related datasets were obtained from the Gene Expression Omnibus (GEO) database. Propionate metabolism-related genes (PMRGs) and fibrosis-related genes (FRGs) were extracted from the GeneCards database. Then, differential expression analysis, random forest (RF), recursive feature elimination (RFE), and other bioinformatic analyses using the abovementioned data were performed to identify associated biomarkers. Finally, single gene set enrichment analysis (GSEA) and immune infiltration analysis of various biomarkers were performed, and the transcription factor (TF)–microRNA (miRNA)–mRNA and drug–gene interaction networks were also constructed. The use of fibrosis and PMRGs as biomarkers of DN, which is significantly important in DN diagnosis and treatment, was explored.

## 2. Materials and Methods

### 2.1. Source of Data

Two transcriptome datasets related to DN were procured from the GEO database (https://www.ncbi.nlm.nih.gov/gds). The GSE96804 (GPL17586) dataset comprised 41 DN specimens and 20 control glomerular tissue samples. Similarly, the GSE104948 (GPL22945) dataset contained 7 DN samples and 18 control samples from glomerular tissues. Conducting a search on the GeneCard database (https://www.genecards.org/) (Relevance score > 5) with the terms “propionate metabolism” and “fibrosis” resulted in the identification of 924 PMRGs and 656 FRGs.

### 2.2. Differential Expression Analysis, Gene Set Variation Analysis (GSVA), and Functional Enrichment Analysis

In the GSE96804 dataset, DN differentially expressed genes (DN-DEGs) between DN and control samples were sifted out by limma package (Version 3.52.2) [14] setting |log2FoldChange| > 0.5 and adj.*p* < 0.05. Next, we used PMRGs as the background gene set and calculated the PMRG scores of DN samples by ssGSEA algorithm. Then, the samples were divided into high/low PMRG score groups (high = 21 and low = 20) by the median value of the scores, and PM-DEGs between these 2 groups were screened out (adj. *p* < 0.05 and |log2FoldChange| > 0.5). Finally, the intersection of DN-DEGs, PM-DEGs, and FRGs was taken to acquire the intersected genes. After that, the Gene Ontology (GO) and Kyoto Encyclopedia of Genes and Genomes (KEGG) enrichment analyses were performed on them by clusterProfiler package (Version 4.4.4) [15]. Moreover, the STRING (https://string-db.org) website was utilized to predict the interactions among intersected genes (Confidence = 0.15), and protein–protein interaction (PPI) network of them was constructed.

### 2.3. RF Analysis and Construction of Artificial Neural Network (ANN) Model

This study conducted RF on the intersected genes and utilized RFE to acquire the importance ranking of each gene, the error rate, and the accuracy of each iteration combination. Then, we used the genes that corresponded to the lowest error rate point as biomarkers. Furthermore, the expression of biomarkers was compared between DN and control samples in the GSE96804 and GSE104948 datasets via wilcox.test. Subsequently, an ANN model focusing on biomarkers was developed utilizing the neuralnet package [16]. The model's validity was assessed using receiver operating characteristic (ROC) analysis conducted on both the training set from GSE96804 and the validation set from GSE104948. Finally, the correlation of expressions among biomarkers was compared by Spearman's analysis.

### 2.4. Functional Annotation of Biomarkers

In order to analyze the functional similarity of the biomarkers, we calculated the semantic similarity among their GO terms through GOSemSim (Version 2.22.0) package [17]. Correlations between biomarkers and other genes were calculated and sorted in the GSE96804 dataset using the default background gene set in org.Hs.eg.db package (Version 3.15.0). Subsequently, each biomarker was subjected to GSEA using the clusterProfiler package (Version 4.4.4) using the criteria |NES| > 1, adj.*p* < 0.05, *q*.value < 0.2 [15].

### 2.5. Construction of the Logistic Regression Model (LRM)

To further determine the ability of the biomarkers to differentiate between samples, based on the GSE96804 dataset, a multivariate LRM and nomogram were constructed using the rms package (Version 6.3-0) [18]. To evaluate the predictive performance of the model, calibration curves were created and the ROC analysis was performed using the pROC package (Version 1.18.0) [19], and the results were validated in the GSE104948 validation set. Finally, a decision curve analysis (DCA) curve was conducted to evaluate the degree of benefit for DN patients (intervention for high-risk patients and avoidance for low-risk patients).

### 2.6. Immunity Infiltration Analysis

First, the proportion of 22 immune cells in all samples (*n* = 61) of the GSE96804 dataset was calculated based on the immune cell LM22 gene set using the CIBERSORT algorithm. Secondly, we clustered the immune cells in DN and normal samples and compared the correlation among immune cells utilizing Spearman analysis. The differences in the expression of immune cells between DN and control samples were analyzed by wilcox.test, and the immune cells with significant differences were extracted. Finally, we analyzed the correlation between immune cells with significant differences and biomarkers.

### 2.7. Construction of TF-miRNA-mRNA Regulatory Network and Prediction of Potential Therapeutic Agents

To further explore the potential regulatory mechanisms of biomarkers for DN, TFs as well as miRNAs for biomarkers were predicted in the miRNet database (https://www.mirnet.ca/), and the TF-miRNA-mRNA network was visualized using Cytoscape (Version 3.8.2). Secondly, the biomarker-associated potential therapeutic agents were explored through the drug-gene interaction database (DGIdb) (https://dgidb.genome.wustl.edu/), and we constructed a drug-gene interaction network.

### 2.8. Statistical Analysis

All data were processed using the R package. In the realm of multiple hypothesis testing, we employed the Benjamini–Hochberg procedure, a method of false discovery rate (FDR) correction, to regulate the ratio of false discoveries among all the results of the significance tests. Component comparisons were conducted using the rank sum test, and statistical significance was determined with *p* < 0.05.

## 3. Results

### 3.1. Identification of DEGs and Intersected Genes

**Figure 1:**
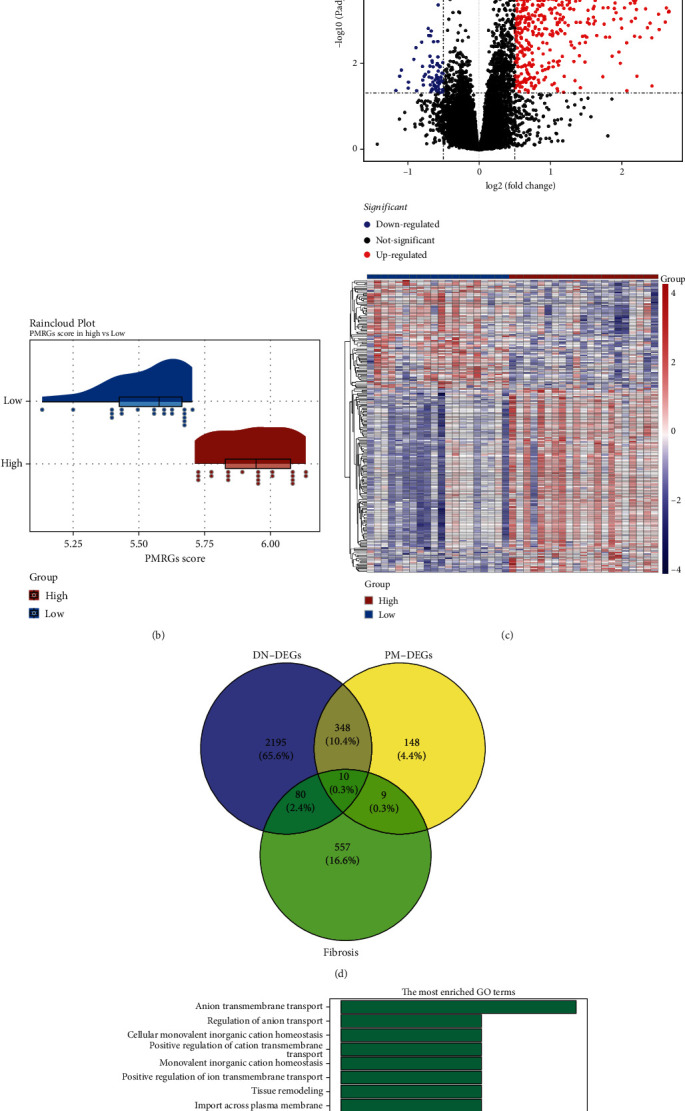
Differential expression analysis and functional enrichment analysis. (a) The volcano map and heat map of differentially expressed genes (DEGs) between diabetic nephropathy (DN) and control samples. (b) The raincloud plot of propionate metabolism-related genes (PMRGs) score. (c) The volcano map and heat map of 515 propionate metabolism-related DEGs (PM-DEGs). (d) The Venn diagram of 10 intersected genes obtained by overlapping DN-DEGs, PM-DEGs, and fibrosis-related genes (FRGs). (e, f) The Gene Ontology (GO) terms and Kyoto Encyclopedia of Genes and Genomes (KEGG) pathways enriched in intersected genes. BP, biological progress; CC, cellular component; MF, molecular function (MF). (g) The protein–protein interaction (PPI) network of intersected genes.

In total, 2633 DN-DEGs between the DN and control samples were obtained (Figure 1(a), Table S1).  Figure 1(b) shows the PMRG scores of the high/and low PMRG score groups. We acquired 515 PM-DEGs between the two groups (Figure 1(c), Table S2). By taking the intersection of DN-DEGs, PM-DEGs, and FRGs, 10 intersected genes were obtained (Figure 1(d)). These genes were enriched in 310 GO entries, including the regulation of inflammatory response, very long-chain fatty acid metabolic process, and purine-containing compound metabolic process (Figure 1(e)). The intersected genes were involved in five KEGG pathways, such as the renin–angiotensin system, proximal tubule bicarbonate reclamation, and protein digestion and absorption (Figure 1(f)). The PPI network comprised 9 nodes and 16 edges, including reciprocal relationship pairs such as SLC9A3–AGT, angiotensin-converting enzyme 2 (ACE2)–NOX4, and SLC4A4–GPD1 (Figure 1(g)).

### 3.2. Acquisition of Biomarkers

**Figure 2:**
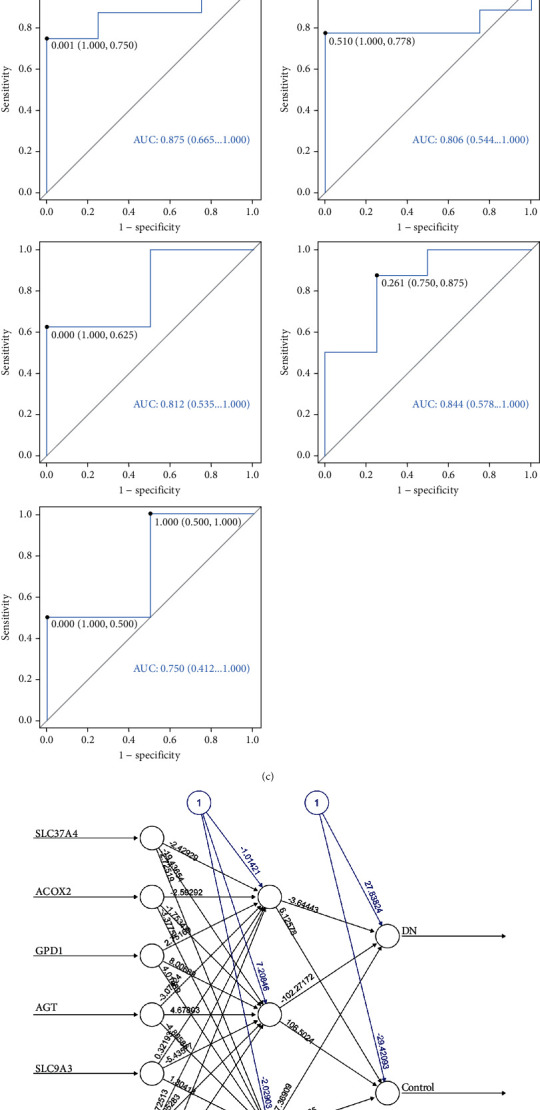
Identification of biomarkers. (a, b) The accuracy and importance ranking of feature gene in random forest (RF) model. (c) The receiver operating characteristic (ROC) curves of 5x cross-validated. AUC, area under the curve. (d) The artificial neural network (ANN) model of biomarkers. (e) The importance of each gene to the model prediction outcome.

After employing RF and RFE (Figures 2(a) and 2(b)), a total of 7 biomarkers—SLC37A4, ACOX2, GPD1, ACE2, SLC9A3, AGT, and PLG—were identified. These biomarkers exhibited lower expression levels in DN samples, with the expression of SLC37A4, GPD1, AGT, and PLG showing significant differences in the two datasets (Figure S1). In the 5-fold cross-validation, the AUC values of the ROC curves for each fold were all greater than 0.7, respectively, indicating that the model has good discriminatory ability (Figure 2(c)), while Figure 2(d) illustrates the completion of the entire training process in 2160 steps. Notably, SLC37A4 emerged as the most critical variable for classification (Figure 2(e)). In addition, the area under the curve (AUC) of the ANN model (AUC > 0.9) surpassed that of each individual biomarker in both the training and validation sets, underscoring its robust accuracy and stability (Figure S2). Furthermore, correlation analysis revealed significant positive correlations among the 7 biomarkers, with GPD1 and PLG displaying the strongest correlation (Figure S3; Figure S4).

### 3.3. Functional Annotation Analysis

**Figure 3:**
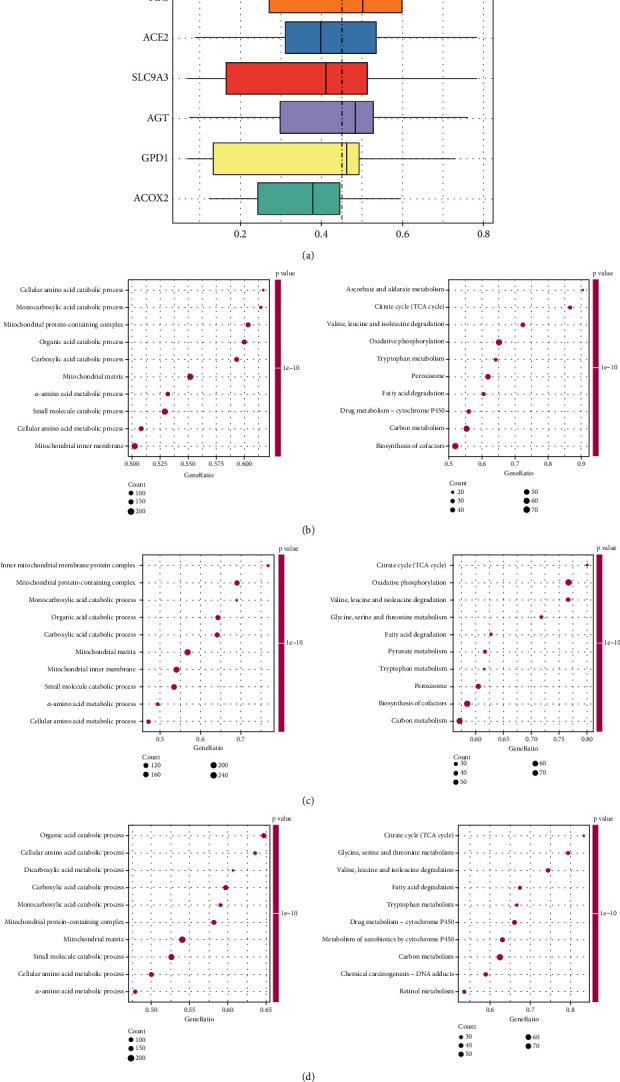
Functional enrichment analysis. (a) The boxplot of the GO semantic similarity of the key genes. (b–d) Gene Set Enrichment Analysis (GSEA) of (b) ACE2, (c) ACOX2, and (d) AGT.

**Figure 4:**
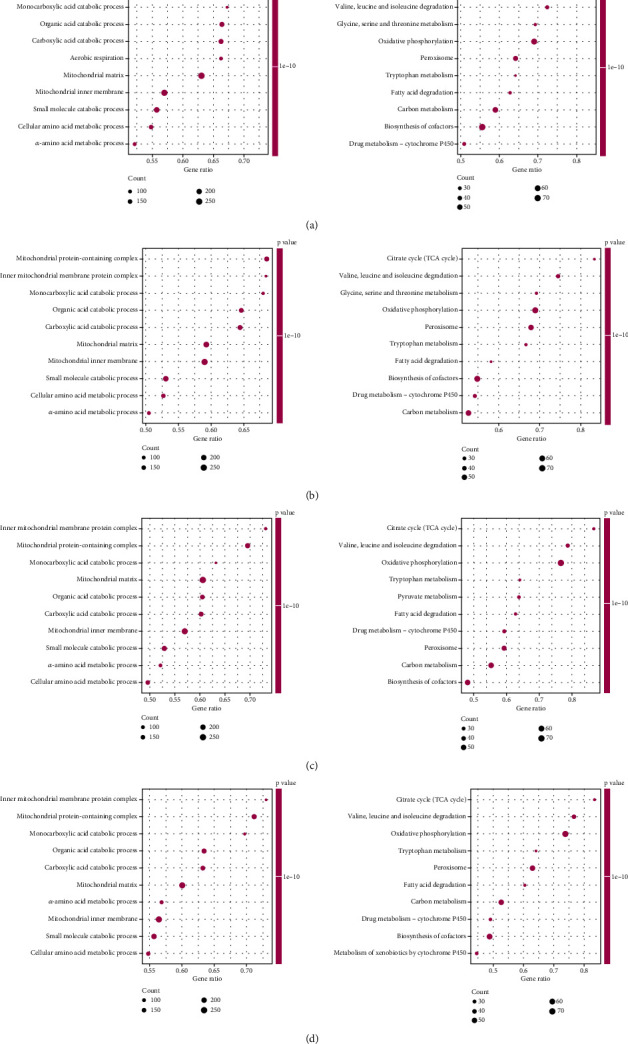
Functional enrichment analysis. (a–d) GSEA analysis of GPD1, PLG, SLC9A3, and SLC37A4.

After GO semantic similarity analysis, PLG, AGT, and GPD1 were found at the core of the interaction (Figure 3(a)). ACE2 was enriched during mechanisms such as sugar transmembrane transporter activity, glomerulus development, and fatty acid metabolism (Figure 3(b)). ACOX2 was involved in mechanisms such as organic acid catabolic process, oxidative phosphorylation, and peroxisome (Figure 3(c)). AGT was associated with small molecule catabolic process, leukocyte-mediated immunity, and biosynthesis of nucleotide sugars and other substances (Figure 3(d)). GPD1 was enriched in mechanisms including mitochondrial matrix, valine, leucine, and isoleucine degradation (Figure 4(a)). PLG was engaged in mechanisms such as fatty acid catabolic process, mitochondrial respirasome, and vascular smooth muscle contraction (Figure 4(b)). SLC9A3 played a role in processes such as cellular respiration, inner mitochondrial membrane protein complex, and arginine and proline metabolism (Figure 4(c)). SLC37A4 was enriched in mechanisms such as complex, microtubule-based movement, and drug metabolism-cytochrome (Figure 4(d)).

### 3.4. Biomarker Assessment

**Figure 5:**
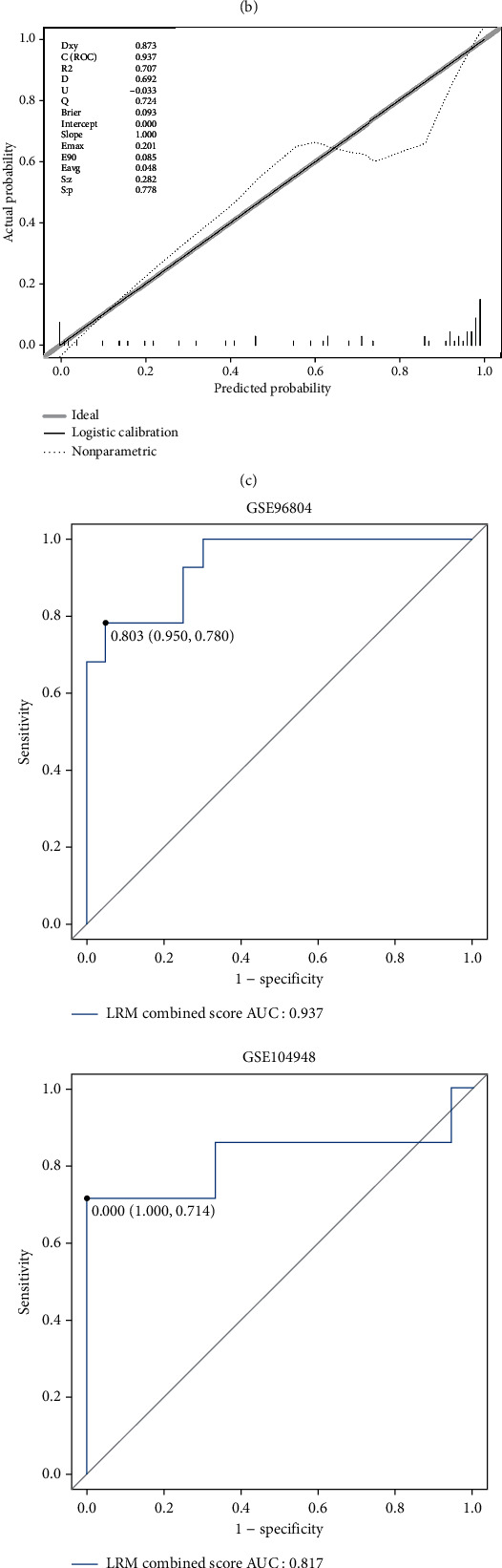
Identification of biomarkers and exploration of their clinical significance. (a) The multivariate logistic regression model of 7 biomarkers. (b) The nomogram was constructed to predict the odd ratio of DN based on the biomarkers. (c) The calibration curve of the nomogram. (d) The ROC curves of logistic regression model in the GSE96804 and GSE104948 datasets. (e) The decision curve analysis (DCA) curve of the nomogram.

The LRM results showed that ACE2 was a risk factor and SLC37A4 was a protective factor influencing the development of DN (Figure 5(a)). Meanwhile, based on the nomogram, the biomarkers were effective in predicting DN. In addition, the correction curve further validated the accuracy of the prediction results (Figures 5(b) and 5(c)). Importantly, the AUC values of the nomogram were > 0.8 in both the training and validation sets. This finding indicated an accurate ability to distinguish DN from normal samples (Figure 5(d)). Moreover, according to the DCA map, the nomogram model curve was higher than the ALL curve. Therefore, the biomarkers could be utilized in patients with DN (Figure 5(e)).

### 3.5. Immune Microenvironment Analysis

**Figure 6:**
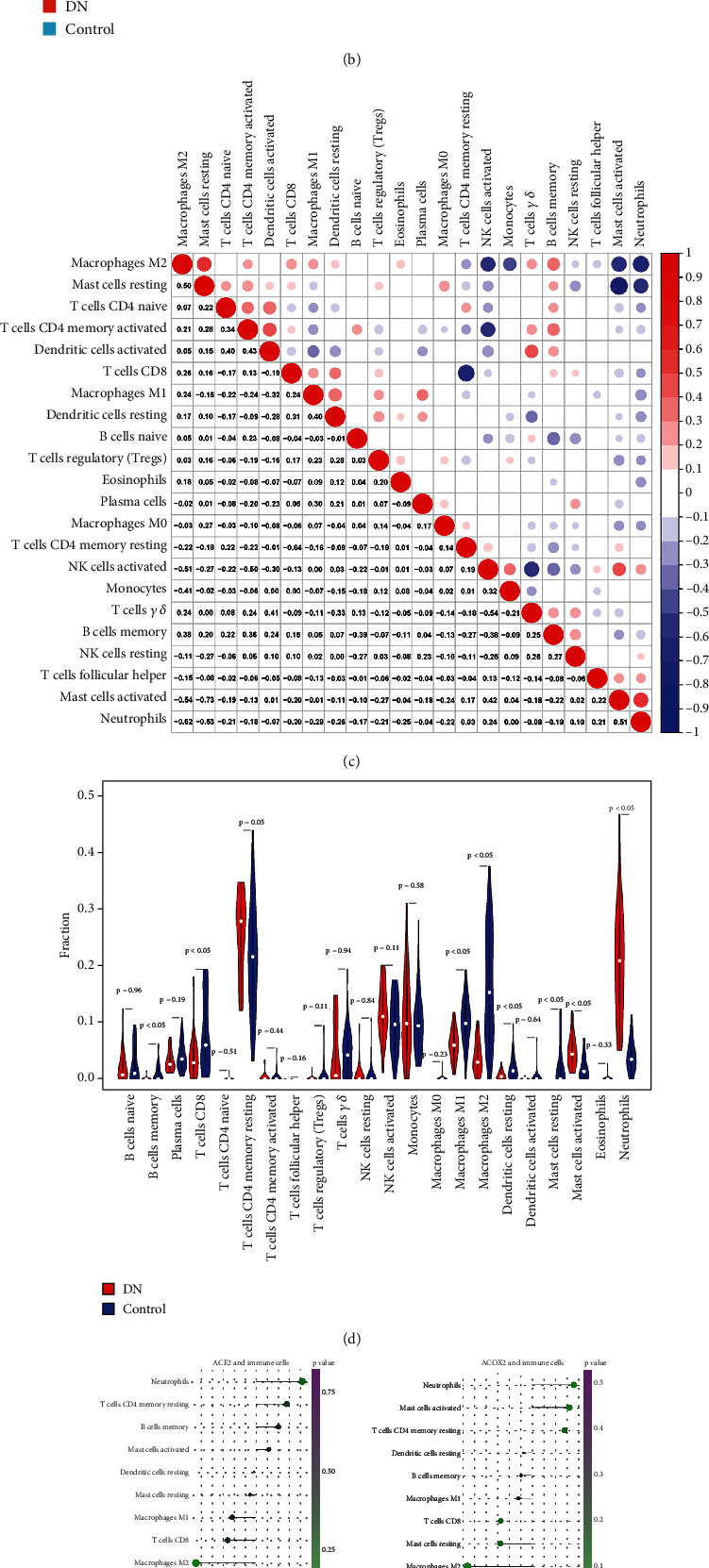
Immune infiltration analysis. (a, b) The bar graph and heat map of the proportion of immune cells in DN and control samples. (c) The relevance of immune cells. (d) Discrepancies of the fraction of immune cells in DN and control samples. (e) The relevance of biomarkers to differential immune cells.

Figures 6(a) and 6(b) showed the proportion and abundance of immune cells in each sample, respectively. The correlations among immune cells differed. For example, activated mast cells were negatively correlated with resting mast cells and were positively associated with activated NK cells (Figure 6(c)). As seen in the violin plot, the fraction of nine immune cells was significantly different between the DN and control samples, such as memory B cells, CD8 T cells, and M2 macrophages (Figure 6(d)). The correlation analysis found that seven biomarkers were positively correlated with neutrophils (Figure 6(e)).

### 3.6. TF-miRNA-mRNA Regulatory Network and Potential Therapeutic Agents

**Figure 7:**
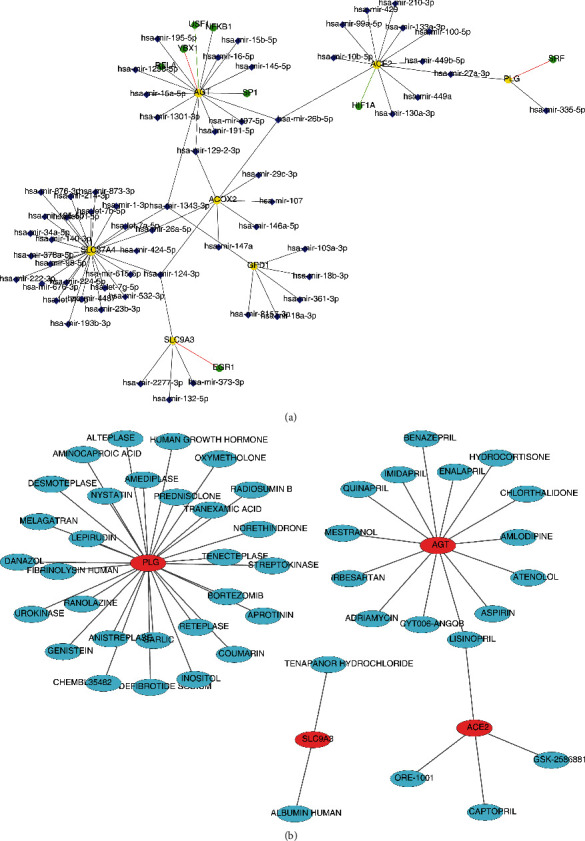
Construction of the regulatory network and drugs prediction. (a) The network established based on biomarkers, microRNAs (miRNAs), and transcription factors (TFs). Blue diamonds represent miRNAs; yellow circles represent biomarkers; green circles represent TFs. (b) The drug-biomarker interaction network. Red represents biomarkers and biue represents drugs.

The TF-miRNA-mRNA regulatory network comprised 8 TFs, 60 miRNAs, and 7 mRNAs, with interactions such as USF1-hsa-mir-1296-5p-AGT, HIF1A-hsa-mir-449a-5p-ACE2, and EGR1-hsa-mir-2277-3p-SLC9A3 (Figure 7(a)). Further, ACE2, AGT, PLG, and SLC9A3 separately corresponded to 4, 14, 29, and 2 drugs. The drug-gene network included UROKINASE-PLG, ATENOLOL-AGT, CAPTOPRIL-ACE2, and other interaction relationship pairs (Figure 7(b)).

## 4. Discussion

DM is a metabolic disease mainly characterized by chronic hyperglycemia. If the patient's blood glucose level is not well controlled for a long time, various complications can develop. DN is one of the most important microvascular complications of DM and is the main cause of end-stage renal disease [20]. Its pathogenesis is still unclear, and there is a lack of effective treatment options. The short-chain fatty acid propionate participates in a series of metabolic processes such as the regulation of appetite, glucose levels, and lipid metabolism and the reduction of inflammatory response by activating G protein–coupled receptors and deacetyl–histone receptors, which may play a key role in the incidence and development of diabetes and DN [6, 21]. Fibrosis plays a role in DN progression and is essential for the progression of type 1 and type 2 DN to renal failure [22]. Patients with DN eventually develop renal fibrosis. This study screened out seven biomarkers (SLC37A4, ACOX2, GPD1, ACE2, SLC9A3, AGT, and PLG) related to propionate metabolism and fibrosis in DN via bioinformatic analyses.

SLC37A4, also known as glucose-6-phosphate translocase (G6PT), functions with glucose-6-phosphatase-alpha or glucose-6-phosphatase-beta to hydrolyze glucose-6-phosphate to phosphate, and glucose and release it from the endoplasmic reticulum, thereby maintaining glucose homeostasis [23]. Mutations in SLC37A4 could cause glycogen storage disease type 1b (GSD1b), which is characterized by abnormal glycogen storage in the liver and kidney with neutropenia and neutrophil dysfunction. Kidney disease is a common complication of GSD1b. Patients with GSD1b present with significant glycogen accumulation in the kidney, which leads to renal failure [24]. Angiotensin-converting enzyme 2 (ACE2) is a regulatory enzyme of the renin–angiotensin system, and its main biological effect is to hydrolyse angiotensin II for producing angiotensin 1-7. Renal local renin–angiotensin system–activation plays an essential role in DN progression, and the dysregulation of ACE2 expression is closely related to the incidence and development of kidney disease [25]. The remaining biomarkers, such as ACOX2 [26], AGT [27], and PLG [28], are significantly related to the development of DN. ACOX2, which is related to the oxidation of peroxisome fatty acids, may affect the lipid metabolism of DN [26]. AGT is part of the renin-angiotensin system, which affects blood pressure and kidney function. Epiberberine has been shown to improve DN by AGT, thereby inhibiting the TGF*β*/Smad2 pathway [29]. PLG is involved in fibrinolysis and affects extracellular matrix remodeling. Chen et al. found that PLG was related to kidney and urinary system abnormalities through aqueous humor proteomics analysis, providing a new reference for early detection and diagnosis of DN [30].

Recent studies have found that immune and inflammatory responses play an important role in the incidence and development of DN [31]. Nine types of immune cells were found to differ between DN and control by immune infiltration analysis. Among them, macrophages, mast cells, T cells, and other inflammatory cells are involved in the process of chronic inflammation and interstitial fibrosis in several kidney diseases. Macrophages are the main immune cells that promote diabetic kidney injury [32]. Macrophages are mainly divided into two phenotypes: M1 type (classical type, with proinflammatory effects) and M2 type (alternating activation type, with anti-inflammatory effects). The cells that cause diabetic kidney injury are mainly M1-type macrophages, which can damage podocytes via MCP-1. In contrast, the transfection of M2 macrophages in diabetic mice resulted in a reduction of renal infiltration of macrophages and alleviated renal pathologies, such as tubular atrophy and renal interstitial fibrosis [33]. Macrophages play an important role in the incidence and development of DN, which is the cause of renal interstitial hyperplasia and glomerular injury. M1 is the main site of renal injury in diabetes mellitus. Inflammasome activation can cause chronic renal inflammation and renal fibrosis via the M1 phenotype. M2 promotes the clearance of fibrous tissues via phagocytosis. The transfer of M2 to streptozotocin–induced type 1 diabetes and reduced renal infiltration by macrophages, thereby alleviating tubular atrophy and glomerulosclerosis. The inhibition of M1 macrophage activation and the promotion of M2 macrophage transformation can prevent podocyte injury [9]. Based on data analysis, the proportion of macrophages significantly differed between DN and control samples. The abovementioned studies supported our results and showed that the seven genes might be effective biomarkers for predicting diabetic DN.

The miRNA forms a regulatory network involving TF–miRNA–mRNA, with ACE2 expression regulated by miR-429. miR-429 is crucial for cell invasion, migration, proliferation, and apoptosis. It is significantly downregulated in renal clear cell carcinoma tissues and acts as a tumor suppressor by inhibiting epithelial–mesenchymal transition in cancer cells [34]. Wang et al. found that miR-429 could have protective effects against angiotensin II–induced renal injury in renal nontumor cells [35]. Zhang et al. revealed that the reduction of ACE2 expression induced by endosulfan was regulated by miR-429. Further, they found that ACE2 is a direct target gene of miR-429 [36]. However, there are limited reports on how miR-429 regulates ACE2 to produce biological effects. The specific mechanism is still unclear, and more in-depth research on this notion should be performed. Previous studies have shown that the miR-192 expression decreases in the early stage of DN, and the miR-192 expression decreases with a lower urine albumin-creatinine ratio, which is valuable for the early diagnosis of DN and can also be used as a potential therapeutic target for DN [37]. Mao et al. found that miR-192 is an important medium for transforming growth factor beta 1 (TGF-*β*1) signaling in vitro renal fibrosis. In the process of renal fibrosis, miR-192 is regulated by TGF-*β*1 via Smad3. Astragaloside IV can reduce the expression of miR-192 and inhibit renal mesangial hyperproliferation and renal fibrosis via the TGF-*β*1/Smad/miR-192 signaling pathway [38]. Thus, it has a therapeutic effect on DN. The miR-181c levels of elderly patients with frailty who present with diabetes mellitus and heart failure with preserved ejection fraction were significantly high, and miR-181c targeted PRKN and SMAD7 in human cardiac fibroblasts [39].

Angiotensin–converting enzyme inhibitors (ACEi), such as captopril and liopelil, and mineralocorticoid receptor antagonists (MRA), like finerenone, are well-established in the treatment of DN and are included in current guidelines due to their efficacy in managing hypertension and cardiovascular diseases. Captopril has vasodilator effects and is widely used in the treatment of hypertension and cardiovascular diseases [40]. Further, it is used to treat hypertensive diabetes with nephropathy [41]. However, there is a lack of relevant research on the specific role and therapeutic effect of DN. Liopelil is also an angiotensin-converting enzyme inhibitor. It can improve the permeability of the glomerular basement membrane and improve renal hemodynamics, thereby reducing the excretion of urinary protein and delaying glomerulosclerosis [42]. Liopelil is often used in combination with other drugs, and it has a good effect against DN [43]. Furthermore, MRAs, such as finerenone, is a highly selective third-generation nonsteroidal receptor that significantly reduces the risk of cardiovascular and renal complications and improves cardiovascular-renal outcomes in patients with type 2 diabetes mellitus who develop chronic kidney disease and/or chronic heart failure. Fenenone has a protective effect on the kidney by binding to the mineralocorticoid receptor (MR) and inhibiting the recruitment of transcriptional cofactors related to the expression of hypertrophic, proinflammatory, and profibrotic genes [44]. Despite the established roles of these drugs, our study is aimed at delving deeper into their specific molecular mechanisms and therapeutic effects in the context of DN. Ranolazine is a piperazine derivative that has antianginal and antiarrhythmic properties, and it is used to treat stable angina pectoris [45]. Ranolazine alleviates contrast-associated acute kidney injury, and it may have antidiabetic effects [46]. Sacubitrile–valsartan can be a potent regulator of diabetes-associated metabolic abnormalities and is superior to valsartan alone, which can normalize insulin and glycosylated hemoglobin [47]. Other drugs, such as irbesartan [48], bortezomib [49], and chlorthalidone [50], have also been used in the treatment of DN, which provides a new basis for DN management.

The current study had several possible limitations. That is, as our data come from public databases, there might have been inconsistent or missing data, and limited sample selection could have caused selection bias. Further, there were consistencies between different data sources, and data might be updated over time, thereby affecting stability. Moreover, more in-depth studies on the specific role of each marker in DN and the related pathogenesis should be explored. However, the biomarkers screened out in this study were strongly correlated with DN, and the regulatory network and drugs of these biomarkers were predicted, which can provide novel methods and strategies for understanding the incidence, development, diagnosis, and treatment of DN.

## 5. Conclusions

SLC37A4, ACOX2, GPD1, ACE2, SLC9A3, AGT, and PLG are associated with propionate metabolism and fibrosis based on the bioinformatics analysis, which had excellent power to differentiate control from DN samples. According to the immune infiltration analysis, differential immune cells, particularly neutrophils, were associated with the incidence and development of DN. In addition, since functional enrichment analysis was performed and the TF–miRNA–mRNA regulatory network was established, this research can provide novel theoretical support for DN diagnosis and treatment.

## Data Availability

GSE96804 and GSE104948 datasets analyzed in this study were acquired via the GEO (https://www.ncbi.nlm.nih.gov/gds) database.

## Supporting Information

Supporting Information 1 Table S1: 2633 differentially expressed genes (DEGs) between diabetic nephropathy (DN) and control samples.

Supporting Information 2 Table S2: 515 DEGs between high- and low-propionate metabolism-related genes (PMRGs) score groups.

Supporting Information 3 Figure S1: the expression of biomarkers in DN and control samples. a: GSE96804; b: GSE104948.

Supporting Information 4 Figure S2: the ROC curves and confusion matrix of biomarkers in GSE96804 and GSE104948 datasets.

Supporting Information 5 Figure S3: the correlation of 7 biomarkers.

Supporting Information 6 Figure S4: scatter plot of correlation analysis for biomarkers.
