# Supplementary material for: To Develop Biomarkers for Diabetic Nephropathy Based on Genes Related to Fibrosis and Propionate Metabolism and Their Functional Validation
Source: J Diabetes Res. 2024 Oct 16;2024:9066326. doi: 10.1155/2024/9066326 (PMC11498995; doi:10.1155/2024/9066326)
Supplement: Supporting Information 1 — Table S1: 2633 differentially expressed genes (DEGs) between diabetic nephropathy (DN) and control samples. [file 9066326.f1.pdf]

	logFC	AveExpr	t	P.Value	adj.P.Val	B
G6PC	-4.695155	8.9169818	-10.6768	1.22E-15	1.21E-12	25.285189
FOS	-3.70053	6.2280277	-15.80346	2.41E-23	4.82E-19	42.390975
HBA1	-3.405963	12.784646	-8.60974	3.67E-12	5.00E-10	17.470167
ZFP36	-3.29342	10.245086	-11.00076	3.60E-16	4.46E-13	26.469567
S100A9	-3.292743	9.8326816	-9.072817	5.92E-13	1.17E-10	19.253123
PDK4	-3.274594	9.8632616	-12.29618	3.14E-18	8.82E-15	31.072496
GSTA2	-3.176792	11.812219	-6.147323	6.40E-08	1.33E-06	7.9424813
SNORD3B-1	-3.106989	9.3330007	-12.67673	8.11E-19	3.20E-15	32.382271
SNORD3B-2	-3.106989	9.3330007	-12.67673	8.11E-19	3.20E-15	32.382271
SNORD3A	-3.041903	9.9064921	-12.2201	4.12E-18	1.06E-14	30.808311
SNORD3D	-3.036063	8.7096692	-12.88491	3.90E-19	3.01E-15	33.090415
HPD	-2.972531	9.3861472	-6.383084	2.54E-08	6.28E-07	8.8408717
SNORD3C	-2.971298	8.9332428	-12.59977	1.06E-18	3.66E-15	32.118986
EGR1	-2.933958	9.8467602	-6.618755	1.00E-08	2.88E-07	9.7451757
S100A12	-2.910527	5.6313407	-8.732271	2.26E-12	3.46E-10	17.943264
HIST2H2AA4	-2.83175	11.280837	-12.35143	2.58E-18	7.96E-15	31.263873
ALB	-2.822326	7.5170941	-6.434685	2.08E-08	5.28E-07	9.0383889
MIOX	-2.661908	9.87977	-5.389351	1.18E-06	1.50E-05	5.1143666
DEFA1B	-2.61595	7.7582226	-2.781487	0.0071713	0.0216183	-3.140417
GSTA1	-2.603988	13.268744	-5.356932	1.33E-06	1.66E-05	4.9960076
AK4P3	-2.538019	9.4773741	-6.924262	3.00E-09	1.04E-07	10.924544
PTGS2	-2.516348	5.4211513	-8.148578	2.29E-11	2.08E-09	15.683027
HBB	-2.505058	10.871868	-8.140848	2.36E-11	2.11E-09	15.653
ESM1	-2.459413	7.7732687	-6.852509	3.98E-09	1.33E-07	10.646959
AGMAT	-2.454816	9.6584874	-4.9917	5.21E-06	5.18E-05	3.6812028
OTTHUMG000	-2.420414	13.287924	-6.427727	2.13E-08	5.39E-07	9.0117364
BRE-AS1	-2.396392	5.9815861	-6.066345	8.78E-08	1.74E-06	7.635606
LOC727944	-2.387604	6.3544764	-8.087658	2.92E-11	2.46E-09	15.446343
GLYAT	-2.377295	11.081026	-4.958301	5.89E-06	5.76E-05	3.5628176
OTTHUMG000	-2.362254	12.409681	-6.190687	5.40E-08	1.16E-06	8.1071896
HAO2	-2.355668	8.594	-4.795136	1.07E-05	9.48E-05	2.9893787
SLC12A3	-2.303335	7.0467754	-5.046996	4.25E-06	4.36E-05	3.8779235
HBA2	-2.227431	12.912414	-7.332882	5.90E-10	2.73E-08	12.510179
PAH	-2.170361	11.187202	-4.718888	1.41E-05	0.0001197	2.7243354
JUNB	-2.168754	7.2733244	-8.339042	1.07E-11	1.12E-09	16.422219
RGS2	-2.114402	10.135506	-6.362027	2.76E-08	6.73E-07	8.7603593
KNG1	-2.090167	10.622124	-4.156911	0.0001014	0.0006228	0.836248
GLYATL1	-2.047067	8.6346172	-5.034076	4.46E-06	4.54E-05	3.83188
LOC1005059	-2.009512	6.9606751	-4.58116	2.31E-05	0.0001798	2.2506072
HSPA1B	-2.007365	9.5432103	-11.40624	7.99E-17	1.47E-13	27.933726
PCK1	-2.007097	7.7641749	-6.031264	1.01E-07	1.95E-06	7.5029625
DCXR	-1.995166	11.907191	-8.326129	1.13E-11	1.16E-09	16.372147
ERRFI1	-1.984001	10.58073	-8.98285	8.43E-13	1.52E-10	18.907855
AFM	-1.981443	8.1322052	-4.639499	1.87E-05	0.0001515	2.4504576
ALDOB	-1.98097	16.504808	-4.553856	2.54E-05	0.0001953	2.1574892
DUSP1	-1.953111	7.4002241	-15.57141	5.01E-23	5.16E-19	41.693819
CXCR1	-1.943937	4.7105543	-10.26425	5.84E-15	3.68E-12	23.759076
TMEM174	-1.935141	9.3563318	-3.861503	0.000273	0.0014148	-0.103288
CYP4A11	-1.92619	9.0452115	-3.7171	0.0004371	0.0021003	-0.547389
CXCR2	-1.915092	5.1434177	-11.81619	1.78E-17	3.92E-14	29.392616

HSPA1A	-1.902224	9.2534331	-11.1679	1.93E-16	2.71E-13	27.075605
HSD17B14	-1.899706	6.2751784	-11.70321	2.68E-17	5.53E-14	28.992753
SNORD66	-1.894234	3.6695202	-8.808176	1.68E-12	2.77E-10	18.235886
FBP1	-1.893625	7.8269492	-6.34113	3.00E-08	7.20E-07	8.6805069
FPR1	-1.890064	5.9054957	-7.466569	3.46E-10	1.81E-08	13.03011
TMEM150C	-1.884532	12.546728	-7.38098	4.87E-10	2.34E-08	12.697198
SNORA48	-1.878831	12.586624	-6.234598	4.55E-08	1.02E-06	8.2742303
CTXN3	-1.868615	7.080982	-5.76549	2.81E-07	4.52E-06	6.5045029
UMOD	-1.85502	9.938788	-3.287347	0.0016735	0.0064883	-1.803525
SNORA22	-1.830999	8.8046659	-7.200008	1.00E-09	4.29E-08	11.993847
ACMSD	-1.814049	7.4126275	-6.576299	1.19E-08	3.30E-07	9.581868
S100A8	-1.808454	5.98735	-9.65261	6.15E-14	1.94E-11	21.462591
LOC1009962	-1.808353	6.9360421	-5.680109	3.90E-07	5.94E-06	6.1864041
FAM151A	-1.803453	8.5145608	-4.585176	2.27E-05	0.0001779	2.2643257
PRODH2	-1.803171	7.8160462	-6.058165	9.06E-08	1.78E-06	7.604662
OTTHUMG000	-1.802878	8.4421431	-3.549462	0.0007459	0.0032875	-1.049478
RNF152	-1.801097	9.110989	-6.167913	5.91E-08	1.25E-06	8.0206537
RBP5	-1.801043	12.228173	-5.154252	2.85E-06	3.12E-05	4.2619636
ALDH6A1	-1.800501	10.39118	-5.984044	1.21E-07	2.26E-06	7.3247174
SMCO3	-1.786173	8.5946097	-7.844159	7.69E-11	5.43E-09	14.499455
LOX	-1.784044	9.9650485	-6.571153	1.21E-08	3.36E-07	9.5620817
FAM180A	-1.782193	7.5776846	-6.775659	5.40E-09	1.71E-07	10.350029
BHMT	-1.780104	11.637237	-4.113731	0.0001175	0.0007049	0.6964451
CYP2B6	-1.75373	5.9797166	-6.908848	3.19E-09	1.10E-07	10.864886
SLC36A2	-1.752105	8.0311105	-4.682263	1.61E-05	0.0001335	2.5977166
ACSM2B	-1.751911	12.023591	-4.003971	0.0001701	0.0009589	0.3448124
SLC6A19	-1.730039	7.9846646	-4.671974	1.67E-05	0.0001378	2.5622286
OTTHUMG000	-1.728506	10.161142	-5.595896	5.39E-07	7.73E-06	5.8740549
APOM	-1.69312	9.6921605	-4.310438	5.98E-05	0.0003996	1.3397684
FABP1	-1.692341	8.8711336	-5.817196	2.30E-07	3.85E-06	6.6977995
SLC13A3	-1.687896	9.9030772	-4.082228	0.0001307	0.0007708	0.5949682
SAT2	-1.685281	10.07326	-9.219891	3.32E-13	7.67E-11	19.816246
CDH10	-1.676834	4.5484408	-8.053191	3.35E-11	2.71E-09	15.312388
EHD3	-1.650008	11.305393	-4.945056	6.18E-06	5.98E-05	3.5159618
UGT2B7	-1.645449	12.719362	-3.772757	0.000365	0.0018075	-0.377456
GIPC2	-1.644845	8.0523257	-5.843366	2.08E-07	3.53E-06	6.7958187
XPNPEP2	-1.640841	8.7836556	-4.127366	0.0001121	0.0006786	0.7405046
NAT8B	-1.640059	11.27011	-4.396966	4.42E-05	0.00031	1.6278127
HRSP12	-1.638561	10.366173	-5.766466	2.80E-07	4.51E-06	6.5081459
SLC5A12	-1.63782	11.229523	-3.261076	0.0018111	0.006923	-1.876907
HIST1H1E	-1.633116	13.055692	-8.796859	1.75E-12	2.86E-10	18.19228
TNNI1	-1.629297	10.550459	-5.329294	1.48E-06	1.81E-05	4.8953036
ACSM2A	-1.622479	10.637281	-4.001425	0.0001716	0.0009649	0.3367231
PSAT1	-1.615037	8.8500949	-4.060753	0.0001405	0.000819	0.5260434
NPHS1	-1.614433	11.59298	-5.212945	2.29E-06	2.59E-05	4.4734466
DEFB1	-1.612799	16.315448	-4.176495	9.48E-05	0.0005882	0.8999234
TM4SF5	-1.601253	8.2908107	-5.093292	3.58E-06	3.77E-05	4.0432986
HPGD	-1.578559	8.5761115	-4.330357	5.58E-05	0.0003774	1.4058112
ANGPTL3	-1.571603	7.1628372	-4.41137	4.20E-05	0.0002976	1.6760525
NAT8	-1.561951	10.156718	-3.701474	0.0004597	0.0021914	-0.594813
SLC7A7	-1.559774	8.4643528	-6.56375	1.25E-08	3.44E-07	9.5336283

A1CF	-1.559283	5.444242	-7.46227	3.52E-10	1.83E-08	13.013384
CA2	-1.556193	8.9634333	-8.325795	1.13E-11	1.16E-09	16.370853
CR1	-1.544675	11.595366	-4.578718	2.33E-05	0.0001811	2.2422653
ECHS1	-1.543631	10.461413	-7.28901	7.03E-10	3.18E-08	12.339641
HIST1H2BD	-1.540291	6.8400436	-10.0417	1.37E-14	7.05E-12	22.927961
TPPP3	-1.539668	9.1117793	-6.871339	3.70E-09	1.25E-07	10.719775
C19orf77	-1.539185	12.768806	-3.287589	0.0016722	0.0064844	-1.802848
HES1	-1.535557	9.4530566	-7.578626	2.22E-10	1.25E-08	13.466124
MT1H	-1.531394	16.901038	-4.183049	9.27E-05	0.0005773	0.9212686
FCGR3B	-1.52008	6.8173887	-8.086412	2.93E-11	2.47E-09	15.441502
AK4	-1.519461	9.2285138	-6.199221	5.23E-08	1.13E-06	8.1396348
GATM	-1.515349	13.261307	-3.653457	0.0005363	0.0024881	-0.739752
GBA3	-1.512539	7.1018907	-4.87856	7.89E-06	7.36E-05	3.2815333
IGF1	-1.512387	9.343772	-2.86845	0.0056425	0.0177616	-2.922466
EGF	-1.506972	5.5716616	-5.836258	2.14E-07	3.61E-06	6.7691828
CPXM1	-1.496122	7.2162434	-4.732418	1.34E-05	0.0001149	2.7712267
G0S2	-1.495626	8.5225556	-6.794081	5.02E-09	1.61E-07	10.421171
CYP4A22	-1.486978	6.2084349	-3.975299	0.0001872	0.0010368	0.2538635
LOC1010602	-1.485004	5.0938738	-4.332276	5.54E-05	0.0003754	1.4121821
AKR7A3	-1.474808	5.8198874	-8.394808	8.61E-12	9.44E-10	16.638384
RNU5E-1	-1.473994	5.6729002	-5.650666	4.37E-07	6.51E-06	6.0770357
ALDH4A1	-1.472722	7.4045675	-6.436309	2.06E-08	5.26E-07	9.0446081
AGXT2	-1.471932	9.2053072	-3.478303	0.000932	0.003957	-1.258031
CUBN	-1.467734	11.61853	-2.918544	0.0049047	0.0157889	-2.794601
BTG2	-1.465656	10.457588	-9.17346	3.99E-13	8.74E-11	19.638649
WDR72	-1.464223	8.7165126	-4.981993	5.40E-06	5.34E-05	3.646763
OTTHUMG000	-1.463749	8.1190395	-5.249282	2.00E-06	2.32E-05	4.6048293
OTTHUMG000	-1.457743	8.8170382	-3.020853	0.0036674	0.0123723	-2.528309
PEPD	-1.454318	10.807702	-6.776059	5.39E-09	1.71E-07	10.351577
TMEM52B	-1.451766	9.1071892	-3.36271	0.0013313	0.0053704	-1.590755
PI3	-1.451504	4.2099897	-5.693696	3.71E-07	5.68E-06	6.2369294
ACY1	-1.450322	7.8061521	-5.769417	2.77E-07	4.46E-06	6.519167
APOH	-1.447109	5.4646433	-4.937492	6.36E-06	6.12E-05	3.4892255
SLC22A8	-1.44175	9.5913126	-3.478432	0.0009317	0.0039561	-1.257655
OTTHUMG000	-1.434305	8.7423892	-5.420071	1.05E-06	1.36E-05	5.2267511
SLC7A8	-1.43181	12.560321	-5.856905	1.98E-07	3.38E-06	6.8465732
DDC	-1.429084	7.9155511	-4.706027	1.47E-05	0.0001243	2.6798204
LINC00948	-1.423469	6.242299	-5.364052	1.30E-06	1.62E-05	5.0219807
CALB1	-1.416135	7.1179146	-3.805262	0.0003283	0.0016544	-0.277491
VNN2	-1.411099	6.3547672	-8.578896	4.15E-12	5.46E-10	17.350946
HGD	-1.409828	8.1554769	-4.552486	2.55E-05	0.0001961	2.1528238
RNU5B-1	-1.408864	6.4085536	-7.927232	5.53E-11	4.10E-09	14.822624
SLC4A4	-1.404505	9.6818207	-3.38602	0.0012396	0.0050473	-1.524272
DANCR	-1.395021	6.2716331	-10.86158	6.07E-16	6.95E-13	25.962287
FABP3	-1.392771	9.1707898	-5.378593	1.23E-06	1.54E-05	5.0750616
SNORD41	-1.37648	4.4612831	-4.832948	9.32E-06	8.50E-05	3.1215223
CHI3L1	-1.370942	12.329987	-3.777121	0.0003599	0.0017863	-0.364067
DPP6	-1.367635	8.8045279	-5.766733	2.80E-07	4.50E-06	6.5091448
ECH1	-1.366288	10.94386	-10.27047	5.70E-15	3.68E-12	23.782249
SNORD14E	-1.363942	6.1274567	-4.296925	6.27E-05	0.000415	1.2950556
SNORA12	-1.357311	5.6715733	-5.7904	2.56E-07	4.17E-06	6.5975658

LRRC2	-1.34565	9.8691916	-5.213021	2.29E-06	2.59E-05	4.4737221
OTTHUMG000	-1.345342	4.546301	-5.670711	4.05E-07	6.11E-06	6.1514738
WDR49	-1.345301	7.0840866	-4.52725	2.79E-05	0.0002114	2.067013
FRY-AS1	-1.34434	6.8956059	-5.977726	1.24E-07	2.30E-06	7.3008927
SLC7A9	-1.341402	7.6544697	-3.803434	0.0003303	0.001663	-0.283126
GDF15	-1.34114	4.4431644	-7.17938	1.09E-09	4.62E-08	11.913738
DAO	-1.336592	7.0937561	-4.232296	7.83E-05	0.0005003	1.0822511
PBLD	-1.334282	8.3682893	-5.402144	1.13E-06	1.44E-05	5.1611421
VASN	-1.328964	11.001102	-6.781572	5.28E-09	1.68E-07	10.372862
MORN2	-1.327117	8.0133511	-10.75212	9.16E-16	1.01E-12	25.561689
CLEC4E	-1.326353	4.3843741	-5.401085	1.13E-06	1.44E-05	5.1572663
BHMT2	-1.324668	11.340649	-4.155715	0.0001018	0.000625	0.8323671
SORD	-1.319227	6.9082277	-5.685716	3.82E-07	5.83E-06	6.207251
ACY3	-1.317691	6.8915495	-6.031592	1.00E-07	1.95E-06	7.5042036
KIAA1191	-1.316534	11.776192	-9.706292	5.00E-14	1.64E-11	21.665653
FMO1	-1.311719	9.2432293	-3.199793	0.0021748	0.0080646	-2.046473
LPL	-1.306678	6.4543797	-6.929628	2.93E-09	1.02E-07	10.945316
SLC22A6	-1.306202	9.4701157	-3.15047	0.0025161	0.0090823	-2.181283
PPBP	-1.302604	6.2153454	-4.632813	1.92E-05	0.0001544	2.427493
ABAT	-1.295987	7.326231	-5.349222	1.37E-06	1.70E-05	4.9678979
ALPL	-1.293606	5.9131313	-7.072246	1.66E-09	6.45E-08	11.497964
CYB5A	-1.289733	13.89748	-7.741597	1.16E-10	7.60E-09	14.100362
CRYAA	-1.289583	7.4757833	-6.002927	1.12E-07	2.12E-06	7.3959558
RNU5E-2P	-1.288177	6.4411808	-5.793142	2.53E-07	4.14E-06	6.6078171
PDZK1IP1	-1.286397	11.398177	-6.810307	4.71E-09	1.53E-07	10.483852
FMN2	-1.286383	7.8876897	-5.522069	7.14E-07	9.84E-06	5.6014353
TMEM27	-1.285262	10.25047	-2.989474	0.004012	0.0133511	-2.610709
PTH1R	-1.283563	11.949147	-5.542555	6.60E-07	9.22E-06	5.6769683
PVALB	-1.283182	6.293033	-5.019151	4.71E-06	4.77E-05	3.7787525
ASPA	-1.280502	8.0376816	-6.025842	1.03E-07	1.98E-06	7.4824798
FOLH1B	-1.275443	6.0257372	-4.9791	5.46E-06	5.39E-05	3.6365041
OTTHUMG000	-1.27377	7.172731	-5.98343	1.21E-07	2.26E-06	7.3224006
DACH1	-1.26796	13.72561	-5.15093	2.89E-06	3.16E-05	4.2500226
RNU4-2	-1.265094	6.1433752	-5.524582	7.07E-07	9.76E-06	5.6106964
KCNJ15	-1.262366	8.9226213	-3.984389	0.0001816	0.0010106	0.282656
CRYL1	-1.260787	7.6783333	-7.022414	2.03E-09	7.57E-08	11.304745
C14orf164	-1.254162	5.4837505	-6.248565	4.31E-08	9.69E-07	8.3274158
ATP6V1G3	-1.250879	6.2026156	-2.862184	0.0057417	0.0180069	-2.938342
BLVRB	-1.250568	7.883901	-10.39172	3.59E-15	2.58E-12	24.232697
PLG	-1.249419	7.1513448	-4.110416	0.0001188	0.0007113	0.6857481
HIST2H2BE	-1.246782	8.6346934	-7.304923	6.59E-10	3.01E-08	12.401491
JUN	-1.244521	6.2681005	-9.585149	8.00E-14	2.45E-11	21.207023
TCF21	-1.241581	14.54342	-4.766882	1.18E-05	0.0001031	2.8909428
ANKS4B	-1.240446	4.2028393	-6.384814	2.53E-08	6.24E-07	8.8474893
OTTHUMG000	-1.238621	4.8257943	-4.684099	1.60E-05	0.0001329	2.6040549
PCOLCE2	-1.237517	12.118921	-5.502719	7.69E-07	1.04E-05	5.5301767
NR4A2	-1.236753	4.9522916	-4.637776	1.88E-05	0.0001523	2.4445406
PCBD1	-1.233249	10.167015	-9.99558	1.63E-14	7.67E-12	22.75507
METTL7B	-1.23267	7.5033597	-3.703458	0.0004568	0.0021798	-0.588799
PLGLA	-1.232179	7.5361941	-4.300591	6.19E-05	0.0004105	1.307178
ARHGAP19	-1.230451	10.336946	-5.365125	1.29E-06	1.61E-05	5.0258949

CTSL2	-1.230197	7.2171085	-5.128365	3.14E-06	3.39E-05	4.1689817
CKB	-1.228935	6.4467949	-10.0684	1.24E-14	6.73E-12	23.02795
NFIL3	-1.223895	5.7197379	-9.069764	5.99E-13	1.17E-10	19.241419
OTTHUMG00(	-1.222933	4.3667195	-4.002658	0.0001708	0.0009616	0.3406409
TYRO3	-1.222902	11.15287	-4.415538	4.14E-05	0.0002946	1.6900238
MRO	-1.217692	5.3110777	-4.998868	5.07E-06	5.06E-05	3.7066535
ASS1	-1.217517	12.758284	-5.635955	4.62E-07	6.82E-06	6.0224564
SMIM2-AS1	-1.212225	7.0041561	-6.309183	3.40E-08	7.94E-07	8.558527
MT1G	-1.211619	17.702993	-4.259646	7.13E-05	0.0004629	1.1720946
SNORD14D	-1.211071	4.8038641	-4.84449	8.93E-06	8.21E-05	3.1619494
MAOA	-1.209216	7.800312	-8.074191	3.08E-11	2.56E-09	15.394009
ACADSB	-1.205493	10.320456	-4.653367	1.78E-05	0.0001455	2.4981432
DPEP1	-1.203546	6.5516859	-5.462322	8.96E-07	1.19E-05	5.3816749
SNORD59B	-1.203434	4.4608243	-3.893637	0.0002456	0.0012938	-0.00306
LOC728290	-1.19887	5.1597313	-4.998913	5.07E-06	5.06E-05	3.7068127
PLCG2	-1.197624	8.021991	-2.569707	0.0126137	0.0345255	-3.649157
SLC17A1	-1.197164	9.1424931	-3.082958	0.0030653	0.0107053	-2.36336
OTTHUMG00(	-1.196541	6.1459543	-5.322166	1.52E-06	1.85E-05	4.8693641
MNDA	-1.193135	7.6562631	-4.288475	6.45E-05	0.0004254	1.267133
CERS6	-1.192575	12.441078	-5.71001	3.48E-07	5.41E-06	6.2976443
SOST	-1.18755	11.9045	-4.06689	0.0001377	0.0008059	0.5457196
EHHADH	-1.185571	7.2000495	-5.099883	3.49E-06	3.70E-05	4.0668913
PTGDS	-1.181105	10.728707	-7.117737	1.39E-09	5.60E-08	11.674449
KDR	-1.179335	12.821397	-4.374501	4.78E-05	0.0003315	1.5527439
PRKAR2B	-1.178188	9.451662	-5.830513	2.19E-07	3.67E-06	6.7476617
AQP9	-1.176709	4.9688974	-7.123923	1.36E-09	5.49E-08	11.698458
OTTHUMG00(	-1.174775	8.6722508	-5.385832	1.20E-06	1.51E-05	5.1015088
OTTHUMG00(	-1.174761	4.6983552	-4.229993	7.89E-05	0.0005034	1.0746978
USP2	-1.166768	6.7580892	-4.556124	2.52E-05	0.0001941	2.1652128
LOC100505(	-1.165615	6.4414049	-5.391187	1.17E-06	1.49E-05	5.1210787
PAK1	-1.165063	11.353975	-5.942465	1.42E-07	2.56E-06	7.1680546
SLC34A1	-1.16214	9.1845302	-2.730364	0.0082394	0.0242582	-3.26612
GSTA3	-1.161603	4.9294872	-8.329596	1.12E-11	1.15E-09	16.385591
FOXQ1	-1.161337	6.1263679	-3.4946	0.0008859	0.0038003	-1.210516
NPL	-1.154849	7.1573857	-5.965139	1.30E-07	2.39E-06	7.2534519
C11orf54	-1.153079	12.10252	-5.011142	4.85E-06	4.88E-05	3.7502684
SST	-1.15022	7.0339854	-3.352248	0.0013745	0.0055127	-1.62049
CMBL	-1.147771	5.6784718	-6.227791	4.67E-08	1.04E-06	8.2483213
PIPOX	-1.145933	7.2136877	-4.666151	1.70E-05	0.0001401	2.5421595
ALDH2	-1.144663	9.3180525	-7.703171	1.35E-10	8.44E-09	13.95082
ETFB	-1.144655	9.5316115	-8.460779	6.63E-12	7.82E-10	16.893931
SLC13A1	-1.136537	9.1158608	-2.662722	0.0098766	0.0282548	-3.429631
NEBL-AS1	-1.135714	4.9031605	-10.4256	3.15E-15	2.32E-12	24.358255
FXYD1	-1.13329	7.3683638	-6.823915	4.46E-09	1.46E-07	10.536431
SNORD13	-1.132358	13.113652	-3.142746	0.0025739	0.0092486	-2.20226
SBSPON	-1.132026	12.47201	-4.486582	3.23E-05	0.0002393	1.929229
CX3CL1	-1.128854	12.102577	-7.012483	2.11E-09	7.79E-08	11.266251
APLN	-1.128363	8.6906038	-5.310675	1.59E-06	1.92E-05	4.8275695
TREM1	-1.126336	4.2063377	-7.131723	1.31E-09	5.35E-08	11.728728
SLC2A2	-1.126282	6.6989905	-4.011618	0.0001658	0.0009384	0.3691358
ACAA2	-1.123512	11.439093	-4.773942	1.15E-05	0.000101	2.9155126

EGOT	-1.122623	6.0874223	-5.00498	4.96E-06	4.97E-05	3.7283652
PDZK1	-1.122597	12.382822	-3.744227	0.0004004	0.0019503	-0.46476
SLC47A1	-1.122517	8.2272807	-3.469016	0.0009593	0.0040603	-1.285041
RASD1	-1.122505	3.7275649	-5.385401	1.20E-06	1.52E-05	5.0999307
MT1F	-1.121932	16.983144	-6.151379	6.30E-08	1.32E-06	7.9578746
HTRA1	-1.114518	14.742452	-5.436006	9.90E-07	1.29E-05	5.285132
ALKBH7	-1.112168	9.4976246	-11.28447	1.25E-16	2.04E-13	27.496204
RRAS	-1.110729	9.8500638	-6.282031	3.78E-08	8.65E-07	8.454952
MIR614	-1.107501	12.084719	-3.397175	0.0011979	0.0049046	-1.492348
LINC00052	-1.106792	5.5344874	-4.349808	5.21E-05	0.0003558	1.4704571
RNU5D-2P	-1.103371	6.4070587	-5.431123	1.01E-06	1.31E-05	5.2672377
MPP5	-1.099911	13.518366	-4.830529	9.40E-06	8.57E-05	3.113055
PDZK1P1	-1.098865	11.590253	-3.683094	0.0004877	0.0023042	-0.650433
DPYS	-1.098202	6.3509148	-4.536535	2.70E-05	0.0002053	2.0985587
PLTP	-1.098049	10.455367	-5.770599	2.76E-07	4.46E-06	6.5235792
EPHX2	-1.096798	7.4837782	-5.973643	1.26E-07	2.33E-06	7.2855013
L3MBTL3	-1.09589	8.1645959	-5.453184	9.28E-07	1.22E-05	5.3481334
SLC6A13	-1.095449	6.8366384	-4.726819	1.37E-05	0.0001167	2.7518134
CTH	-1.093745	5.0701392	-5.894364	1.71E-07	3.02E-06	6.9871689
KHK	-1.093265	5.8096926	-5.233365	2.12E-06	2.44E-05	4.5472368
ANPEP	-1.089711	12.687836	-2.80509	0.0067225	0.0205215	-3.081771
TGFBR3	-1.08867	13.274426	-5.391143	1.17E-06	1.49E-05	5.1209147
SELL	-1.088595	5.2178326	-5.450362	9.38E-07	1.23E-05	5.337777
SLC27A2	-1.084785	8.184869	-2.805724	0.0067108	0.0204878	-3.080191
GSTA5	-1.076672	5.0143151	-6.49029	1.67E-08	4.41E-07	9.2515471
AQP7P3	-1.074487	6.3081057	-5.27379	1.82E-06	2.15E-05	4.6936336
RHCG	-1.073928	4.852038	-4.547282	2.60E-05	0.0001987	2.1351093
SNHG8	-1.073245	13.295569	-6.0262	1.03E-07	1.98E-06	7.4838298
ABCC2	-1.072558	5.6798282	-4.619789	2.01E-05	0.0001606	2.3828046
MED11	-1.069873	6.7723577	-8.629822	3.39E-12	4.70E-10	17.547763
HIST1H1C	-1.065737	10.803665	-8.126475	2.50E-11	2.20E-09	15.597165
OTTHUMG00(	-1.065084	5.1213556	-3.879639	0.0002572	0.0013463	-0.046783
EXPH5	-1.064637	11.353164	-3.182164	0.0022914	0.0084106	-2.09483
FTLP3	-1.063887	17.142803	-7.692671	1.41E-10	8.74E-09	13.909957
GADD45B	-1.063313	7.8374623	-5.735184	3.16E-07	4.98E-06	6.3914324
CNTN1	-1.062343	8.4754625	-5.531235	6.90E-07	9.56E-06	5.63522
NAPSA	-1.061292	5.8170121	-5.375724	1.24E-06	1.56E-05	5.0645846
PRAP1	-1.059298	7.9455541	-4.611367	2.07E-05	0.0001647	2.3539348
GK	-1.058249	8.3119226	-4.074607	0.0001341	0.0007881	0.570484
NLK	-1.058046	13.307649	-5.423387	1.04E-06	1.34E-05	5.2388935
ANXA9	-1.057694	6.406141	-7.424284	4.10E-10	2.06E-08	12.86562
NR4A1	-1.054457	5.298542	-6.706974	7.09E-09	2.14E-07	10.085013
CETP	-1.053621	6.9905285	-5.520269	7.19E-07	9.89E-06	5.5948036
FUT6	-1.052888	6.4168734	-4.830226	9.41E-06	8.57E-05	3.1119929
PAIP2B	-1.05055	6.9502631	-5.841951	2.09E-07	3.54E-06	6.790517
ATP5D	-1.050364	11.422428	-8.056909	3.30E-11	2.69E-09	15.326839
MXD1	-1.046641	7.25698	-9.772988	3.86E-14	1.34E-11	21.917558
AKR7A2	-1.041957	7.3204279	-8.413983	7.98E-12	9.03E-10	16.712682
KLK7	-1.041474	11.826013	-3.776643	0.0003604	0.0017885	-0.365534
TST	-1.040238	8.1858139	-8.490398	5.89E-12	7.17E-10	17.008598
CLYBL	-1.038926	7.1600123	-6.657331	8.63E-09	2.53E-07	9.8936967

CLDN8	-1.038707	5.63609	-3.247955	0.0018838	0.0071437	-1.913404
TMEM178A	-1.037959	8.5796782	-4.068422	0.0001369	0.0008023	0.5506344
NAP1L2	-1.037285	6.4011236	-7.014895	2.09E-09	7.73E-08	11.275602
C9orf66	-1.036307	4.5230841	-6.255625	4.19E-08	9.44E-07	8.3543083
TSPYL5	-1.032406	9.8027448	-5.964942	1.30E-07	2.39E-06	7.2527083
SNORA47	-1.03223	5.6543859	-6.299505	3.53E-08	8.20E-07	8.521597
BST1	-1.02881	8.7496818	-4.583536	2.29E-05	0.0001787	2.258721
STRADB	-1.027725	8.4609764	-7.07534	1.64E-09	6.38E-08	11.509966
ENPEP	-1.027617	14.373321	-5.648135	4.41E-07	6.56E-06	6.0676427
KLK1	-1.026204	5.4531761	-3.0582	0.0032933	0.0113454	-2.429412
LINC00839	-1.025358	9.7468049	-4.079367	0.000132	0.0007774	0.585774
SNORD46	-1.025264	8.8407684	-3.326706	0.0014855	0.0058777	-1.692819
FMO4	-1.024641	8.2386436	-3.365183	0.0013212	0.0053341	-1.583715
NKG7	-1.0234	9.5776667	-3.819096	0.0003138	0.0015914	-0.234786
SNORD105B	-1.022437	3.966243	-6.730897	6.45E-09	1.98E-07	10.177278
AOX1	-1.021703	8.1681862	-3.557973	0.0007262	0.0032069	-1.02435
SLC17A3	-1.021482	8.4479648	-2.608434	0.0113998	0.0317369	-3.558514
ADH6	-1.021176	5.8998293	-4.490176	3.19E-05	0.0002369	1.9413804
LOC550643	-1.017311	11.121053	-10.49502	2.42E-15	1.92E-12	24.615166
ATP6V0D2	-1.017293	6.3085528	-3.450212	0.001017	0.0042664	-1.339585
ABRACL	-1.016936	8.4646269	-7.261452	7.84E-10	3.48E-08	12.232545
AGPAT9	-1.016534	6.3677641	-5.575085	5.83E-07	8.29E-06	5.7970914
TMEM207	-1.013409	4.952339	-3.657874	0.0005287	0.0024596	-0.72647
USP46	-1.01327	9.0076508	-5.457438	9.13E-07	1.20E-05	5.3637462
ACE2	-1.011845	7.770401	-2.639371	0.0105075	0.0296913	-3.485326
ZBED5-AS1	-1.010995	8.9073956	-8.477172	6.21E-12	7.44E-10	16.957399
ZNRF3	-1.008357	9.5624521	-5.942191	1.42E-07	2.56E-06	7.1670205
USH1C	-1.007791	5.8766952	-5.788975	2.57E-07	4.19E-06	6.5922372
C15orf59	-1.007487	9.6897989	-3.586467	0.0006637	0.0029768	-0.939933
UQCR10	-1.007483	11.158144	-9.845958	2.91E-14	1.10E-11	22.19266
OTTHUMG000(	-1.007087	6.9886608	-3.943558	0.0002081	0.00113	0.1536246
AS3MT	-1.006901	7.9418823	-6.683679	7.77E-09	2.33E-07	9.995212
C1orf192	-1.006532	7.2886882	-6.370506	2.67E-08	6.54E-07	8.7927744
GPX3	-1.003302	15.435739	-5.158413	2.81E-06	3.09E-05	4.2769278
KL	-1.001605	10.750798	-4.360875	5.02E-05	0.0003449	1.5073053
HSD11B2	-1.000484	5.6512157	-3.027051	0.0036027	0.0121966	-2.51196
ARG2	-0.998956	7.5264261	-6.861595	3.84E-09	1.29E-07	10.682091
OTTHUMG000(	-0.998952	6.4060339	-4.76799	1.18E-05	0.0001028	2.8947966
ACSM5	-0.998556	5.5085425	-6.92012	3.05E-09	1.06E-07	10.908511
FPR2	-0.994684	4.0015548	-6.632889	9.50E-09	2.74E-07	9.7995782
SUCLG1	-0.993377	9.4799769	-5.702837	3.58E-07	5.52E-06	6.2709411
GMNC	-0.989367	5.2358274	-5.87336	1.86E-07	3.22E-06	6.9083066
GPR27	-0.989353	6.6814136	-5.337547	1.44E-06	1.76E-05	4.9253557
FAM134B	-0.988881	10.106177	-6.606846	1.05E-08	2.99E-07	9.6993491
RGN	-0.988704	5.7871266	-6.01731	1.06E-07	2.02E-06	7.4502525
TMEM244	-0.988557	5.4012038	-3.552586	0.0007386	0.0032572	-1.04026
MYCT1	-0.988438	10.916015	-4.690199	1.56E-05	0.0001305	2.6251147
CCDC91	-0.988089	11.01175	-4.943473	6.22E-06	6.01E-05	3.5103659
ACO2	-0.987965	8.5378257	-6.539319	1.37E-08	3.73E-07	9.4397555
IL1RL1	-0.987777	10.513921	-2.93485	0.0046846	0.0152028	-2.75262
C10orf32	-0.982712	9.933201	-8.297702	1.27E-11	1.27E-09	16.261894

TSHZ1	-0.980041	9.2761597	-5.006405	4.93E-06	4.95E-05	3.7334293
MGAM	-0.979296	6.1690977	-3.676561	0.000498	0.002343	-0.670161
ADI1	-0.978857	8.5625641	-9.104172	5.23E-13	1.05E-10	19.373316
DIO1	-0.978715	6.8760852	-3.040808	0.0034629	0.0117954	-2.475578
TNNC1	-0.978711	9.4233356	-2.621876	0.011004	0.0308264	-3.526798
SLC9A3R1	-0.977728	8.0418593	-8.196124	1.90E-11	1.76E-09	15.867671
FGF9	-0.977268	7.595071	-4.628819	1.95E-05	0.0001564	2.4137816
OTTHUMG000	-0.974118	5.9008048	-4.112933	0.0001178	0.0007065	0.6938683
CDKN1B	-0.973039	10.13359	-8.368745	9.55E-12	1.02E-09	16.537376
AIF1	-0.971434	12.660554	-5.071443	3.88E-06	4.04E-05	3.9651768
HSD17B2	-0.971258	4.2905161	-4.55402	2.54E-05	0.0001952	2.158048
DDN	-0.971157	6.5754439	-4.586755	2.26E-05	0.0001769	2.2697187
OR2T10	-0.967722	5.3898526	-3.295194	0.0016343	0.0063628	-1.781528
MRPL16	-0.967498	8.2359416	-9.410527	1.58E-13	4.13E-11	20.543561
APOO	-0.967301	9.7096741	-8.484097	6.04E-12	7.30E-10	16.984206
FBXO21	-0.967036	10.16533	-6.41184	2.27E-08	5.66E-07	8.9509072
OTTHUMG000	-0.965358	12.916718	-4.96358	5.78E-06	5.67E-05	3.581507
HRASLS2	-0.962899	4.3180334	-3.603065	0.0006296	0.0028474	-0.890556
LOC284578	-0.961441	5.8440285	-3.794181	0.0003404	0.001706	-0.311629
OTTHUMG000	-0.960944	6.3294567	-2.980821	0.0041122	0.0136302	-2.633321
OTTHUMG000	-0.960929	13.991425	-7.165969	1.15E-09	4.81E-08	11.861667
CYP27B1	-0.960333	4.5898015	-7.341417	5.70E-10	2.66E-08	12.543361
CMTM2	-0.959891	4.9146354	-7.407341	4.39E-10	2.17E-08	12.799718
SLC5A2	-0.958743	6.0306833	-5.589148	5.53E-07	7.92E-06	5.8490894
TGFBR2	-0.957294	14.577438	-5.035275	4.44E-06	4.52E-05	3.8361498
SFXN2	-0.955369	6.8042482	-5.216107	2.26E-06	2.58E-05	4.4848661
OTTHUMG000	-0.955184	10.469881	-7.331549	5.93E-10	2.74E-08	12.504997
GHITM	-0.954374	11.624385	-8.702385	2.55E-12	3.79E-10	17.827952
LRRC4	-0.95319	7.2568057	-7.415545	4.24E-10	2.12E-08	12.831628
BBOX1	-0.949754	10.893434	-2.697814	0.0089934	0.0260685	-3.345206
BPHL	-0.949696	7.9117357	-5.811052	2.36E-07	3.92E-06	6.6748076
DMGDH	-0.946658	7.7223482	-3.611891	0.0006122	0.0027834	-0.86424
SLC5A10	-0.944782	6.9893407	-4.945178	6.18E-06	5.98E-05	3.5163941
ABCC6P2	-0.944666	5.7893938	-4.958709	5.88E-06	5.76E-05	3.5642606
PRDX6	-0.94232	11.690543	-9.447514	1.37E-13	3.67E-11	20.684318
WT1	-0.940237	13.118262	-4.924408	6.67E-06	6.38E-05	3.4430242
LOC644662	-0.940043	5.3030052	-5.6105	5.10E-07	7.41E-06	5.9281177
NDUFAB1	-0.938692	13.832036	-8.782579	1.86E-12	2.99E-10	18.137247
SYNPO	-0.938224	10.027495	-6.891118	3.42E-09	1.17E-07	10.796283
LRRC19	-0.935141	8.1659415	-2.780487	0.007191	0.0216647	-3.142894
IMPA2	-0.934964	7.926109	-5.170661	2.68E-06	2.98E-05	4.3209985
FRMD3	-0.934428	10.261637	-4.506721	3.01E-05	0.0002251	1.9973827
CDHR5	-0.934371	6.3241841	-4.795154	1.07E-05	9.48E-05	2.9894421
SNORA5A	-0.933546	7.6840887	-3.348422	0.0013906	0.0055654	-1.631347
KLF9	-0.931039	10.738713	-5.464734	8.88E-07	1.18E-05	5.3905307
SPTB	-0.931023	6.4952148	-5.237796	2.09E-06	2.40E-05	4.5632615
CA10	-0.930634	7.2937777	-5.011831	4.84E-06	4.88E-05	3.7527185
PRMT6	-0.92921	8.8377269	-6.493236	1.65E-08	4.37E-07	9.2628501
CPNE8	-0.928357	11.071077	-4.817809	9.84E-06	8.88E-05	3.068559
SLCO4C1	-0.926867	7.4260311	-3.022921	0.0036457	0.0123153	-2.522858
PARD6G	-0.925086	7.7673751	-5.161013	2.78E-06	3.06E-05	4.2862809

CCDC148	-0.924083	7.0389939	-4.730332	1.35E-05	0.0001156	2.7639905
ANK2	-0.922358	7.182358	-5.038127	4.39E-06	4.48E-05	3.8463126
WT1-AS	-0.922037	6.7219428	-6.904823	3.24E-09	1.11E-07	10.849311
LINC00671	-0.921888	6.3070846	-4.666266	1.70E-05	0.0001401	2.5425573
C11orf71	-0.920885	4.8252375	-9.514688	1.05E-13	2.98E-11	20.939644
NUPR1L	-0.91948	5.4127307	-4.009843	0.0001668	0.0009428	0.3634884
RNU5A-1	-0.919289	5.3281493	-6.708486	7.05E-09	2.14E-07	10.090843
AZGP1	-0.916814	6.3539372	-4.174764	9.54E-05	0.0005911	0.8942884
MYOZ1	-0.916175	5.5414038	-5.351694	1.36E-06	1.68E-05	4.9769081
COX8A	-0.914455	17.165707	-9.525388	1.01E-13	2.89E-11	20.980278
RNA5-8SP2	-0.907161	6.07999	-7.420218	4.17E-10	2.08E-08	12.849803
NAMPT	-0.906331	6.0341508	-7.209243	9.65E-10	4.17E-08	12.029713
OTTHUMG00(	-0.904307	6.4191267	-3.75341	0.0003887	0.0019039	-0.436705
LPPR1	-0.904287	6.5101828	-4.697151	1.52E-05	0.0001277	2.6491318
GJA1	-0.903137	11.601244	-4.474807	3.36E-05	0.0002477	1.8894476
SALL1	-0.899226	8.4727415	-7.462744	3.52E-10	1.83E-08	13.01523
GALM	-0.897205	7.4000023	-4.604549	2.12E-05	0.0001682	2.3305852
CRABP1	-0.896494	4.7741411	-5.057109	4.09E-06	4.23E-05	3.9139969
SIRPA	-0.89639	10.384129	-5.323498	1.51E-06	1.84E-05	4.8742108
QDPR	-0.89591	7.1943625	-8.211338	1.78E-11	1.67E-09	15.926741
TMED4	-0.895732	10.499586	-7.83051	8.12E-11	5.65E-09	14.446349
CSRNP1	-0.894615	5.4347548	-5.801278	2.45E-07	4.04E-06	6.6382419
DDTL	-0.893636	5.7143367	-8.705459	2.52E-12	3.78E-10	17.839815
SLC39A5	-0.89351	5.6254989	-5.810766	2.36E-07	3.92E-06	6.673736
ARHGEF26	-0.892816	8.7593613	-3.564119	0.0007122	0.0031569	-1.006177
ATF3	-0.892212	5.4260754	-5.590709	5.50E-07	7.88E-06	5.8548645
OTTHUMG00(	-0.890395	7.7455046	-3.777779	0.0003591	0.0017833	-0.362047
RAB7L1	-0.889483	7.3635008	-6.319147	3.27E-08	7.69E-07	8.5965582
OTTHUMG00(	-0.88642	6.3352703	-5.849963	2.03E-07	3.46E-06	6.8205464
RASL11B	-0.886295	6.5534207	-4.922204	6.72E-06	6.42E-05	3.4352458
TINAG	-0.886135	8.0864693	-3.006159	0.0038252	0.0128275	-2.566974
OTTHUMG00(	-0.88577	7.9220849	-4.326212	5.66E-05	0.0003818	1.3920548
ERVMER34-1	-0.88543	5.3917249	-8.23365	1.63E-11	1.55E-09	16.013352
OTTHUMG00(	-0.885091	8.052201	-6.982383	2.38E-09	8.58E-08	11.149619
SEMA5A	-0.882568	12.885374	-3.798376	0.0003358	0.0016857	-0.298711
EPB41L5	-0.88248	12.161073	-5.020481	4.68E-06	4.75E-05	3.7834839
MACROD1	-0.881518	5.0150482	-9.161942	4.17E-13	9.02E-11	19.59457
NME3	-0.881443	6.3823972	-9.129223	4.74E-13	9.97E-11	19.469289
ST6GALNAC2	-0.8808	7.4139577	-5.094997	3.56E-06	3.75E-05	4.0493994
PM20D2	-0.8786	8.2044128	-7.420633	4.16E-10	2.08E-08	12.851418
LOC145837	-0.878127	4.4235787	-9.420693	1.52E-13	4.01E-11	20.582263
CLPP	-0.878103	9.4891623	-9.568856	8.52E-14	2.57E-11	21.145239
LOC100506(	-0.873362	3.8226121	-5.807391	2.39E-07	3.96E-06	6.6611089
PAQR5	-0.872953	7.8754628	-3.859834	0.0002745	0.0014207	-0.10848
FLRT3	-0.872856	11.800296	-5.045263	4.27E-06	4.38E-05	3.8717435
FRY	-0.872349	12.066847	-3.477044	0.0009357	0.0039716	-1.261695
C11orf1	-0.871444	6.8255592	-10.06732	1.24E-14	6.73E-12	23.023924
AQP7P1	-0.871003	5.7722482	-6.487249	1.69E-08	4.45E-07	9.2398818
SLC23A1	-0.870959	6.8496421	-3.787506	0.0003479	0.0017369	-0.332163
ARRDC2	-0.868792	4.1980475	-4.884961	7.71E-06	7.22E-05	3.3040375
RAET1E	-0.867682	5.6804216	-4.284403	6.54E-05	0.0004307	1.2536902

PXMP2	-0.867028	8.0478623	-5.022947	4.64E-06	4.71E-05	3.7922582
AHCY	-0.866687	5.8820515	-7.132159	1.31E-09	5.35E-08	11.730419
PRDM1	-0.865218	5.9362623	-6.459524	1.88E-08	4.88E-07	9.1335667
MCM6	-0.86519	9.6916348	-4.651492	1.79E-05	0.0001461	2.4916932
ERVFRD-1	-0.864416	6.0157116	-4.600526	2.15E-05	0.0001701	2.3168146
OTTHUMG00(	-0.863583	4.3805748	-8.032447	3.63E-11	2.91E-09	15.231755
TENC1	-0.863502	10.488153	-6.225126	4.72E-08	1.04E-06	8.2381784
HIST1H2AE	-0.863468	9.4551025	-3.574403	0.0006895	0.0030752	-0.975726
NDUFA7	-0.862779	12.83962	-10.26484	5.82E-15	3.68E-12	23.761281
C7orf10	-0.860914	5.8973561	-4.981047	5.42E-06	5.35E-05	3.6434063
NECAB1	-0.860681	8.8490059	-5.193613	2.46E-06	2.77E-05	4.4036906
FBXO17	-0.860224	8.0837154	-8.44357	7.10E-12	8.34E-10	16.827289
MED21	-0.859815	13.531087	-6.766363	5.60E-09	1.76E-07	10.314143
ETNK2	-0.859677	5.545068	-7.175506	1.10E-09	4.69E-08	11.898695
MRGPRF	-0.858896	6.6733666	-7.130191	1.32E-09	5.38E-08	11.722783
FN3K	-0.858704	5.5051816	-7.024639	2.01E-09	7.51E-08	11.313371
HIGD2A	-0.858525	12.083389	-8.125659	2.51E-11	2.20E-09	15.593996
MME-AS1	-0.857815	12.930186	-3.705852	0.0004533	0.0021664	-0.581538
ARRDC4	-0.856761	13.001504	-2.766956	0.0074612	0.0223611	-3.176331
ASPDH	-0.856573	5.8990346	-6.737321	6.29E-09	1.94E-07	10.202061
DISP1	-0.855228	8.7770603	-4.75002	1.26E-05	0.0001087	2.8323206
SLC45A1	-0.854497	6.9638407	-5.609625	5.11E-07	7.42E-06	5.9248784
FAH	-0.854406	6.937368	-6.758751	5.78E-09	1.81E-07	10.284761
RRAGD	-0.853458	7.5081721	-7.14392	1.25E-09	5.17E-08	11.776068
CXCL14	-0.853353	11.500809	-2.493121	0.0153653	0.0406179	-3.825188
SH3BGRL2	-0.851534	15.441252	-4.261348	7.09E-05	0.0004603	1.1776954
RARRES2	-0.851505	11.958577	-6.150622	6.32E-08	1.32E-06	7.9550017
APOD	-0.851358	15.877561	-4.807469	1.02E-05	9.16E-05	3.0324282
SAPCD1-AS1	-0.850501	6.8417785	-5.10168	3.47E-06	3.68E-05	4.0733272
OTTHUMG00(	-0.850501	6.8417785	-5.10168	3.47E-06	3.68E-05	4.0733272
OTTHUMG00(	-0.850501	6.8417785	-5.10168	3.47E-06	3.68E-05	4.0733272
OTTHUMG00(	-0.850501	6.8417785	-5.10168	3.47E-06	3.68E-05	4.0733272
OTTHUMG00(	-0.850501	6.8417785	-5.10168	3.47E-06	3.68E-05	4.0733272
PTPRO	-0.848452	14.546969	-3.675135	0.0005003	0.0023516	-0.674465
PHYH	-0.847482	7.9194257	-6.568026	1.23E-08	3.39E-07	9.5500612
PRLR	-0.846855	5.9698185	-5.373605	1.25E-06	1.57E-05	5.056849
ATP6V1F	-0.845093	10.212936	-9.014273	7.45E-13	1.37E-10	19.028515
PPDPF	-0.844631	7.2964028	-7.410637	4.33E-10	2.14E-08	12.812537
NQO2	-0.84342	6.2373882	-5.271032	1.84E-06	2.17E-05	4.6836315
HSD17B8	-0.843264	7.5433603	-7.924303	5.59E-11	4.13E-09	14.81123
NFASC	-0.840512	10.843951	-3.591871	0.0006524	0.0029314	-0.923873
SHMT1	-0.836768	7.8147395	-4.437831	3.83E-05	0.0002762	1.7648748
MIB1	-0.834055	11.56671	-4.184164	9.24E-05	0.0005755	0.9249022
ACAT1	-0.832679	8.9696413	-4.210221	8.45E-05	0.0005342	1.0099621
OAZ1	-0.831265	14.227766	-7.502444	3.00E-10	1.60E-08	13.169686
SLC23A3	-0.830712	5.6807028	-4.83717	9.17E-06	8.39E-05	3.1363069
IDH2	-0.829627	8.3415141	-5.963282	1.31E-07	2.40E-06	7.2464529
WASL	-0.829224	13.011352	-6.508744	1.55E-08	4.13E-07	9.3223597
SLC2A12	-0.828802	9.8301116	-3.385262	0.0012425	0.0050567	-1.526438
MAGI2	-0.82853	9.1025805	-3.567966	0.0007036	0.0031275	-0.994795
OTTHUMG00(	-0.828206	5.7795516	-3.742028	0.0004033	0.0019627	-0.471472

VPS28	-0.827524	10.25871	-9.543364	9.41E-14	2.74E-11	21.048517
APOE	-0.827353	5.83994	-5.925648	1.52E-07	2.71E-06	7.1047699
CRYAB	-0.826523	8.9201298	-9.329994	2.16E-13	5.39E-11	20.236683
NDUFB7	-0.826303	11.993916	-5.936067	1.46E-07	2.62E-06	7.143972
SNORA79	-0.824984	7.4792054	-3.002421	0.0038664	0.0129486	-2.57679
GOT1	-0.8248	8.1262087	-5.720822	3.34E-07	5.22E-06	6.3379134
ACOX2	-0.824239	6.5869584	-5.719069	3.36E-07	5.24E-06	6.3313827
OGDHL	-0.824172	6.225217	-5.016397	4.76E-06	4.81E-05	3.7689562
OTTHUMG000	-0.823083	6.8911377	-5.911077	1.60E-07	2.85E-06	7.0499768
BCAM	-0.822984	11.384902	-4.096487	0.0001245	0.0007412	0.6408457
HIST1H2AC	-0.821378	9.7243772	-7.650627	1.66E-10	9.92E-09	13.746329
RABAC1	-0.821062	14.226364	-8.517482	5.29E-12	6.65E-10	17.113412
CXCR4	-0.820375	6.9026464	-4.176339	9.49E-05	0.0005884	0.8994145
PROK2	-0.818596	5.1053698	-9.310809	2.33E-13	5.71E-11	20.163495
USP46-AS1	-0.818273	6.3398867	-6.065582	8.80E-08	1.74E-06	7.6327201
QPRT	-0.817438	7.4057438	-6.428942	2.12E-08	5.37E-07	9.0163924
CYP4F3	-0.817407	6.1520461	-4.084278	0.0001298	0.0007662	0.601557
C5AR1	-0.816553	5.9319711	-4.234121	7.78E-05	0.0004977	1.0882353
MTHFS	-0.816459	6.2460098	-10.60184	1.62E-15	1.56E-12	25.009335
C1orf21	-0.81618	7.1283467	-4.65058	1.80E-05	0.0001466	2.488557
TXN2	-0.81511	7.8706333	-9.703362	5.05E-14	1.64E-11	21.654579
C6orf57	-0.814942	6.9775077	-8.640337	3.26E-12	4.59E-10	17.588383
ZNF124	-0.814337	8.0103695	-6.165819	5.96E-08	1.26E-06	8.0127036
KLHL42	-0.811789	11.146731	-4.574764	2.36E-05	0.0001832	2.2287693
LNX2	-0.808822	7.9395062	-6.158623	6.13E-08	1.29E-06	7.9853758
OTTHUMG000	-0.805781	3.9842502	-5.356577	1.34E-06	1.66E-05	4.9947145
MYLIP	-0.80545	11.604052	-4.63953	1.87E-05	0.0001515	2.450565
CPPED1	-0.804918	6.914583	-10.72338	1.02E-15	1.06E-12	25.456277
CPNE4	-0.80415	4.8308652	-4.992474	5.19E-06	5.17E-05	3.6839497
MAFB	-0.803256	8.9661503	-5.458766	9.08E-07	1.20E-05	5.3686191
OTTHUMG000	-0.803209	4.7942518	-4.429211	3.95E-05	0.0002831	1.7359103
SNORA24	-0.803039	6.486717	-3.581485	0.0006742	0.0030195	-0.954723
WDR83OS	-0.802626	11.594585	-7.861956	7.17E-11	5.14E-09	14.568696
ENPP6	-0.802406	4.6801667	-4.69766	1.52E-05	0.0001275	2.6508914
PNPLA4	-0.801417	7.3551093	-4.851185	8.72E-06	8.04E-05	3.1854212
PEBP1	-0.798471	15.236923	-8.965353	9.03E-13	1.61E-10	18.840637
TNNT2	-0.797662	8.280051	-3.52693	0.0008006	0.0034874	-1.115818
STX11	-0.797392	5.7752962	-5.08225	3.73E-06	3.91E-05	4.0037986
PPAP2B	-0.797127	12.702141	-4.522342	2.84E-05	0.0002146	2.0503533
FLJ22763	-0.796973	4.9334359	-3.9378	0.0002121	0.0011476	0.1354911
SOWAHC	-0.795523	6.7748297	-5.867259	1.90E-07	3.28E-06	6.8854123
LGMN	-0.795	12.452061	-3.950063	0.0002037	0.0011087	0.1741307
PRDX2	-0.794352	8.1088836	-8.011727	3.95E-11	3.09E-09	15.151201
PLA2G16	-0.794138	10.858355	-6.650513	8.86E-09	2.59E-07	9.8674363
LGALS2	-0.793551	8.4284469	-4.295782	6.29E-05	0.0004163	1.2912777
H1FX	-0.793127	7.4394684	-7.263734	7.77E-10	3.46E-08	12.241412
HOGA1	-0.792629	5.7142431	-5.301971	1.64E-06	1.97E-05	4.7959314
SUCLG2	-0.792618	11.007422	-6.944595	2.76E-09	9.74E-08	11.003264
FAM127B	-0.791472	10.253677	-9.182114	3.85E-13	8.51E-11	19.671762
LMAN2	-0.791296	10.094618	-8.58902	3.99E-12	5.38E-10	17.390086
RSPH3	-0.791001	8.9300392	-5.845052	2.07E-07	3.51E-06	6.8021364

THSD7A	-0.789155	11.230674	-3.022237	0.0036529	0.012334	-2.524662
SLC16A12	-0.788298	6.5176928	-3.142887	0.0025729	0.0092469	-2.201878
FOXC1	-0.785924	7.480793	-5.887062	1.76E-07	3.09E-06	6.9597441
CLCNKB	-0.784784	7.0252744	-2.987233	0.0040377	0.013428	-2.616569
LRP2	-0.784735	11.283913	-2.403833	0.0192481	0.0488755	-4.024918
TMOD2	-0.783958	9.2934236	-5.623014	4.86E-07	7.11E-06	5.9744811
PLCE1	-0.783444	14.197143	-2.871557	0.005594	0.0176301	-2.914584
ANKS1B	-0.782573	4.9072828	-8.471673	6.35E-12	7.55E-10	16.936109
HIST2H2AB	-0.781478	6.5687864	-5.698679	3.64E-07	5.60E-06	6.2554695
ANGPTL1	-0.781052	5.4794184	-6.477482	1.75E-08	4.60E-07	9.2024185
SCGB1D2	-0.77987	6.2690515	-3.020805	0.003668	0.0123726	-2.528437
CLEC3B	-0.779463	11.062599	-4.067084	0.0001376	0.0008055	0.5463421
OXER1	-0.778567	5.2765266	-5.955441	1.35E-07	2.46E-06	7.216914
OTTHUMG000	-0.777544	5.4190433	-4.318359	5.82E-05	0.0003907	1.3660107
MUT	-0.777404	8.9573949	-8.002197	4.10E-11	3.18E-09	15.114147
MT1CP	-0.777187	8.1765031	-5.55241	6.36E-07	8.93E-06	5.7133338
MRPS18A	-0.77718	11.439233	-6.981155	2.39E-09	8.60E-08	11.14486
NOX4	-0.776904	8.5482243	-3.817577	0.0003154	0.0015976	-0.239477
GZMH	-0.776288	6.2385498	-3.376765	0.0012752	0.0051728	-1.550706
BNIP3	-0.776115	10.141694	-6.564457	1.24E-08	3.44E-07	9.5363465
ZC3H7B	-0.77574	8.0980233	-6.208599	5.04E-08	1.10E-06	8.1752972
LINC00493	-0.775081	13.952049	-8.298664	1.26E-11	1.27E-09	16.265624
MIR1184-2	-0.77502	13.459785	-6.767427	5.58E-09	1.76E-07	10.31825
MIR1184-1	-0.77502	13.459785	-6.767427	5.58E-09	1.76E-07	10.31825
KIRREL	-0.774691	11.790701	-4.662622	1.72E-05	0.0001416	2.5300038
ARSE	-0.773689	6.9750536	-3.622898	0.0005912	0.0027011	-0.831365
DHRS4-AS1	-0.772952	7.5738969	-4.847368	8.84E-06	8.13E-05	3.1720388
ACOT13	-0.772902	7.9677915	-6.460841	1.87E-08	4.86E-07	9.1386147
UBXN6	-0.772736	8.7346908	-9.733952	4.49E-14	1.51E-11	21.770176
SPATA18	-0.772557	6.9258084	-3.608064	0.0006197	0.0028112	-0.875655
SMPDL3A	-0.77162	7.2035631	-5.21533	2.27E-06	2.58E-05	4.482061
CAT	-0.771445	10.989733	-5.08876	3.64E-06	3.83E-05	4.0270836
RNF114	-0.770999	12.886757	-7.374896	4.99E-10	2.38E-08	12.673537
GIMAP7	-0.767263	11.475209	-4.149471	0.000104	0.000636	0.8121012
MME	-0.76653	16.575202	-4.308988	6.01E-05	0.0004013	1.3349671
RAB11B	-0.766216	9.6326695	-10.27492	5.60E-15	3.68E-12	23.798807
CYFIP2	-0.766112	7.7998352	-5.168109	2.71E-06	3.00E-05	4.3118129
PRDM5	-0.765552	6.7606244	-5.019597	4.70E-06	4.76E-05	3.7803384
PARD6B	-0.765361	10.143937	-6.645864	9.03E-09	2.63E-07	9.8495354
FGF14-AS2	-0.764776	6.4118054	-5.053802	4.14E-06	4.27E-05	3.902196
CITED2	-0.763627	10.937527	-4.998945	5.07E-06	5.06E-05	3.7069275
ILF3-AS1	-0.763095	5.1312223	-9.715724	4.82E-14	1.60E-11	21.701303
CMTM8	-0.762501	7.8935105	-5.34167	1.41E-06	1.74E-05	4.940376
RMST	-0.761709	5.6293766	-4.466306	3.47E-05	0.0002542	1.860762
ADAMTS19	-0.761266	7.9891133	-4.218229	8.22E-05	0.0005215	1.0361616
CCL14	-0.760036	6.1415513	-4.168667	9.74E-05	0.0006021	0.8744518
ITGA8	-0.759686	10.759238	-3.170062	0.0023749	0.0086563	-2.127915
CTSH	-0.758628	8.1206139	-3.823293	0.0003095	0.0015735	-0.22181
SLC37A4	-0.758564	6.6176616	-5.977093	1.24E-07	2.31E-06	7.2985061
C14orf37	-0.757156	15.008177	-3.115774	0.0027857	0.0099001	-2.275214
CEACAM1	-0.756369	10.513991	-3.372957	0.0012902	0.0052273	-1.561568

GPR137B	-0.755686	9.3451964	-4.751722	1.25E-05	0.0001081	2.8382328
RPS6KA6	-0.755303	6.553738	-5.549038	6.44E-07	9.01E-06	5.7008884
ALDH7A1	-0.754871	8.886941	-6.337206	3.04E-08	7.28E-07	8.6655174
KANSL1-AS1	-0.754459	8.1466841	-5.551005	6.40E-07	8.97E-06	5.708148
MRPS36	-0.753785	10.017756	-8.908797	1.13E-12	1.96E-10	18.623226
FAM167B	-0.753102	6.4393685	-4.63913	1.88E-05	0.0001517	2.4491919
ARNTL	-0.752682	5.3604321	-9.098096	5.36E-13	1.07E-10	19.35003
SDHB	-0.752438	7.6202213	-9.897618	2.38E-14	9.96E-12	22.3871
ALDH3A2	-0.751748	8.4626903	-5.249711	2.00E-06	2.32E-05	4.6063801
TULP4	-0.750776	9.4702682	-7.11141	1.42E-09	5.69E-08	11.649898
ID1	-0.749776	11.245602	-4.008849	0.0001673	0.0009455	0.3603237
ADRBK2	-0.749308	7.8796948	-5.2679	1.87E-06	2.19E-05	4.6722755
TMEM98	-0.748227	9.552542	-6.096907	7.79E-08	1.57E-06	7.7513132
TMEM160	-0.74766	8.5278346	-8.882928	1.25E-12	2.13E-10	18.523707
HSPA1L	-0.7469	4.8246049	-10.72128	1.03E-15	1.06E-12	25.448568
BOK	-0.746769	7.5755162	-5.831669	2.18E-07	3.66E-06	6.7519933
COTL1	-0.746284	9.7504187	-4.436303	3.85E-05	0.0002774	1.75974
CYP3A7	-0.746282	4.6815154	-6.042385	9.64E-08	1.88E-06	7.5449914
ANG	-0.745933	7.4114398	-9.344773	2.04E-13	5.21E-11	20.29304
SERPINA5	-0.744869	5.9777361	-4.187432	9.14E-05	0.00057	0.9355539
FAM96B	-0.744411	10.523468	-9.05474	6.35E-13	1.24E-10	19.183797
TRAV39	-0.743952	4.3400356	-4.096644	0.0001245	0.0007409	0.6413482
FAM65A	-0.743126	11.175307	-4.190056	9.05E-05	0.0005663	0.9441103
VTI1B	-0.742655	10.383314	-8.920622	1.08E-12	1.89E-10	18.668701
FGF1	-0.742565	13.866044	-4.409315	4.23E-05	0.0002995	1.6691634
MYL9	-0.742467	16.910525	-6.009074	1.10E-07	2.08E-06	7.4191565
RERG	-0.742417	9.8742126	-5.679028	3.92E-07	5.96E-06	6.1823857
F3	-0.741122	10.615289	-3.332938	0.0014577	0.005789	-1.675207
MIR4444-1	-0.74028	5.1774907	-5.883126	1.79E-07	3.13E-06	6.9449666
CA12	-0.738773	9.886791	-2.841374	0.0060828	0.018863	-2.990875
OTTHUMG000	-0.738667	4.7609148	-3.647509	0.0005466	0.0025256	-0.757623
EIF4EBP2	-0.738326	12.415123	-9.899435	2.37E-14	9.96E-12	22.393933
ZDHHC6	-0.73815	15.198431	-3.068755	0.0031942	0.011075	-2.4013
GCSHP3	-0.737577	8.2556884	-6.758395	5.78E-09	1.81E-07	10.283386
OTTHUMG000	-0.737465	6.4452662	-3.98768	0.0001796	0.0010016	0.2930896
SNTA1	-0.737427	5.9434549	-7.184639	1.06E-09	4.54E-08	11.93416
JMJD8	-0.736932	8.6235615	-5.962651	1.31E-07	2.40E-06	7.2440747
OTTHUMG000	-0.736672	3.4691831	-5.092389	3.59E-06	3.78E-05	4.0400677
PCMTD2	-0.736162	11.509393	-5.308554	1.60E-06	1.93E-05	4.8198573
EMC6	-0.735573	6.3001808	-8.506603	5.53E-12	6.81E-10	17.071316
ACO1	-0.735456	10.377965	-3.674161	0.0005019	0.0023576	-0.677403
GPD1	-0.734534	6.0718972	-4.249509	7.38E-05	0.000476	1.1387584
FAM20B	-0.733862	10.362553	-4.257097	7.19E-05	0.0004658	1.1637082
AGT	-0.733536	5.8419982	-3.603806	0.0006282	0.0028432	-0.888348
TACR3	-0.732905	6.2945275	-3.642148	0.000556	0.0025631	-0.773713
DEPDC7	-0.732069	4.0081398	-6.898837	3.31E-09	1.14E-07	10.82615
MTTP	-0.731843	4.7245356	-4.46517	3.48E-05	0.0002549	1.8569306
NQO1	-0.731698	11.164603	-3.697259	0.000466	0.0022176	-0.607583
ECM2	-0.731484	7.4274008	-5.710189	3.48E-07	5.41E-06	6.2983102
ARHGAP24	-0.729937	6.2060451	-9.915388	2.23E-14	9.56E-12	22.453923
DLG2	-0.729376	6.667313	-4.671673	1.67E-05	0.0001378	2.5611905

STRA13	-0.728688	7.4174841	-8.701935	2.55E-12	3.79E-10	17.826218
MAPT	-0.727451	6.2214697	-7.588407	2.13E-10	1.22E-08	13.50419
SLC2A13	-0.727102	7.4119189	-5.043002	4.31E-06	4.41E-05	3.8636843
H2AFJ	-0.726953	8.3993367	-5.699849	3.62E-07	5.57E-06	6.2598245
ISCA1P1	-0.725928	13.200716	-6.10378	7.59E-08	1.54E-06	7.7773547
PDK2	-0.725893	5.9525836	-9.826496	3.14E-14	1.15E-11	22.119338
MBNL3	-0.7258	7.8516041	-5.633843	4.66E-07	6.86E-06	6.0146226
SLC25A20	-0.725537	6.0890885	-7.110329	1.43E-09	5.71E-08	11.645703
MUC15	-0.725323	5.236961	-4.090413	0.0001271	0.0007537	0.6212904
GTF2H5	-0.724659	8.8720741	-7.744752	1.14E-10	7.53E-09	14.11264
RPP25L	-0.721783	6.5756292	-9.74698	4.27E-14	1.45E-11	21.819381
SEC61B	-0.721282	11.643184	-7.787091	9.66E-11	6.50E-09	14.277399
SIK1	-0.721243	5.7402244	-4.624361	1.98E-05	0.0001585	2.3984847
DHTKD1	-0.720721	7.4011033	-5.443754	9.61E-07	1.26E-05	5.3135397
LAMTOR4	-0.72033	6.2563339	-10.0045	1.58E-14	7.63E-12	22.788543
ODC1	-0.720266	13.391807	-6.635562	9.40E-09	2.72E-07	9.809868
TALDO1	-0.720257	11.708138	-10.30228	5.05E-15	3.47E-12	23.900575
OTTHUMG00(	-0.720157	4.0134938	-4.067112	0.0001375	0.0008055	0.5464313
LOC401164	-0.719861	4.3565672	-5.226911	2.18E-06	2.49E-05	4.523902
SDC1	-0.719707	6.5276382	-6.228627	4.66E-08	1.04E-06	8.2515039
GSTM3	-0.719195	9.4153056	-4.509288	2.98E-05	0.0002235	2.0060801
WDFY3-AS2	-0.717613	5.450673	-6.345539	2.95E-08	7.11E-07	8.6973483
GLIPR1L2	-0.717072	5.290687	-3.876951	0.0002595	0.0013571	-0.055166
TTC38	-0.716784	6.6510087	-5.143038	2.97E-06	3.24E-05	4.2216643
EMR3	-0.716241	4.1250559	-7.279474	7.30E-10	3.28E-08	12.302578
DCTPP1	-0.715629	6.4446461	-5.362943	1.30E-06	1.62E-05	5.0179364
OR2T6	-0.715015	4.5538697	-3.774774	0.0003626	0.0017974	-0.371268
ASB15	-0.714386	4.176302	-5.686074	3.82E-07	5.83E-06	6.2085815
CCBE1	-0.71427	6.6558907	-4.679286	1.62E-05	0.0001345	2.5874446
IVD	-0.714209	6.6455267	-8.406105	8.23E-12	9.25E-10	16.682161
NFKBIA	-0.713635	12.877989	-5.656727	4.27E-07	6.39E-06	6.099535
SPOCK2	-0.713433	11.47288	-3.735185	0.0004123	0.002001	-0.492345
FOLH1	-0.712831	6.2529513	-3.482018	0.0009213	0.0039197	-1.247211
CDH16	-0.712697	7.0962172	-2.92659	0.0047949	0.0154943	-2.773907
TM7SF2	-0.712693	6.5957797	-7.904852	6.04E-11	4.43E-09	14.735572
SPRYD4	-0.712051	7.6987882	-8.920773	1.08E-12	1.89E-10	18.669281
LOC729970	-0.711973	7.2370833	-4.797573	1.06E-05	9.42E-05	2.9978809
OTTHUMG00(	-0.711656	7.0714826	-3.609473	0.000617	0.0028007	-0.871455
INSR	-0.710793	7.1943154	-5.611185	5.08E-07	7.39E-06	5.9306573
MYL3	-0.710747	6.5602656	-6.331446	3.11E-08	7.41E-07	8.6435184
FAM43A	-0.710703	10.480389	-3.692892	0.0004726	0.0022438	-0.620806
OTTHUMG00(	-0.710566	3.8214646	-2.933803	0.0046984	0.015243	-2.755319
LINC00657	-0.710026	14.37733	-8.212397	1.78E-11	1.66E-09	15.93085
BST2	-0.709449	13.330372	-4.40666	4.27E-05	0.0003015	1.6602697
TIMM13	-0.708138	8.6759425	-6.509432	1.55E-08	4.13E-07	9.3249987
LOC1002893	-0.707328	6.6852195	-4.804356	1.03E-05	9.24E-05	3.021559
RNA5-8S5	-0.706893	13.414705	-6.885019	3.50E-09	1.19E-07	10.77269
BMP7	-0.70687	6.413461	-5.172285	2.67E-06	2.96E-05	4.3268439
SDHD	-0.706625	11.448585	-7.712875	1.30E-10	8.21E-09	13.988583
OTTHUMG00(	-0.706435	4.3783561	-5.166117	2.73E-06	3.01E-05	4.3046411
NDUFV1	-0.704528	8.4180903	-6.424906	2.16E-08	5.44E-07	9.0009342

HADH	-0.703713	6.9404184	-5.261145	1.91E-06	2.24E-05	4.6477958
FUOM	-0.703478	5.981803	-9.567801	8.55E-14	2.57E-11	21.141234
NECAP1	-0.703382	8.8710848	-6.15931	6.11E-08	1.29E-06	7.9879848
RAMP3	-0.702876	9.75783	-3.762644	0.0003772	0.0018566	-0.408451
IP6K3	-0.702244	4.5677877	-5.43595	9.90E-07	1.29E-05	5.2849262
WNT3	-0.70188	6.3785008	-3.846971	0.0002864	0.0014756	-0.148448
EMX2	-0.701627	6.1454008	-3.920849	0.0002244	0.0011991	0.0822029
FAM81A	-0.70105	7.7007926	-3.576981	0.0006839	0.0030551	-0.968085
LDOC1	-0.700363	7.4153025	-6.658789	8.58E-09	2.52E-07	9.8993129
NFIC	-0.700222	10.112113	-9.760188	4.06E-14	1.39E-11	21.869246
TMEM109	-0.699799	11.226718	-6.152286	6.28E-08	1.31E-06	7.961317
TMEM256	-0.699697	9.5741131	-6.665229	8.36E-09	2.48E-07	9.9241214
PRUNE	-0.69967	8.3064257	-9.865509	2.70E-14	1.06E-11	22.266278
RNF185-AS1	-0.699047	10.615359	-6.289326	3.67E-08	8.47E-07	8.4827703
TPMT	-0.697991	9.2339182	-4.382706	4.65E-05	0.0003231	1.5801396
SLC22A12	-0.697605	5.8547157	-4.146586	0.000105	0.0006409	0.8027469
RARRES3	-0.697159	10.217403	-5.817679	2.30E-07	3.84E-06	6.6996077
ARHGEF37	-0.696504	6.6771379	-4.523155	2.84E-05	0.0002141	2.053114
NCF1B	-0.695263	5.750142	-3.566429	0.0007071	0.0031396	-0.999344
TECRP1	-0.695104	12.980259	-6.505623	1.57E-08	4.18E-07	9.3103809
MRPL50	-0.695056	8.2470769	-6.634401	9.44E-09	2.73E-07	9.8053969
DNAJC12	-0.694582	5.8835982	-4.060711	0.0001406	0.000819	0.525909
TMEM14A	-0.694316	12.966789	-6.096793	7.80E-08	1.57E-06	7.7508839
ZMYND12	-0.693357	5.5093348	-7.220833	9.22E-10	4.01E-08	12.074734
DPP4	-0.693351	9.2267974	-2.638312	0.0105369	0.0297501	-3.487842
CABLES1	-0.693151	8.6797615	-6.082843	8.23E-08	1.65E-06	7.6980516
SRGAP2	-0.690892	12.03578	-2.814495	0.006551	0.0200733	-3.058297
TMEM208	-0.690126	9.2390174	-6.217013	4.88E-08	1.07E-06	8.2073052
FLT1	-0.689686	12.44626	-3.297247	0.0016242	0.0063258	-1.775766
CDA	-0.689272	5.3663789	-5.351265	1.36E-06	1.69E-05	4.9753444
DLL4	-0.689079	6.574949	-4.555508	2.53E-05	0.0001944	2.163116
FREM2	-0.687431	6.0839334	-3.556586	0.0007293	0.0032196	-1.028448
LPCAT2	-0.685822	10.842192	-4.57909	2.32E-05	0.0001809	2.2435367
TAB3-AS2	-0.685716	10.826579	-5.351792	1.36E-06	1.68E-05	4.9772661
NKD1	-0.68547	7.3644456	-3.512642	0.0008373	0.0036252	-1.15774
MRPS35	-0.68537	10.035708	-6.573286	1.20E-08	3.34E-07	9.5702823
ID4	-0.685173	8.7793449	-5.784723	2.61E-07	4.25E-06	6.5763444
C12orf39	-0.685139	5.5740644	-3.726279	0.0004244	0.0020495	-0.519471
AKR1A1	-0.684972	8.0449021	-5.517242	7.27E-07	9.99E-06	5.5836523
SLC13A2	-0.684284	6.0659097	-3.149748	0.0025215	0.0090961	-2.183247
HIBADH	-0.684041	8.944258	-7.144296	1.25E-09	5.17E-08	11.777529
SLC25A5	-0.683702	9.8538336	-5.565224	6.06E-07	8.56E-06	5.7606541
APOOL	-0.68369	9.4694569	-9.341606	2.07E-13	5.23E-11	20.280965
BBS1	-0.683333	8.6894243	-8.607865	3.70E-12	5.02E-10	17.462923
SLC22A11	-0.682424	5.6935551	-4.056928	0.0001424	0.0008282	0.513791
CYP17A1-AS	-0.682163	5.3865559	-5.525311	7.05E-07	9.74E-06	5.613382
SLC25A33	-0.682148	7.7597164	-3.862378	0.0002722	0.0014115	-0.100565
OTTHUMG000	-0.682134	4.3569256	-3.519369	0.0008198	0.0035606	-1.138015
OTTHUMG000	-0.681923	4.4993741	-5.246627	2.02E-06	2.34E-05	4.5952182
GABARAPL1	-0.681195	5.6514548	-8.796405	1.76E-12	2.86E-10	18.190534
AIFM1	-0.681126	9.4783674	-3.94762	0.0002053	0.0011164	0.1664285

LOC389332	-0.680964	4.0608903	-6.22818	4.67E-08	1.04E-06	8.2498022
PNMA1	-0.679627	8.148813	-7.046068	1.85E-09	7.04E-08	11.396446
OCEL1	-0.678884	6.4719051	-9.044641	6.61E-13	1.26E-10	19.145056
KLK6	-0.678436	13.292082	-3.583693	0.0006695	0.003	-0.94817
GOLIM4	-0.678157	14.41773	-4.114486	0.0001172	0.0007034	0.6988818
DNAJB1	-0.677966	7.6422921	-5.050172	4.20E-06	4.32E-05	3.8892497
ARL2	-0.677948	10.9643	-7.491918	3.13E-10	1.66E-08	13.128729
RNA5-8SP5	-0.677461	3.9481621	-5.94628	1.40E-07	2.54E-06	7.1824168
LOC339535	-0.676774	7.6625479	-2.809703	0.0066379	0.0203113	-3.070266
TRAM1L1	-0.676637	7.0938426	-4.496041	3.12E-05	0.0002325	1.96122
C11orf63	-0.676612	7.2006005	-5.459746	9.05E-07	1.20E-05	5.3722159
SLC16A4	-0.676571	11.701945	-3.674733	0.0005009	0.0023539	-0.675679
GBP4	-0.676228	11.145501	-2.901094	0.005151	0.0164504	-2.839333
CHPT1	-0.675764	8.03443	-5.939434	1.44E-07	2.59E-06	7.1566446
OTTHUMG000	-0.675067	4.0750285	-2.879016	0.0054789	0.0173364	-2.895636
MDH2	-0.674211	9.9383059	-7.727034	1.23E-10	7.87E-09	14.043686
SERPINA6	-0.674093	6.1419966	-3.506203	0.0008543	0.003684	-1.176595
MSRB3	-0.673401	7.9743526	-7.028437	1.98E-09	7.41E-08	11.328094
MCCD1	-0.672324	5.6154615	-3.679298	0.0004937	0.0023271	-0.661899
GLDC	-0.672297	5.9000079	-3.123827	0.0027208	0.0097075	-2.25348
MAML2	-0.671368	9.8910803	-8.779969	1.88E-12	3.00E-10	18.127189
NAPEPLD	-0.670498	8.962687	-5.893455	1.72E-07	3.03E-06	6.9837536
CPM	-0.670406	8.1230574	-5.126643	3.16E-06	3.40E-05	4.1628033
OTTHUMG000	-0.670245	6.0823513	-8.502282	5.62E-12	6.90E-10	17.054593
ISOC2	-0.670212	7.1888169	-5.145228	2.95E-06	3.21E-05	4.2295309
C19orf53	-0.670155	13.439128	-7.338155	5.78E-10	2.68E-08	12.53068
NUDT16	-0.669741	5.7117831	-10.14069	9.36E-15	5.36E-12	23.298289
BAIAP2	-0.669566	6.6188821	-6.173945	5.77E-08	1.23E-06	8.0435694
WIPF3	-0.669445	7.6660423	-3.599717	0.0006364	0.0028732	-0.900527
TRPC7-AS1	-0.6691	5.7854516	-3.20435	0.0021455	0.0079773	-2.033944
RNA5-8SP6	-0.669056	3.1981713	-6.317308	3.29E-08	7.74E-07	8.5895377
PTBP3	-0.668664	13.969305	-7.523756	2.76E-10	1.50E-08	13.25261
LOC1001321	-0.667931	4.0695146	-4.066272	0.0001379	0.0008071	0.543737
HEBP1	-0.667272	8.614017	-5.689693	3.76E-07	5.76E-06	6.2220403
OTTHUMG000	-0.666905	4.6872218	-6.163149	6.02E-08	1.27E-06	8.0025631
F11	-0.666063	4.7750375	-4.395645	4.44E-05	0.0003113	1.6233921
CTDSPL	-0.665895	11.002711	-4.364849	4.95E-05	0.0003411	1.5205509
SLC44A2	-0.665811	13.503857	-5.102348	3.46E-06	3.68E-05	4.0757172
MGAT5	-0.66567	12.387018	-2.878571	0.0054857	0.0173491	-2.896767
SNORA59B	-0.665665	5.8114803	-4.140975	0.0001071	0.0006516	0.7845589
ALDH1L1	-0.66536	4.4850249	-7.504144	2.98E-10	1.60E-08	13.1763
RBM43	-0.664892	7.8006584	-6.842844	4.14E-09	1.37E-07	10.609593
BDH2	-0.66486	8.7048072	-4.748158	1.27E-05	0.0001093	2.8258522
LOC1005066	-0.664686	7.6673707	-4.268221	6.92E-05	0.0004509	1.2003277
TMEM37	-0.664099	7.1810664	-5.231282	2.14E-06	2.45E-05	4.5397034
VSIG8	-0.664058	6.4307297	-4.283868	6.56E-05	0.0004313	1.2519236
MCUR1	-0.661995	10.710303	-8.275118	1.38E-11	1.35E-09	16.174275
ZNF322P1	-0.661923	6.9607164	-5.81874	2.29E-07	3.83E-06	6.7035806
ICT1	-0.66181	10.425172	-6.228133	4.67E-08	1.04E-06	8.2496236
UNG	-0.661293	8.0564579	-7.49565	3.08E-10	1.64E-08	13.143252
PCK2	-0.661152	6.7255243	-4.235088	7.76E-05	0.0004964	1.0914066

PAQR7	-0.660335	10.396044	-5.167976	2.71E-06	3.00E-05	4.3113328
OTTHUMG000	-0.660261	11.869478	-3.082029	0.0030735	0.0107252	-2.365847
SLC25A39	-0.659232	7.8922572	-7.690231	1.42E-10	8.76E-09	13.900461
TMEM200C	-0.658987	5.5043641	-5.530291	6.92E-07	9.59E-06	5.6317414
SLC25A10	-0.658584	4.9635862	-5.033269	4.47E-06	4.55E-05	3.8290067
SLITRK2	-0.658538	5.7847889	-4.426732	3.98E-05	0.0002852	1.7275849
OTTHUMG000	-0.658071	8.4452375	-3.874497	0.0002616	0.0013668	-0.06282
FAM138B	-0.657214	6.3826431	-4.089146	0.0001277	0.0007562	0.6172143
SLCO2A1	-0.657087	14.598502	-4.686235	1.58E-05	0.0001322	2.6114267
EIF6	-0.657084	5.5714559	-8.264241	1.45E-11	1.39E-09	16.132072
TMEM245	-0.656826	12.021802	-4.06174	0.0001401	0.0008169	0.5292095
UBL5	-0.656484	13.672897	-7.834903	7.98E-11	5.58E-09	14.463441
SPINT2	-0.655657	9.5175225	-5.886452	1.76E-07	3.10E-06	6.9574534
KCNJ10	-0.655529	5.3621961	-3.167018	0.0023963	0.0087169	-2.136221
HDDC2	-0.655519	9.2472779	-6.767716	5.57E-09	1.76E-07	10.319366
SOCS3	-0.655221	4.6396434	-6.358937	2.80E-08	6.80E-07	8.7485458
PMM1	-0.654649	5.7881679	-9.269942	2.73E-13	6.50E-11	20.007493
MAL2	-0.654306	10.651501	-5.045271	4.27E-06	4.38E-05	3.8717743
SLC28A1	-0.65376	5.6798939	-4.128883	0.0001116	0.0006757	0.7454114
LOC728040	-0.653294	6.0675818	-2.530214	0.0139713	0.0375529	-3.740469
SEMA3B	-0.653023	6.6037448	-6.372444	2.65E-08	6.50E-07	8.8001847
SCP2	-0.652878	9.560202	-7.83149	8.09E-11	5.64E-09	14.450162
SLC17A5	-0.652736	11.19285	-5.413686	1.08E-06	1.38E-05	5.2033742
SPRY4	-0.652607	7.5364482	-5.463963	8.90E-07	1.18E-05	5.3877012
SDR42E1	-0.652338	7.2489056	-3.222179	0.0020346	0.0076273	-1.984795
UGT2B10	-0.6523	4.7158975	-4.122106	0.0001142	0.000689	0.7234961
NR4A3	-0.651447	4.140049	-3.419737	0.0011175	0.0046196	-1.427558
PLBD1	-0.651374	10.254845	-4.786555	1.10E-05	9.74E-05	2.9594548
CCL23	-0.65093	4.939698	-3.127662	0.0026904	0.0096179	-2.243116
SUCO	-0.650137	9.8246752	-6.745841	6.08E-09	1.89E-07	10.234933
TREH	-0.650031	4.8343923	-4.076191	0.0001334	0.0007846	0.5755711
ERO1LB	-0.649723	8.7811584	-6.568867	1.22E-08	3.38E-07	9.5532945
IQGAP2	-0.649616	12.858443	-3.232126	0.0019751	0.0074386	-1.957294
SEMA3G	-0.649015	8.1985244	-3.37005	0.0013017	0.005265	-1.569854
PCYOX1	-0.648622	12.780656	-5.533418	6.84E-07	9.50E-06	5.6432688
NEDD4L	-0.648509	8.7453521	-5.112179	3.34E-06	3.56E-05	4.1109375
OTTHUMG000	-0.648199	9.8560956	-4.369338	4.87E-05	0.0003367	1.5355164
HIST1H2BG	-0.647893	5.5015325	-3.227281	0.0020039	0.0075295	-1.970698
ALDH9A1	-0.647348	9.4508925	-7.935859	5.34E-11	4.00E-09	14.856177
KCNK5	-0.6473	5.2626877	-3.214309	0.0020829	0.0077866	-2.006515
SORT1	-0.647281	12.299472	-4.345243	5.30E-05	0.0003606	1.4552704
OTTHUMG000	-0.647106	7.3712556	-3.459454	0.0009883	0.0041656	-1.312802
LRRC6	-0.647085	8.1546397	-6.963573	2.56E-09	9.13E-08	11.076756
DHRS3	-0.646743	10.391289	-4.956747	5.92E-06	5.78E-05	3.5573191
TCEB2	-0.646588	8.8735634	-10.5742	1.79E-15	1.62E-12	24.907468
FUZ	-0.646065	6.3267546	-9.408457	1.59E-13	4.13E-11	20.535681
SNORD116-8	-0.644223	6.7361179	-3.648554	0.0005447	0.0025183	-0.754484
ATPIF1	-0.643602	11.381215	-6.159913	6.09E-08	1.28E-06	7.9902758
SNORD116-2	-0.643364	4.3014264	-5.714828	3.42E-07	5.32E-06	6.3155852
TLR3	-0.643006	9.5031246	-5.214584	2.28E-06	2.58E-05	4.4793644
SMIM10	-0.64277	8.9456557	-5.109019	3.38E-06	3.60E-05	4.0996141

GMDS	-0.642035	7.709379	-6.530937	1.42E-08	3.84E-07	9.4075628
CRYM	-0.641798	5.1840374	-5.255716	1.95E-06	2.27E-05	4.6281275
MRPS7	-0.64173	10.209326	-5.975702	1.25E-07	2.32E-06	7.2932607
MRPS34	-0.640678	8.0773574	-7.031046	1.96E-09	7.35E-08	11.338205
GRHPR	-0.640663	7.8043707	-7.68489	1.45E-10	8.89E-09	13.879676
DNPH1	-0.640535	7.3827156	-5.968827	1.28E-07	2.36E-06	7.2673499
CLDND2	-0.639845	6.679118	-5.62828	4.76E-07	6.99E-06	5.9939972
OTTHUMG000	-0.6397	5.2584511	-4.154168	0.0001024	0.0006275	0.8273453
DNAL1	-0.639514	7.2530864	-7.072322	1.66E-09	6.45E-08	11.498258
C16orf80	-0.639384	8.6220959	-7.011632	2.12E-09	7.81E-08	11.262956
ENTPD5	-0.638946	8.3868213	-2.989995	0.0040061	0.013336	-2.609346
MXRA8	-0.638909	9.835368	-6.194445	5.33E-08	1.15E-06	8.1214757
NCF1C	-0.638803	5.8609464	-3.837467	0.0002955	0.0015143	-0.177927
MPC2	-0.638632	8.6252077	-6.999288	2.22E-09	8.15E-08	11.215117
FAM65B	-0.638058	6.8341161	-5.468904	8.74E-07	1.17E-05	5.405845
LINC00853	-0.63703	4.8638441	-5.153416	2.86E-06	3.13E-05	4.2589595
ACOT2	-0.636228	7.5936233	-7.154964	1.20E-09	4.98E-08	11.818942
BTBD3	-0.636164	7.7099544	-4.219873	8.17E-05	0.0005189	1.0415429
CNDP2	-0.635895	9.8242689	-5.360893	1.31E-06	1.63E-05	5.0104549
ZNF804A	-0.635734	6.9156882	-2.505893	0.0148717	0.0395194	-3.796131
OTTHUMG000	-0.635581	8.5893461	-4.048274	0.0001466	0.0008478	0.4860912
CSF3R	-0.635236	4.8949656	-6.874548	3.65E-09	1.23E-07	10.732186
HERPUD1	-0.634886	11.856618	-5.553843	6.33E-07	8.88E-06	5.7186258
OTTHUMG000	-0.634732	6.2924034	-4.430953	3.93E-05	0.0002818	1.7417619
PC	-0.634588	5.6925875	-7.389104	4.72E-10	2.29E-08	12.72879
FOXF1	-0.633733	5.7602198	-3.953699	0.0002012	0.0010998	0.1855998
GSTK1	-0.633576	8.8736951	-8.139099	2.38E-11	2.12E-09	15.646207
PVRL2	-0.633555	9.7177338	-4.663777	1.72E-05	0.000141	2.5339834
EPHA7	-0.633294	7.3653477	-3.59975	0.0006363	0.0028732	-0.90043
GALC	-0.633203	9.4430789	-4.367655	4.90E-05	0.0003384	1.5299065
NXPH2	-0.632958	4.389253	-5.579019	5.75E-07	8.19E-06	5.8116309
RGS1	-0.632678	3.7298331	-5.037512	4.40E-06	4.49E-05	3.8441201
THNSL2	-0.632525	6.301029	-4.064384	0.0001388	0.000811	0.5376847
SYAP1	-0.632447	10.397388	-7.71799	1.27E-10	8.09E-09	14.008492
TSPAN2	-0.63219	13.220366	-2.7112	0.008676	0.025305	-3.312771
TMEM88	-0.631398	12.312603	-2.918911	0.0048997	0.0157767	-2.793658
FOSB	-0.630849	4.00871	-6.332096	3.11E-08	7.40E-07	8.6459984
FOXD2-AS1	-0.630754	5.023511	-5.210619	2.31E-06	2.61E-05	4.4650484
NDUFB5	-0.630664	8.3173079	-8.850867	1.42E-12	2.37E-10	18.400306
TRIM6	-0.630605	6.6148725	-3.458863	0.0009901	0.0041722	-1.314517
TPRG1L	-0.630226	10.780155	-6.963742	2.56E-09	9.13E-08	11.077412
OTTHUMG000	-0.629973	5.0769262	-4.128747	0.0001116	0.0006759	0.7449715
OTTHUMG000	-0.629388	4.6601285	-5.278895	1.79E-06	2.12E-05	4.7121488
DEFA4	-0.629257	3.9366046	-3.496204	0.0008814	0.0037852	-1.20583
LOC729080	-0.629098	6.953521	-6.295801	3.58E-08	8.28E-07	8.5074672
CR1L	-0.629017	5.4585748	-4.699043	1.51E-05	0.000127	2.6556704
TMEM106A	-0.629008	8.6921098	-3.579311	0.0006789	0.0030372	-0.961173
RNF181	-0.628884	9.4918248	-7.719962	1.26E-10	8.04E-09	14.016167
SEPW1	-0.628551	9.6952375	-6.771203	5.50E-09	1.74E-07	10.332827
MANEA	-0.627981	9.035959	-6.130312	6.84E-08	1.41E-06	7.877939
CHCHD2P6	-0.627973	9.3971141	-9.857335	2.79E-14	1.06E-11	22.235505

GAS6	-0.626854	6.9141374	-8.092479	2.86E-11	2.43E-09	15.465075
FNBP1L	-0.626517	12.315693	-4.863934	8.32E-06	7.72E-05	3.2301508
PHYHIPL	-0.625798	6.3027057	-4.211148	8.42E-05	0.0005326	1.0129952
BNIP3P1	-0.625421	6.938019	-6.275775	3.87E-08	8.84E-07	8.4310997
ALS2CL	-0.624494	8.0088366	-2.949522	0.0044943	0.0146996	-2.714695
LOC1009968	-0.623906	5.6452905	-4.312379	5.94E-05	0.0003977	1.3461956
GLB1L2	-0.623431	6.1549954	-5.651659	4.35E-07	6.49E-06	6.0807235
XYLT1	-0.623345	5.9488089	-4.954658	5.97E-06	5.82E-05	3.5499244
KLF2	-0.622738	8.8412026	-3.450748	0.0010153	0.0042611	-1.338031
SDHC	-0.622712	11.305861	-8.521643	5.21E-12	6.60E-10	17.129514
PMVK	-0.622195	6.6709405	-6.608603	1.05E-08	2.97E-07	9.7061089
DDIT4L	-0.621804	5.9819679	-4.948297	6.11E-06	5.93E-05	3.5274222
HDHD2	-0.620834	9.8987948	-5.398616	1.14E-06	1.45E-05	5.1482371
NINJ1	-0.620654	7.3458457	-7.525568	2.74E-10	1.50E-08	13.25966
AP1M2	-0.620554	4.6215441	-6.933068	2.89E-09	1.01E-07	10.958633
TMEM167B	-0.620197	8.3199602	-8.515704	5.33E-12	6.67E-10	17.106532
HIPK2	-0.619145	14.546603	-4.499252	3.09E-05	0.0002304	1.9720871
FAM162A	-0.61895	10.150074	-6.061415	8.95E-08	1.77E-06	7.6169536
FECH	-0.618651	6.6336751	-6.785373	5.20E-09	1.66E-07	10.387541
C21orf33	-0.618606	5.8873257	-7.699727	1.37E-10	8.54E-09	13.937418
LAMTOR5	-0.61836	6.4046702	-6.635834	9.39E-09	2.72E-07	9.8109138
NAMPTL	-0.618013	6.0396215	-4.832738	9.32E-06	8.51E-05	3.1207876
UQCRHL	-0.61783	9.9144846	-6.689834	7.59E-09	2.28E-07	10.018937
RBP4	-0.617682	5.2825931	-3.841878	0.0002912	0.0014963	-0.164252
CHCHD2	-0.617598	12.315498	-11.35784	9.55E-17	1.64E-13	27.760058
B3GNT1	-0.616532	7.4590193	-5.860881	1.95E-07	3.34E-06	6.8614848
MSRA	-0.616188	5.2279821	-4.607469	2.10E-05	0.0001667	2.3405842
RNA5SP243	-0.615025	6.137183	-2.661566	0.0099071	0.0283157	-3.432399
IYD	-0.614288	5.4595672	-3.719395	0.0004339	0.0020881	-0.540412
OTTHUMG000	-0.613781	5.15735	-2.992762	0.0039746	0.0132478	-2.602106
LDHB	-0.613715	12.459138	-4.655308	1.77E-05	0.0001445	2.5048235
COX5A	-0.613407	13.442905	-6.708725	7.04E-09	2.14E-07	10.091765
CES2	-0.613377	7.5558918	-3.17299	0.0023545	0.0085949	-2.119917
CPT1A	-0.613215	8.9723893	-6.161182	6.06E-08	1.28E-06	7.9950943
SDC4	-0.613131	14.532775	-6.058043	9.07E-08	1.78E-06	7.6042006
CD274	-0.612845	6.2832841	-4.673264	1.66E-05	0.0001372	2.5666753
NDUFS8	-0.612719	7.5550662	-7.480863	3.27E-10	1.71E-08	13.085721
PPARGC1A	-0.611642	5.5174592	-4.12961	0.0001113	0.0006741	0.7477635
RANGRF	-0.611315	7.5387615	-7.493985	3.11E-10	1.65E-08	13.136774
FAM229B	-0.610352	7.5852536	-6.26336	4.07E-08	9.21E-07	8.3837797
LOC1005063	-0.609964	4.6749759	-4.046184	0.0001476	0.000853	0.4794045
OTTHUMG000	-0.609878	4.4423966	-6.45841	1.89E-08	4.90E-07	9.1292955
NIT2	-0.609518	6.4763928	-4.796824	1.06E-05	9.43E-05	2.9952666
PAM16	-0.609397	8.408211	-8.171164	2.09E-11	1.91E-09	15.770746
SMLR1	-0.609386	4.1728428	-5.749381	2.99E-07	4.76E-06	6.4443811
MT1X	-0.608683	16.36267	-3.583293	0.0006704	0.003003	-0.949357
SNORD18B	-0.608507	3.3731959	-4.443796	3.75E-05	0.0002715	1.784936
NSMCE1	-0.608101	8.9069157	-6.33058	3.12E-08	7.43E-07	8.6402109
RORC	-0.60784	5.5557954	-6.166838	5.93E-08	1.26E-06	8.0165725
FOLR1	-0.607579	7.0753421	-3.357952	0.0013507	0.0054326	-1.604284
IDH1	-0.607122	7.7423367	-3.830616	0.0003022	0.0015423	-0.199149

RNU6ATAC	-0.606077	4.7016233	-3.201742	0.0021622	0.0080277	-2.041116
ABP1	-0.605852	6.3196108	-3.24722	0.0018879	0.0071565	-1.915444
ALDH8A1	-0.604821	5.6708739	-3.945579	0.0002067	0.0011232	0.159993
APPBP2	-0.604276	10.365022	-5.041688	4.33E-06	4.43E-05	3.8589997
OTTHUMGOO(	-0.604222	6.7917613	-3.771507	0.0003665	0.0018144	-0.381291
AIP	-0.604189	6.6466595	-7.557337	2.41E-10	1.35E-08	13.38328
RBKS	-0.604051	5.9456382	-7.365419	5.18E-10	2.45E-08	12.636686
TCEAL6	-0.603661	8.0158543	-5.750951	2.97E-07	4.74E-06	6.4502361
CLIP3	-0.602243	7.463387	-5.776035	2.70E-07	4.37E-06	6.5438853
HSBP1L1	-0.602225	9.6725338	-5.757051	2.91E-07	4.65E-06	6.4730008
FTL	-0.601707	18.476959	-8.643079	3.22E-12	4.57E-10	17.598976
PIGK	-0.601586	11.530085	-4.957608	5.90E-06	5.77E-05	3.5603658
GCSH	-0.600588	8.6986779	-6.615847	1.02E-08	2.90E-07	9.733985
PHB	-0.600444	11.029528	-5.582947	5.66E-07	8.09E-06	5.8261569
DHRS4L2	-0.600418	7.5548502	-5.342564	1.41E-06	1.73E-05	4.9436331
ZER1	-0.599354	7.181328	-7.387804	4.74E-10	2.30E-08	12.723736
CMAS	-0.599156	9.8042005	-6.287704	3.70E-08	8.51E-07	8.4765827
METTL21B	-0.598129	5.6994851	-7.713057	1.30E-10	8.21E-09	13.989294
NGFRAP1	-0.596847	8.2957669	-8.424516	7.65E-12	8.79E-10	16.753487
UGT2B28	-0.596725	4.0371149	-4.6317	1.93E-05	0.000155	2.4236729
ITGB5	-0.595981	9.4444718	-4.361733	5.00E-05	0.000344	1.510164
GABARAPL2	-0.595955	12.801595	-5.968748	1.28E-07	2.36E-06	7.2670503
TRAPPC6A	-0.595935	7.0326118	-6.048427	9.41E-08	1.84E-06	7.5678354
SLC4A1	-0.595514	4.9625326	-3.358117	0.0013501	0.0054307	-1.603816
NUAK2	-0.595262	5.7805621	-5.492645	7.99E-07	1.08E-05	5.4931114
FAXDC2	-0.595178	7.4689938	-6.264512	4.05E-08	9.19E-07	8.3881723
LOC100506(	-0.594967	5.1324908	-4.130018	0.0001111	0.0006735	0.7490833
SLC2A3	-0.594797	7.4360359	-3.40608	0.0011655	0.0047918	-1.466811
RUSC2	-0.594558	7.3621743	-5.221003	2.22E-06	2.53E-05	4.5025526
NICN1-AS1	-0.594488	5.3274536	-6.45482	1.92E-08	4.95E-07	9.115538
AKR7L	-0.594411	5.7461403	-6.723547	6.64E-09	2.03E-07	10.148927
FKBP4	-0.594361	8.6230943	-7.165833	1.15E-09	4.81E-08	11.86114
PLEKHA5	-0.593961	9.5751734	-6.996119	2.25E-09	8.21E-08	11.202839
FRAT1	-0.593244	5.854909	-6.915386	3.10E-09	1.07E-07	10.890189
PRDX3	-0.593184	12.342952	-5.46674	8.81E-07	1.17E-05	5.3978987
CDC14A	-0.592934	8.9262544	-3.322182	0.0015061	0.0059407	-1.705589
LOC143666	-0.592501	4.5829979	-6.328246	3.15E-08	7.49E-07	8.6312972
OTTHUMGOO(	-0.592483	11.004684	-3.096407	0.0029476	0.0103672	-2.327316
MAGI2-AS3	-0.592317	13.1992	-3.032464	0.0035471	0.0120345	-2.497657
RNU12	-0.592205	3.5016061	-5.960732	1.32E-07	2.42E-06	7.2368465
GABARAP	-0.591589	14.687715	-11.11811	2.33E-16	3.00E-13	26.895441
NUPR1	-0.591524	8.7225356	-4.745489	1.28E-05	0.0001103	2.816583
SUSD2	-0.590901	5.8406749	-4.482435	3.27E-05	0.0002424	1.9152137
BLOC1S1	-0.590773	4.7712528	-9.228961	3.21E-13	7.46E-11	19.850918
CISD3	-0.589786	7.7075854	-5.614301	5.02E-07	7.31E-06	5.9421969
WWC2	-0.58954	9.5565	-4.932313	6.48E-06	6.22E-05	3.4709325
SERINC5	-0.588921	8.8225331	-4.143942	0.000106	0.0006461	0.7941735
BLVRA	-0.58858	9.2607338	-7.287425	7.07E-10	3.19E-08	12.333479
IMP3	-0.588393	5.6395597	-9.133384	4.67E-13	9.88E-11	19.485228
CCDC11	-0.587878	4.9706621	-6.021585	1.04E-07	2.00E-06	7.4663978
PCNA-AS1	-0.587844	6.0342267	-4.808447	1.02E-05	9.14E-05	3.035844

LINC00864	-0.587487	5.1397405	-4.910549	7.02E-06	6.67E-05	3.3941374
NAA60	-0.587245	8.2940493	-9.259588	2.85E-13	6.71E-11	19.967946
PTOV1	-0.587051	8.3700518	-10.21649	7.00E-15	4.26E-12	23.581163
NPAS3	-0.586991	7.2206436	-3.718793	0.0004347	0.0020911	-0.542242
FTCD	-0.586612	5.2052113	-4.420483	4.07E-05	0.0002904	1.7066111
TACR1	-0.585959	5.5625913	-4.893354	7.47E-06	7.03E-05	3.333569
GAS1	-0.58591	7.6399149	-5.467114	8.80E-07	1.17E-05	5.3992711
TJP1	-0.585605	14.705416	-4.165609	9.84E-05	0.0006075	0.86451
GRAMD1B	-0.585525	4.7686507	-5.46491	8.87E-07	1.18E-05	5.391176
ELP4	-0.585076	8.3081746	-8.402672	8.35E-12	9.31E-10	16.668857
SNORD94	-0.585064	7.3379762	-2.55557	0.0130853	0.0355768	-3.681974
EIF3M	-0.58474	14.355698	-4.958467	5.89E-06	5.76E-05	3.5634062
CRYZ	-0.584462	9.8803728	-3.804938	0.0003287	0.0016559	-0.27849
PFKFB2	-0.584318	6.6525559	-5.007363	4.92E-06	4.94E-05	3.7368362
KCNMB2-IT1	-0.583886	4.9431021	-3.779476	0.0003571	0.0017747	-0.356838
KYNU	-0.582801	5.3057008	-4.567032	2.43E-05	0.0001878	2.2023908
SLC41A1	-0.582055	6.6695164	-7.977264	4.53E-11	3.45E-09	15.017202
ARHGAP5	-0.581838	10.111889	-7.450734	3.69E-10	1.90E-08	12.968506
NDUFC1	-0.581377	7.2480339	-10.88371	5.59E-16	6.64E-13	26.043094
OTTHUMG000	-0.581237	4.8864633	-4.2739	6.79E-05	0.000444	1.219044
DENND5B-AS	-0.581222	4.7766898	-6.521852	1.47E-08	3.96E-07	9.3726787
SLC43A2	-0.580936	6.2051926	-5.737616	3.13E-07	4.94E-06	6.4005002
SNORA26	-0.580604	5.4592716	-3.727621	0.0004225	0.0020422	-0.515387
EZR	-0.580075	12.264269	-6.614226	1.02E-08	2.92E-07	9.7277449
SIAE	-0.580047	8.0403341	-6.38991	2.48E-08	6.12E-07	8.8669852
NDNF	-0.579987	15.769148	-4.413785	4.17E-05	0.0002958	1.684148
DHDH	-0.579458	4.865982	-8.982328	8.45E-13	1.52E-10	18.90585
ITPRIP	-0.579433	5.3145779	-4.636283	1.89E-05	0.0001529	2.4394091
ABHD4	-0.579364	7.6692851	-5.970944	1.27E-07	2.35E-06	7.2753258
HAUS4	-0.579124	7.3200538	-8.854812	1.40E-12	2.34E-10	18.415493
HMGN2P25	-0.579119	9.5630603	-5.681444	3.88E-07	5.92E-06	6.1913653
MTSS1L	-0.578681	10.665566	-3.3018	0.001602	0.0062501	-1.76298
UACA	-0.57811	15.593625	-3.853728	0.0002801	0.0014478	-0.127461
FGFBP2	-0.578	3.607218	-5.52746	7.00E-07	9.67E-06	5.6213041
TENM2	-0.577945	7.2739908	-2.995128	0.0039478	0.0131785	-2.595909
MEGF9	-0.57776	9.0721408	-4.547116	2.60E-05	0.0001987	2.1345451
DNAJC3-AS1	-0.577736	5.3639643	-5.643957	4.48E-07	6.65E-06	6.0521412
SGK2	-0.57766	4.9553539	-5.784841	2.61E-07	4.25E-06	6.5767879
CALML3-AS1	-0.577439	3.9013425	-5.366322	1.29E-06	1.61E-05	5.030265
WWP1	-0.577378	10.301796	-4.794632	1.07E-05	9.50E-05	2.9876221
PER1	-0.576667	5.8524484	-4.752077	1.25E-05	0.000108	2.8394659
EEF1DP1	-0.576251	6.6311539	-5.090778	3.61E-06	3.80E-05	4.034302
C15orf27	-0.576144	5.3611689	-5.280165	1.78E-06	2.11E-05	4.7167569
SRFBP1	-0.576129	10.696779	-4.101911	0.0001223	0.0007299	0.6583201
RAMP2	-0.5761	10.924615	-4.153536	0.0001026	0.0006284	0.825291
OTTHUMG000	-0.575922	4.3769941	-3.924064	0.000222	0.0011878	0.0922979
WBSCR16	-0.575913	6.7674731	-5.06103	4.03E-06	4.18E-05	3.9279922
SLC3A2	-0.575829	7.5315736	-6.656936	8.64E-09	2.53E-07	9.8921747
CERS6-AS1	-0.575411	10.713963	-3.832749	0.0003001	0.0015334	-0.192546
HNF4A	-0.574904	6.0941562	-4.642065	1.86E-05	0.0001504	2.4592785
MRPL11	-0.574705	6.4528579	-8.661818	2.99E-12	4.32E-10	17.671349

C12orf10	-0.574248	7.1715854	-9.636113	6.56E-14	2.05E-11	21.400136
MRPL43	-0.574087	7.1133087	-7.651361	1.66E-10	9.92E-09	13.749188
HECW2	-0.574073	9.6692843	-2.730611	0.0082339	0.0242488	-3.265517
PNRC1	-0.57392	11.565475	-9.475239	1.23E-13	3.38E-11	20.789747
COQ5	-0.573774	9.05603	-5.077318	3.80E-06	3.97E-05	3.9861699
PCMTD1	-0.573728	9.3011616	-4.802151	1.04E-05	9.30E-05	3.0138589
GGTLC3	-0.571875	6.1502472	-2.573309	0.0124961	0.0342703	-3.640773
EIF3K	-0.57185	14.165577	-7.22015	9.24E-10	4.02E-08	12.072079
IL17RD	-0.571746	7.6376652	-4.634847	1.90E-05	0.0001535	2.43448
SMPD1	-0.571634	8.4816075	-4.844308	8.94E-06	8.21E-05	3.1613125
LOC1002937	-0.571624	5.132093	-4.074886	0.000134	0.0007875	0.5713792
RAB1B	-0.571479	10.827131	-7.920951	5.67E-11	4.18E-09	14.798194
PIH1D1	-0.571348	7.3445925	-6.703107	7.20E-09	2.17E-07	10.070101
LOC148709	-0.57129	4.8209354	-6.011997	1.08E-07	2.05E-06	7.4301925
FAM98A	-0.571241	8.4219589	-4.947021	6.14E-06	5.95E-05	3.5229106
CALML3	-0.571193	4.2090249	-5.619599	4.92E-07	7.18E-06	5.9618263
TMED1	-0.570867	4.7599523	-8.734941	2.24E-12	3.46E-10	17.953562
SLC25A38	-0.570717	9.345463	-6.417228	2.22E-08	5.57E-07	8.9715333
CRIM1	-0.570599	14.524848	-3.08232	0.0030709	0.0107192	-2.365068
SNORD116-3	-0.570464	5.9770221	-3.087059	0.0030289	0.0106049	-2.352383
SNORD116-9	-0.570464	5.9770221	-3.087059	0.0030289	0.0106049	-2.352383
TCEAL2	-0.57038	9.494361	-4.558447	2.50E-05	0.0001928	2.1731275
OTTHUMG000	-0.570337	4.9607277	-4.746335	1.28E-05	0.00011	2.8195201
GSS	-0.570276	7.3648115	-5.968588	1.28E-07	2.37E-06	7.2664487
DNAJC6	-0.569794	6.2048518	-5.15786	2.81E-06	3.09E-05	4.2749373
OTTHUMG000	-0.569742	5.8435449	-5.196865	2.43E-06	2.74E-05	4.4154176
TP53TG1	-0.569651	8.7635097	-5.045597	4.27E-06	4.38E-05	3.8729369
ARF1	-0.569472	13.37019	-8.684342	2.74E-12	4.01E-10	17.758311
CLIC5	-0.568805	12.727474	-3.394681	0.0012071	0.0049364	-1.499491
SULT1A2	-0.568362	6.5318238	-5.962495	1.31E-07	2.40E-06	7.2434877
FCN1	-0.568199	6.3211772	-2.65709	0.0100255	0.0285856	-3.4431
CDADC1	-0.56798	7.6849277	-6.83966	4.19E-09	1.38E-07	10.597288
HILPDA	-0.567757	5.8429943	-6.825636	4.43E-09	1.46E-07	10.543084
SKAP1	-0.567209	6.0585489	-4.897796	7.35E-06	6.95E-05	3.3492074
MRPL34	-0.567043	3.8942056	-7.980376	4.47E-11	3.42E-09	15.029303
ECI1	-0.567006	6.2291118	-8.096546	2.82E-11	2.40E-09	15.48088
OTTHUMG000	-0.566981	6.2045605	-3.66456	0.0005175	0.0024174	-0.706343
GTF2A2	-0.566968	8.6672974	-4.771862	1.16E-05	0.0001016	2.9082714
ZFP36L2	-0.566856	9.8256048	-6.116448	7.22E-08	1.47E-06	7.8253668
AUH	-0.566853	7.8818077	-5.383192	1.21E-06	1.52E-05	5.0918606
ADH5	-0.566715	9.4600352	-6.932408	2.90E-09	1.01E-07	10.95608
AIG1	-0.566352	6.7094434	-5.204644	2.36E-06	2.67E-05	4.4434799
RBPMS2	-0.565792	6.843039	-5.8295	2.20E-07	3.69E-06	6.7438677
SVIP	-0.565663	10.35397	-7.675176	1.51E-10	9.16E-09	13.84187
OTTHUMG000	-0.565499	4.841849	-3.109103	0.0028405	0.0100591	-2.293188
ZNF277	-0.564991	9.7229097	-6.525133	1.45E-08	3.92E-07	9.3852768
RNF186	-0.564676	4.3316292	-4.690799	1.56E-05	0.0001303	2.6271873
C21orf119	-0.564432	5.4423884	-5.183912	2.55E-06	2.85E-05	4.3687208
ZNF844	-0.564368	8.0992141	-4.224001	8.06E-05	0.0005127	1.055062
LINC00844	-0.563819	4.1063231	-2.805725	0.0067108	0.0204878	-3.080188
AASS	-0.563191	8.3709441	-4.7975	1.06E-05	9.42E-05	2.997626

CYP2B7P1	-0.56305	4.5226	-5.656307	4.28E-07	6.39E-06	6.0979774
ETHE1	-0.562867	8.6259569	-4.306251	6.07E-05	0.0004041	1.3259064
IGBP1	-0.562618	10.274827	-8.112041	2.65E-11	2.29E-09	15.541088
UQCR11	-0.562347	12.178664	-5.770125	2.76E-07	4.46E-06	6.5218107
OTTHUMG000	-0.562144	5.5118498	-3.279516	0.0017134	0.006615	-1.825442
MAN2A2	-0.561838	7.1519366	-6.242788	4.41E-08	9.89E-07	8.305417
C2orf74	-0.56177	8.7133472	-3.438939	0.0010531	0.004395	-1.372188
SLC25A42	-0.561621	5.1165754	-5.902646	1.66E-07	2.93E-06	7.0182862
VPS4A	-0.561611	7.7416056	-5.707454	3.52E-07	5.45E-06	6.2881287
VPS25	-0.561433	9.1119621	-3.95062	0.0002033	0.0011082	0.1758861
MRPL53	-0.561136	7.3219225	-7.275076	7.43E-10	3.32E-08	12.285486
C10orf54	-0.561049	5.7317589	-8.388912	8.81E-12	9.63E-10	16.615538
ARMCX3	-0.560794	9.0833287	-5.960206	1.33E-07	2.42E-06	7.2348629
PARD3B	-0.56063	9.5690356	-3.34897	0.0013883	0.005558	-1.629793
MPLKIP	-0.560282	7.3427089	-6.656429	8.66E-09	2.53E-07	9.8902209
HLA-E	-0.55938	13.133723	-5.643839	4.49E-07	6.65E-06	6.0517011
SULT1A1	-0.559328	6.0278079	-5.604077	5.22E-07	7.54E-06	5.9043362
SLC6A2	-0.559204	4.0541728	-5.171378	2.68E-06	2.97E-05	4.3235776
TMEM259	-0.559157	10.817988	-4.887088	7.65E-06	7.18E-05	3.3115213
DNAJC4	-0.558097	7.5459218	-7.722614	1.25E-10	7.97E-09	14.026486
GCHFR	-0.55776	5.4195866	-7.131957	1.31E-09	5.35E-08	11.729637
OLFML2A	-0.557546	8.2257221	-4.006315	0.0001688	0.0009525	0.3522655
CGNL1	-0.557313	13.299138	-3.363855	0.0013266	0.0053537	-1.587496
GSTA4	-0.557	8.5417008	-5.618955	4.93E-07	7.19E-06	5.9594406
DAZAP2	-0.556358	13.76333	-8.053554	3.34E-11	2.71E-09	15.313798
APRT	-0.5552	10.914698	-4.930968	6.51E-06	6.25E-05	3.4661836
OTTHUMG000	-0.554507	3.5382793	-2.506918	0.0148327	0.039426	-3.793794
RNF38	-0.554286	10.553302	-10.11772	1.02E-14	5.75E-12	23.212468
KREMEN1	-0.554221	6.777378	-5.27562	1.81E-06	2.14E-05	4.7002683
OTTHUMG000	-0.55345	4.0170654	-4.461396	3.53E-05	0.000258	1.844206
ABHD14B	-0.553174	6.4791789	-10.2157	7.03E-15	4.26E-12	23.578234
TMEM38B	-0.553036	6.6053261	-4.888574	7.60E-06	7.14E-05	3.3167473
EIF1AX	-0.552975	12.699823	-4.398843	4.39E-05	0.0003084	1.6340942
OTTHUMG000	-0.552812	7.1383393	-4.964622	5.75E-06	5.65E-05	3.5851983
SLC51B	-0.55278	5.7027836	-4.08914	0.0001277	0.0007562	0.6171959
FIS1	-0.551748	9.8660818	-9.859117	2.77E-14	1.06E-11	22.242212
SOD1	-0.551626	14.245805	-7.460581	3.55E-10	1.84E-08	13.006815
LIN7A	-0.551495	6.2054726	-4.064562	0.0001387	0.0008107	0.5382529
PLA2G15	-0.551412	6.3703118	-6.731848	6.42E-09	1.98E-07	10.180944
ASMTL	-0.551367	5.3774123	-8.892503	1.20E-12	2.08E-10	18.560547
RHOB	-0.551109	9.3582316	-5.384087	1.20E-06	1.52E-05	5.0951306
MAN2B2	-0.550952	7.1379336	-7.782935	9.82E-11	6.60E-09	14.261228
RAB11FIP3	-0.55093	6.4664741	-5.383682	1.21E-06	1.52E-05	5.0936516
ATP5EP2	-0.55092	10.86992	-6.743312	6.14E-09	1.91E-07	10.225176
HIST1H2BF	-0.550262	5.2196141	-3.713188	0.0004427	0.0021217	-0.559272
MARC2	-0.549983	8.0259128	-3.374107	0.0012857	0.0052096	-1.558289
C1orf115	-0.549791	3.3998785	-7.646001	1.69E-10	1.01E-08	13.728328
SLC10A2	-0.549316	4.755011	-2.516165	0.0144852	0.0386619	-3.772676
MRPS21	-0.547792	9.9158089	-5.728441	3.24E-07	5.10E-06	6.3663009
OXT	-0.547571	5.5972815	-5.000777	5.04E-06	5.04E-05	3.7134358
CHMP4C	-0.54749	9.6515438	-4.306447	6.06E-05	0.0004041	1.3265533

IL1B	-0.547279	4.5117703	-4.807495	1.02E-05	9.16E-05	3.0325182
SLC9A3	-0.547032	5.4302708	-3.975308	0.0001872	0.0010368	0.2538909
GOLT1A	-0.546939	4.3215238	-5.539289	6.69E-07	9.31E-06	5.6649209
MTRNR2L9	-0.546812	8.7447936	-8.866954	1.33E-12	2.25E-10	18.462232
SNORD116-2	-0.546164	7.0457505	-3.158425	0.0024579	0.0089092	-2.159643
GPHN	-0.546	8.9959477	-3.701386	0.0004598	0.0021917	-0.595081
CDH13	-0.545919	8.3510475	-2.924334	0.0048255	0.0155735	-2.779714
ATXN7L3B	-0.545627	10.293007	-6.879434	3.58E-09	1.22E-07	10.751084
TXNL4A	-0.545604	6.4755448	-8.031839	3.64E-11	2.91E-09	15.229389
CHDH	-0.545371	4.6144446	-5.89846	1.68E-07	2.98E-06	7.0025586
EYA2	-0.545344	5.5465741	-7.473106	3.37E-10	1.76E-08	13.05554
DHPS	-0.545301	8.3099995	-6.220306	4.81E-08	1.06E-06	8.2198363
JUND	-0.544846	4.8951628	-7.448665	3.72E-10	1.91E-08	12.960459
NRIP2	-0.544661	10.148567	-3.158827	0.0024549	0.0088997	-2.158548
CENPQ	-0.544488	6.2549487	-6.546966	1.33E-08	3.63E-07	9.4691316
EAF2	-0.544403	6.0343377	-4.28332	6.57E-05	0.000432	1.2501139
PSMB3	-0.544261	13.615575	-5.011918	4.83E-06	4.88E-05	3.7530283
KCND3	-0.544022	7.9373915	-2.710735	0.0086869	0.0253343	-3.313902
CLUH	-0.543594	5.7361003	-6.270995	3.95E-08	8.98E-07	8.4128797
ZDHHC12	-0.543427	6.0106533	-7.486362	3.20E-10	1.68E-08	13.107112
MTCH2	-0.54334	12.188339	-4.915966	6.88E-06	6.55E-05	3.4132396
OTTHUMG000	-0.543063	11.45979	-3.327299	0.0014829	0.0058716	-1.691143
NFE2L1	-0.542742	10.918581	-5.212982	2.29E-06	2.59E-05	4.4735807
TSPAN33	-0.542711	8.3552157	-3.696277	0.0004675	0.002223	-0.610557
LOC1005073	-0.542193	6.9371974	-2.505559	0.0148844	0.039543	-3.796893
LMX1B	-0.54164	7.1313659	-4.93758	6.36E-06	6.12E-05	3.4895392
HMGCL	-0.541615	5.1526605	-9.300722	2.42E-13	5.85E-11	20.125003
OR2T11	-0.541433	4.0345872	-5.151431	2.88E-06	3.15E-05	4.2518227
OTTHUMG000	-0.541312	8.205913	-3.306278	0.0015804	0.0061827	-1.750391
MMGT1	-0.540937	9.148878	-7.33255	5.91E-10	2.73E-08	12.508889
CHRDL1	-0.540924	9.2764082	-2.912306	0.0049915	0.0160238	-2.810613
HOXC-AS1	-0.54033	5.8325292	-4.026477	0.0001577	0.0009002	0.4164716
FZD2	-0.540304	8.0194046	-6.127908	6.91E-08	1.42E-06	7.8688202
PDZD11	-0.540125	8.7515203	-8.036228	3.58E-11	2.87E-09	15.246451
SH3YL1	-0.540041	5.968332	-5.077725	3.79E-06	3.96E-05	3.9876244
CRAT	-0.539778	6.7070959	-4.993874	5.17E-06	5.14E-05	3.6889197
FUNDC1	-0.539704	7.3537885	-7.416262	4.23E-10	2.11E-08	12.834418
CCNG1	-0.53957	10.345328	-5.651873	4.35E-07	6.49E-06	6.0815158
ECI2	-0.539128	7.4933382	-4.600492	2.15E-05	0.0001701	2.3166961
RGS10	-0.539081	9.4453669	-5.757266	2.90E-07	4.64E-06	6.473801
SHISA4	-0.538575	6.9971272	-5.564414	6.08E-07	8.58E-06	5.7576608
LANCL1	-0.538478	8.1888639	-5.331757	1.47E-06	1.79E-05	4.9042718
LOC1005076	-0.538089	5.1444664	-5.549962	6.42E-07	8.99E-06	5.7043002
ACOT4	-0.537688	4.2523215	-5.739748	3.11E-07	4.91E-06	6.4084497
ACSF2	-0.537088	6.2962731	-3.570825	0.0006973	0.003103	-0.986328
GIMAP2	-0.53653	6.8670105	-3.820145	0.0003127	0.0015875	-0.231543
COASY	-0.536387	5.9432569	-10.47492	2.62E-15	2.02E-12	24.540825
ST13P19	-0.536192	4.0502277	-7.402037	4.48E-10	2.20E-08	12.779092
DCAF11	-0.534887	7.190599	-6.99617	2.25E-09	8.21E-08	11.203037
EMCN	-0.53486	14.830974	-3.562372	0.0007162	0.0031696	-1.011346
FER1L6	-0.534556	3.9801346	-4.564792	2.45E-05	0.000189	2.1947531

MRPL54	-0.534065	10.432212	-4.402255	4.34E-05	0.0003055	1.6455159
HINT3	-0.534015	7.4535059	-6.899705	3.30E-09	1.13E-07	10.829509
DCN	-0.533997	15.573064	-4.589818	2.24E-05	0.0001753	2.2801897
CNBP	-0.533968	13.865498	-10.03123	1.43E-14	7.22E-12	22.888722
CDK2AP2	-0.53381	5.3036562	-6.246947	4.34E-08	9.74E-07	8.3212557
TM7SF3	-0.533735	10.252854	-3.317726	0.0015266	0.006009	-1.718158
BBS12	-0.533534	5.7507277	-7.038215	1.91E-09	7.19E-08	11.365997
RBM11	-0.532678	5.7002213	-4.363261	4.97E-05	0.0003425	1.5152568
ZNF204P	-0.532509	6.3581926	-5.605697	5.19E-07	7.50E-06	5.9103352
SNX12	-0.532465	8.0184644	-8.11729	2.59E-11	2.26E-09	15.56148
CIB1	-0.532389	8.9374539	-5.62337	4.85E-07	7.10E-06	5.9757983
SIRPAP1	-0.532257	5.1664718	-6.763421	5.67E-09	1.78E-07	10.302786
HYKK	-0.531698	4.9479772	-5.797347	2.49E-07	4.09E-06	6.6235383
PLA2G12B	-0.531551	5.0115211	-2.984584	0.0040684	0.0135145	-2.623493
NDUFV3	-0.53131	8.2654621	-6.315832	3.31E-08	7.78E-07	8.5839043
LPCAT3	-0.531281	11.16368	-4.407671	4.26E-05	0.000301	1.6636568
FGFR4	-0.531143	6.085527	-9.249201	2.96E-13	6.94E-11	19.928263
CPT2	-0.531049	6.4616333	-5.608883	5.13E-07	7.44E-06	5.9221305
ADAMTS9-AS	-0.530686	11.79148	-2.398683	0.0194969	0.0493853	-4.036257
ACTN4	-0.530518	15.194272	-3.868733	0.0002666	0.0013871	-0.080781
ICOSLG	-0.53046	4.8746893	-7.335752	5.83E-10	2.71E-08	12.521336
MTHFD2	-0.530149	7.3609849	-5.108127	3.39E-06	3.61E-05	4.0964165
FZD8	-0.529703	5.324091	-4.015241	0.0001638	0.000929	0.3806677
WBP2	-0.529675	9.7947539	-6.082142	8.25E-08	1.65E-06	7.6953963
PIGH	-0.52964	7.7817169	-5.917431	1.56E-07	2.79E-06	7.0738634
SIK3	-0.529298	7.9541236	-6.115357	7.25E-08	1.48E-06	7.8212302
TUFM	-0.528916	8.6478089	-6.213929	4.93E-08	1.08E-06	8.1955728
PTGR1	-0.528696	8.8483418	-2.625744	0.0108924	0.0305582	-3.517648
MIF	-0.528555	12.124482	-4.969216	5.66E-06	5.57E-05	3.6014688
MMP28	-0.528365	5.5945716	-6.031046	1.01E-07	1.95E-06	7.5021385
UPF1	-0.528009	6.6748169	-8.062654	3.22E-11	2.66E-09	15.349168
FAM168A	-0.527862	8.8541477	-5.868196	1.89E-07	3.27E-06	6.8889262
SERPINF2	-0.527122	6.2405821	-2.759434	0.0076154	0.0227286	-3.194864
LOC1002878	-0.527119	5.8159887	-3.180622	0.0023019	0.008442	-2.099049
LOC1006533	-0.526976	6.6593928	-4.012552	0.0001653	0.0009357	0.3721067
HSPB8	-0.526888	6.2531249	-6.546401	1.34E-08	3.64E-07	9.4669616
TSR2	-0.526468	10.264226	-5.796215	2.50E-07	4.10E-06	6.6193052
SLC26A4	-0.526349	4.8912802	-2.760389	0.0075957	0.0226872	-3.192514
PRPF6	-0.526253	7.3566156	-8.363243	9.76E-12	1.04E-09	16.516045
ALAD	-0.525815	6.0446341	-5.995556	1.16E-07	2.17E-06	7.3681389
METTL1	-0.525473	5.1787233	-5.978735	1.23E-07	2.30E-06	7.304696
OCIAD2	-0.525469	10.419105	-3.038471	0.0034863	0.0118595	-2.481768
SF3B5	-0.525148	8.940843	-8.507328	5.51E-12	6.81E-10	17.074119
CHCHD2P8	-0.525012	7.686482	-9.593065	7.75E-14	2.40E-11	21.237034
VDAC3	-0.524837	7.4036772	-8.646161	3.18E-12	4.53E-10	17.610881
OTTHUMG000	-0.524554	11.021911	-2.627988	0.0108281	0.0304138	-3.512334
ACOT1	-0.524436	6.5719366	-7.163907	1.16E-09	4.83E-08	11.853663
PLIN2	-0.524329	8.8624098	-2.673886	0.0095874	0.0275371	-3.402867
GRPEL1	-0.523722	7.892972	-6.256397	4.18E-08	9.41E-07	8.3572509
KGFLP1	-0.522783	5.4644082	-3.929618	0.000218	0.001171	0.1097516
TSPAN7	-0.522318	11.119434	-2.882194	0.0054305	0.0172093	-2.887551

CYP39A1	-0.522189	4.7556366	-5.29262	1.70E-06	2.03E-05	4.7619629
ROBO2	-0.521933	7.1944198	-2.635478	0.0106162	0.0299283	-3.494574
DCAKD	-0.521843	6.684643	-9.802925	3.44E-14	1.24E-11	22.030485
FAM174B	-0.521839	5.3973461	-8.495385	5.78E-12	7.06E-10	17.027898
MT1P2	-0.521336	4.3749484	-5.311578	1.58E-06	1.91E-05	4.8308497
PAPSS1	-0.520932	8.4077172	-7.43951	3.86E-10	1.98E-08	12.924845
PRKG1	-0.520778	7.2780592	-4.366056	4.93E-05	0.00034	1.5245728
PNMA2	-0.520623	7.9320723	-5.557266	6.24E-07	8.78E-06	5.7312613
SH3GL2	-0.5206	4.6697984	-5.511359	7.44E-07	1.01E-05	5.5619833
ENPP3	-0.520523	5.0109552	-3.150459	0.0025162	0.0090823	-2.181313
CDS1	-0.52018	7.5622905	-3.95838	0.0001981	0.0010876	0.2003739
HDHD3	-0.52008	5.5631862	-6.189355	5.43E-08	1.17E-06	8.1021248
PRR13	-0.519803	11.526149	-8.561204	4.45E-12	5.83E-10	17.282539
GADD45G	-0.519491	4.2802323	-4.453081	3.63E-05	0.0002644	1.8161887
SH3BGR	-0.519433	6.6715611	-4.946639	6.15E-06	5.95E-05	3.5215604
OTTHUMG000	-0.519296	6.7183598	-5.077314	3.80E-06	3.97E-05	3.986156
IL1R2	-0.519285	3.655397	-6.209902	5.01E-08	1.09E-06	8.1802552
METTL7A	-0.519063	8.55261	-3.563926	0.0007127	0.0031572	-1.00675
HADHB	-0.519011	9.7361198	-6.556133	1.29E-08	3.53E-07	9.5043532
PLAT	-0.518678	10.81763	-2.916377	0.0049347	0.0158696	-2.800166
SHISA2	-0.518654	6.6202041	-2.812054	0.0065951	0.0201925	-3.064396
MRPL41	-0.518504	10.038737	-7.223511	9.12E-10	3.98E-08	12.085134
OTTHUMG000	-0.518412	5.6288954	-2.710516	0.008692	0.0253444	-3.314432
LOH12CR2	-0.518161	3.7116974	-5.881407	1.80E-07	3.14E-06	6.9385116
MRPL12	-0.517807	6.3773023	-5.87647	1.83E-07	3.19E-06	6.9199794
TMEM204	-0.517689	7.8198436	-4.730785	1.35E-05	0.0001155	2.7655617
DNAJC15	-0.516957	12.997287	-4.500251	3.07E-05	0.0002298	1.9754711
FOXD2	-0.516854	4.3628166	-5.386272	1.19E-06	1.51E-05	5.1031161
PAMR1	-0.516805	5.3590825	-2.914975	0.0049542	0.0159224	-2.803767
NFATC3	-0.516623	7.5221644	-11.967	1.03E-17	2.44E-14	29.923732
ATP6V1B1	-0.516284	5.8399598	-2.889676	0.0053182	0.0169129	-2.86849
MIR1182	-0.516021	7.8268208	-3.104124	0.0028821	0.0101729	-2.306584
COX6A1P2	-0.51595	15.247074	-5.951974	1.37E-07	2.49E-06	7.2038566
HBD	-0.5158	3.5076282	-7.352284	5.46E-10	2.57E-08	12.585612
HIST1H2BC	-0.515571	5.7809498	-5.245821	2.03E-06	2.34E-05	4.5923002
TTC32	-0.515067	7.9788816	-3.629369	0.0005791	0.0026531	-0.812005
NKAPL	-0.514792	3.7352589	-4.338528	5.42E-05	0.0003681	1.4329475
FCGR3A	-0.514146	7.5216348	-2.762223	0.0075579	0.0226044	-3.187997
SPAG7	-0.512878	8.2953107	-6.219146	4.84E-08	1.06E-06	8.215422
TOX3	-0.512799	5.8906898	-3.11983	0.0027528	0.009807	-2.264273
GJA3	-0.51272	5.8408292	-3.765054	0.0003743	0.0018453	-0.401069
PHACTR4	-0.512482	12.735005	-3.910019	0.0002326	0.0012354	0.0482254
NDUFA11	-0.512445	9.4071898	-8.361134	9.84E-12	1.04E-09	16.50787
NPR1	-0.512378	10.183828	-4.387678	4.57E-05	0.0003183	1.5967534
KMO	-0.512234	5.7555418	-3.054203	0.0033316	0.0114504	-2.440039
AADACL4	-0.512184	3.5304385	-3.489893	0.000899	0.0038362	-1.224253
CLPTM1	-0.511473	11.775333	-6.713041	6.92E-09	2.11E-07	10.108407
NDUFB10	-0.511333	12.854572	-6.251868	4.25E-08	9.57E-07	8.3399981
TAF7	-0.511311	10.240712	-7.349635	5.52E-10	2.59E-08	12.575314
CCDC6	-0.511159	11.132154	-6.512993	1.52E-08	4.08E-07	9.3386675
HIST2H2AC	-0.511128	15.21771	-7.07805	1.63E-09	6.34E-08	11.520474

EPHX1	-0.511101	9.4647679	-6.111266	7.37E-08	1.50E-06	7.805722
LOC284080	-0.510981	5.4676923	-4.254832	7.25E-05	0.0004687	1.1562579
EPB41L3	-0.510911	6.4001098	-3.976175	0.0001867	0.0010345	0.2566376
ETFA	-0.510724	11.328792	-4.606973	2.10E-05	0.0001669	2.338885
HNF4G	-0.510425	5.6425697	-3.054025	0.0033333	0.0114551	-2.440512
COPZ1	-0.510284	8.7919134	-8.228697	1.67E-11	1.56E-09	15.994128
OSTF1	-0.510172	10.777733	-4.691811	1.55E-05	0.00013	2.6306808
TACC2	-0.51002	7.9053782	-4.752309	1.25E-05	0.000108	2.8402702
PSMF1	-0.509933	8.6464225	-8.42821	7.54E-12	8.70E-10	16.767798
CNN2	-0.509812	9.9965428	-5.433253	1.00E-06	1.30E-05	5.275041
OTTHUMG00(	-0.509729	5.327291	-5.856244	1.98E-07	3.38E-06	6.8440951
RNA5S17	-0.509648	6.075828	-4.6599	1.74E-05	0.0001425	2.52063
CNNM2	-0.509409	7.0801133	-5.147859	2.92E-06	3.19E-05	4.2389862
UPB1	-0.509274	4.8643584	-3.795524	0.0003389	0.0016995	-0.307493
PTPLAD2	-0.509156	6.5842656	-4.876269	7.95E-06	7.41E-05	3.2734803
OTTHUMG00(	-0.509113	6.351302	-3.658437	0.0005278	0.0024565	-0.724774
CHP1	-0.509103	13.062551	-9.126215	4.80E-13	1.00E-10	19.457767
ZCRB1	-0.50897	11.159875	-7.800603	9.15E-11	6.26E-09	14.329978
KCNJ3	-0.508905	4.8282003	-4.935358	6.41E-06	6.17E-05	3.4816879
UBL7	-0.508328	8.4420497	-5.709368	3.49E-07	5.42E-06	6.2952531
KLF15	-0.508218	5.0892651	-8.433098	7.40E-12	8.59E-10	16.786729
CIRBP	-0.507488	13.098011	-7.422069	4.14E-10	2.08E-08	12.857003
OTTHUMG00(	-0.507378	5.7634472	-3.291093	0.0016546	0.0064279	-1.793027
POLR2I	-0.507273	10.375781	-5.944961	1.41E-07	2.55E-06	7.177451
C10orf114	-0.50727	4.6767374	-6.219525	4.83E-08	1.06E-06	8.2168626
MRPS12	-0.507001	4.3685461	-7.927929	5.51E-11	4.09E-09	14.825335
ASB13	-0.506974	5.376852	-5.641049	4.53E-07	6.71E-06	6.0413497
NUCB1	-0.506953	8.1931559	-5.428151	1.02E-06	1.32E-05	5.2563461
NCBP2-AS2	-0.506663	6.1998118	-4.900594	7.28E-06	6.88E-05	3.3590606
KLF7	-0.506596	10.072865	-4.142794	0.0001064	0.0006482	0.7904543
GNG5P2	-0.505884	6.6753064	-7.092029	1.54E-09	6.06E-08	11.574704
PTPN13	-0.505757	11.26428	-4.056397	0.0001426	0.0008291	0.5120891
SBDS	-0.505699	13.012644	-5.812241	2.35E-07	3.90E-06	6.679257
HAAO	-0.505619	5.3866354	-6.18065	5.62E-08	1.20E-06	8.069044
C5orf22	-0.505307	7.8344559	-5.976481	1.24E-07	2.31E-06	7.2961992
DYNLRB1	-0.505266	10.61768	-4.440099	3.80E-05	0.0002745	1.7725025
CETN2	-0.505247	11.424352	-7.652014	1.65E-10	9.92E-09	13.751727
SNORD116-1	-0.504964	6.3512257	-2.511897	0.0146447	0.0390201	-3.78243
AIF1L	-0.504765	11.556792	-6.100214	7.69E-08	1.55E-06	7.7638403
SLC25A26	-0.50452	6.5786289	-5.512006	7.42E-07	1.01E-05	5.564368
ACADM	-0.504167	9.9120825	-2.996448	0.0039329	0.0131388	-2.592452
MMAB	-0.504127	6.2423131	-7.403466	4.45E-10	2.20E-08	12.784647
MRPS24	-0.50397	6.9510282	-8.756668	2.06E-12	3.22E-10	18.037354
TUBB2A	-0.503899	9.4007325	-4.519257	2.87E-05	0.0002168	2.0398847
LOC1005061	-0.50382	5.2400092	-3.451466	0.0010131	0.0042533	-1.335953
PRF1	-0.503454	5.695457	-3.181188	0.0022981	0.0084309	-2.0975
ZSWIM6	-0.50337	10.266358	-4.099646	0.0001232	0.000734	0.6510208
ESD	-0.503302	13.44399	-7.504277	2.98E-10	1.60E-08	13.176815
FH	-0.503185	8.8549292	-5.349451	1.37E-06	1.70E-05	4.9687302
ABCC6P1	-0.503075	5.5413944	-2.94789	0.0045151	0.0147583	-2.718919
COX6A1	-0.503024	15.350382	-5.447634	9.47E-07	1.24E-05	5.3277693

PCDHB10	-0.502364	5.515432	-3.25266	0.0018574	0.0070685	-1.900327
PP7080	-0.502086	6.0694703	-4.245183	7.49E-05	0.0004819	1.1245463
TIMM22	-0.501875	7.9750895	-5.030113	4.52E-06	4.60E-05	3.8177664
EPDR1	-0.501494	7.2264377	-4.154104	0.0001024	0.0006276	0.8271371
CRB2	-0.500701	6.4236334	-5.755879	2.92E-07	4.66E-06	6.4686262
OTTHUMG00(	-0.500648	9.9565526	-3.535086	0.0007804	0.0034147	-1.091838
PAXBP1	0.5001433	6.0433864	7.8769913	6.75E-11	4.88E-09	14.627189
MGC16142	0.5005071	4.7160069	3.5559421	0.0007308	0.0032257	-1.030349
CSF1R	0.5005394	5.8663111	3.2755377	0.0017341	0.0066805	-1.836563
RUNX1-IT1	0.5007375	3.2580723	3.6150066	0.0006062	0.0027608	-0.854941
SNORD47	0.5009113	6.4897836	5.0084062	4.90E-06	4.92E-05	3.7405432
MPZL2	0.5009545	6.6530641	3.1068311	0.0028594	0.0101109	-2.299303
ANKRD61	0.5009565	4.0012925	5.3607297	1.32E-06	1.63E-05	5.0098605
OTTHUMG00(	0.5010021	3.8958121	5.1265007	3.16E-06	3.40E-05	4.1622933
OTTHUMG00(	0.5010155	4.1916031	4.7977236	1.06E-05	9.42E-05	2.9984071
RNA5SP370	0.5011175	5.5730407	3.6434293	0.0005537	0.0025542	-0.769867
SETBP1	0.5011344	6.0740766	4.1038266	0.0001215	0.0007256	0.6644939
SNORD114-3	0.5011762	4.4458236	2.5841398	0.0121483	0.033435	-3.615503
OTTHUMG00(	0.5013526	6.4201662	4.3270846	5.64E-05	0.000381	1.39495
CXCL6	0.5014056	4.5008789	2.5442838	0.0134732	0.0364774	-3.708069
ARHGAP26-I	0.502088	4.5890825	4.6969414	1.52E-05	0.0001277	2.6484072
PKD2	0.5029882	8.426587	4.5507508	2.57E-05	0.0001969	2.1469163
LINC00265	0.5034045	8.1293374	6.0038219	1.12E-07	2.11E-06	7.3993316
YTHDC2	0.5036144	6.9547544	9.8083588	3.37E-14	1.22E-11	22.050974
LINC00312	0.5039184	4.6090733	3.9302278	0.0002175	0.0011694	0.1116701
GOLGA8M	0.5041333	5.8527192	6.0093654	1.10E-07	2.07E-06	7.4202564
OTTHUMG00(	0.5044206	3.5585208	3.794731	0.0003398	0.0017034	-0.309935
RNA5SP483	0.5045999	4.1243533	4.1231443	0.0001138	0.0006867	0.7268532
CCDC14	0.5050097	6.8666148	5.7075112	3.52E-07	5.45E-06	6.2883418
LOC100505(	0.5050628	5.4050025	4.8197129	9.77E-06	8.83E-05	3.0752168
OTTHUMG00(	0.5050633	4.4584918	3.6574385	0.0005295	0.0024616	-0.727779
OTTHUMG00(	0.5052007	4.6800607	4.6801444	1.62E-05	0.0001343	2.5904066
SIK3-IT1	0.505244	5.7242272	4.0298159	0.000156	0.0008924	0.4271207
ADAM28	0.5054747	5.8405723	3.4058719	0.0011662	0.0047942	-1.467407
OTTHUMG00(	0.505786	4.094318	4.4727692	3.39E-05	0.0002491	1.8825696
STX19	0.505855	4.7500279	4.9058566	7.14E-06	6.77E-05	3.3776008
OTTHUMG00(	0.5058882	4.7245762	5.6497038	4.39E-07	6.53E-06	6.0734649
FAM41C	0.5066155	5.4449761	5.8911444	1.73E-07	3.05E-06	6.9750761
APOC1P1	0.5073143	4.8935972	3.4335199	0.0010709	0.0044569	-1.387835
ITGA4	0.5073675	5.0456105	3.1565935	0.0024712	0.008949	-2.164629
RASAL2	0.5077972	7.3467103	6.8008697	4.89E-09	1.58E-07	10.447393
RERG-AS1	0.5078526	5.4612885	3.8259713	0.0003068	0.0015611	-0.213525
OTTHUMG00(	0.507976	4.2361805	3.7691438	0.0003693	0.0018256	-0.388536
SYNPO2	0.5079865	6.6735556	4.6158694	2.04E-05	0.0001625	2.3693651
RNU7-71P	0.5081534	4.1172472	3.7647499	0.0003746	0.0018466	-0.402001
C1orf148	0.50853	4.8158508	4.2355518	7.74E-05	0.0004959	1.092929
FHL2	0.5085351	5.4814733	4.5724734	2.38E-05	0.0001846	2.2209519
SPON1	0.5090415	4.2574811	3.5147305	0.0008318	0.0036076	-1.151618
OR7E2P	0.5091515	4.5838561	4.7673985	1.18E-05	0.000103	2.8927385
ANKRD36BP2	0.5094677	4.5739526	6.7631735	5.68E-09	1.78E-07	10.301829
DHCR24	0.5094762	7.3147508	3.3124094	0.0015514	0.0060883	-1.733137

OTTHUMG000	0.5097304	4.7654807	5.9537227	1.36E-07	2.48E-06	7.2104431
OTTHUMG000	0.5104522	5.9881713	5.7022479	3.59E-07	5.53E-06	6.2687503
MIR147A	0.510482	3.9935774	5.0567265	4.10E-06	4.23E-05	3.9126314
RNA5SP99	0.5104931	4.0991713	4.9577687	5.90E-06	5.77E-05	3.5609336
RPS15AP10	0.5110955	4.3417787	4.0217928	0.0001602	0.0009121	0.401537
NAV2-AS1	0.5112296	8.5041498	2.7750862	0.0072977	0.0219329	-3.156254
HMBOX1-IT1	0.5121306	5.8285025	8.428866	7.52E-12	8.70E-10	16.770338
CCDC144CP	0.5124023	4.461728	4.4889253	3.20E-05	0.0002374	1.9371504
OTTHUMG000	0.5124152	3.5306362	2.8883005	0.0053387	0.0169669	-2.871998
SERTAD4	0.5124511	4.1623108	4.3650998	4.94E-05	0.0003408	1.521386
SLC5A4	0.5129103	4.8570695	3.5797001	0.000678	0.0030347	-0.960019
KCNN3	0.5131028	5.4400569	4.3450403	5.30E-05	0.0003608	1.4545975
TPSB2	0.5134008	4.1540797	3.543221	0.0007607	0.0033413	-1.067881
RNU6-18	0.5138555	11.355184	2.7967627	0.0068778	0.0208924	-3.102506
RNU6-17	0.5138555	11.355184	2.7967627	0.0068778	0.0208924	-3.102506
SNORD124	0.5139423	3.8071011	2.6911784	0.0091547	0.0264664	-3.361237
RPS20P22	0.5141048	4.1625308	4.9152873	6.90E-06	6.56E-05	3.4108453
IGFBP1	0.5141646	4.131017	3.2622303	0.0018048	0.0069033	-1.873691
MIR1976	0.5146353	5.4576169	5.4150251	1.07E-06	1.38E-05	5.2082757
OTTHUMG000	0.5146657	5.7223793	4.3265466	5.65E-05	0.0003814	1.3931648
PPP1R14A	0.5149226	8.8135295	3.2133776	0.0020887	0.0078016	-2.009082
RNU105B	0.5150382	3.8929252	3.6989568	0.0004634	0.0022065	-0.602441
CCL13	0.5151672	4.5334739	2.8248498	0.0063668	0.0195866	-3.032382
COL4A2	0.5152114	6.1275911	3.9916063	0.0001773	0.0009908	0.3055451
PGM5P2	0.5158435	5.3032515	7.4364062	3.91E-10	1.99E-08	12.912772
ANXA3	0.5158894	5.7199098	3.2925292	0.0016475	0.0064037	-1.789001
SOS1-IT1	0.516005	7.007679	5.4277828	1.02E-06	1.32E-05	5.2549974
KLHL4	0.5170118	3.9373962	3.8453605	0.0002879	0.001482	-0.153446
LRP1	0.5170205	6.9690751	3.3338106	0.0014538	0.0057773	-1.672739
MIR5480	0.517023	4.5600057	4.4560659	3.59E-05	0.0002622	1.8262446
CPVL	0.5170606	6.746927	3.0527158	0.0033459	0.011486	-2.44399
OTTHUMG000	0.5171952	3.7102515	7.3987115	4.54E-10	2.22E-08	12.766157
OTTHUMG000	0.5172341	4.4910802	6.1748485	5.75E-08	1.22E-06	8.0470003
LENG8	0.517304	8.8778626	4.7298718	1.35E-05	0.0001157	2.7623961
SDIM1	0.5173664	3.5632261	4.4452334	3.73E-05	0.0002705	1.7897732
MFSD2A	0.5174901	5.1835887	4.2392452	7.65E-05	0.0004906	1.1050488
C9orf84	0.5183701	4.6904287	5.5025303	7.69E-07	1.04E-05	5.5294823
OTTHUMG000	0.5185421	4.4568733	4.7351569	1.33E-05	0.0001139	2.7807243
HNRNPR	0.5185657	9.1042197	3.862706	0.0002719	0.0014104	-0.099544
SLC7A2	0.5187784	5.1310741	3.0922113	0.0029839	0.0104744	-2.338574
CD58	0.5203864	7.8059044	7.1943717	1.02E-09	4.37E-08	11.971956
RNU6-32	0.5216451	11.600286	2.9384172	0.0046376	0.015079	-2.743412
RNU6-13	0.5216451	11.600286	2.9384172	0.0046376	0.015079	-2.743412
RNU6-12	0.5216451	11.600286	2.9384172	0.0046376	0.015079	-2.743412
VN1R10P	0.5217031	3.8075744	9.1878556	3.77E-13	8.48E-11	19.69373
ENC1	0.5219923	6.0422328	3.5137584	0.0008344	0.0036166	-1.154468
AGAP11	0.5221785	5.3496749	6.7894788	5.11E-09	1.64E-07	10.403397
DOCK6	0.5221976	6.4139652	6.8394474	4.20E-09	1.38E-07	10.596465
MIR2276	0.5227593	4.3735123	3.1232212	0.0027256	0.0097233	-2.255116
LUC7L3	0.5228702	10.945157	6.0040251	1.12E-07	2.11E-06	7.4000984
SLC25A27	0.5230763	5.8548284	4.7449107	1.28E-05	0.0001104	2.8145749

RABGAP1L-I	0.5231022	5.0743243	5.9000201	1.67E-07	2.96E-06	7.0084194
RNY3P1	0.5231211	12.371641	3.6005172	0.0006348	0.0028676	-0.898145
CLK4	0.5231698	6.7664077	5.0629809	4.00E-06	4.15E-05	3.9349549
IGKV2D-30	0.5232254	4.0242066	2.8903835	0.0053077	0.016889	-2.866687
OTTHUMG00(	0.5232432	3.5382861	8.0483915	3.41E-11	2.76E-09	15.293733
C3orf79	0.5232986	3.9473723	3.1962083	0.002198	0.0081334	-2.056322
OTTHUMG00(	0.5233483	5.2925902	3.1864554	0.0022625	0.0083236	-2.083076
MIR221	0.5234143	3.8566561	3.9092454	0.0002332	0.0012382	0.0458011
SEL1L3	0.5234763	5.0730367	3.6166218	0.0006031	0.0027483	-0.850118
PLXDC2	0.5236011	10.908675	3.8605724	0.0002739	0.0014177	-0.106182
RNA5SP276	0.5237163	3.2598095	5.9485349	1.39E-07	2.52E-06	7.1909066
SNORA70E	0.5241208	4.5435554	3.9391474	0.0002112	0.0011432	0.1397343
IRX3	0.5243142	6.1718674	4.1504588	0.0001037	0.0006341	0.815307
OTTHUMG00(	0.5245401	3.9383948	3.5143074	0.0008329	0.0036119	-1.152858
LOC1005062	0.5249536	5.6046587	3.7300607	0.0004192	0.0020281	-0.507957
NPFF	0.5250226	4.951479	5.0971768	3.53E-06	3.73E-05	4.057203
NBPF24	0.5252188	11.440046	8.2859833	1.33E-11	1.32E-09	16.216432
RNA5SP408	0.5253649	6.2885936	4.9501849	6.07E-06	5.90E-05	3.5340999
RNA5SP263	0.525511	5.0410693	6.1369716	6.67E-08	1.38E-06	7.9032007
OTTHUMG00(	0.5266163	7.5443677	3.4915102	0.0008944	0.0038249	-1.219535
MEG3	0.5269442	5.8693326	3.2501102	0.0018717	0.0071079	-1.907415
MIR450A2	0.526945	3.5618831	4.3565762	5.09E-05	0.0003492	1.4929871
GPR56	0.5269971	9.7661607	2.6622859	0.0098881	0.0282746	-3.430676
GCNT7	0.5273964	4.225459	6.3144442	3.33E-08	7.81E-07	8.578606
RNA5SP33	0.5274671	4.7864561	3.3877101	0.0012332	0.0050246	-1.51944
RNU6-71P	0.5274728	4.1426664	4.5067284	3.00E-05	0.0002251	1.9974082
USP17L6P	0.5275275	4.5289502	4.2163738	8.27E-05	0.0005245	1.0300904
SNORA38	0.527653	3.8250775	3.7689696	0.0003695	0.0018261	-0.38907
RNA5SP355	0.5282409	4.9727702	4.5472295	2.60E-05	0.0001987	2.1349311
OTTHUMG00(	0.5283888	4.0760175	4.9440379	6.21E-06	6.00E-05	3.5123625
PTGES	0.5293101	4.6515423	6.2948417	3.59E-08	8.30E-07	8.5038084
GALNT1	0.5293629	8.4495713	3.9664973	0.0001928	0.0010633	0.2260205
OTTHUMG00(	0.5293739	4.7187682	7.5310076	2.68E-10	1.47E-08	13.280826
METTL21D	0.5294485	4.8403798	6.8358368	4.26E-09	1.40E-07	10.582508
CAPN6	0.5296676	4.2608231	4.0360375	0.0001527	0.0008779	0.4469797
OTTHUMG00(	0.5300883	3.8017989	4.1066476	0.0001203	0.0007197	0.67359
OTTHUMG00(	0.531669	5.7214243	3.9626465	0.0001953	0.0010751	0.2138503
OTTHUMG00(	0.5319997	7.0314946	3.0113314	0.003769	0.0126733	-2.55338
FNDC1	0.5322399	3.5832405	3.4381604	0.0010557	0.0044023	-1.374437
TNFRSF11B	0.5324893	8.7257997	2.8132941	0.0065727	0.0201357	-3.061298
MIR550A3	0.532633	3.9419152	3.2890842	0.0016647	0.0064592	-1.798658
NR1D2	0.5330139	8.6922057	4.9909285	5.22E-06	5.19E-05	3.6784649
MIR548L	0.533364	4.1491766	3.4014453	0.0011822	0.004849	-1.480106
OTTHUMG00(	0.5334709	11.216257	6.5480353	1.33E-08	3.62E-07	9.4732402
PLEKHO2	0.5336264	6.382293	5.8306712	2.19E-07	3.67E-06	6.7482559
DDR2	0.5341354	5.8266395	4.3539743	5.14E-05	0.0003515	1.4843237
SLC7A6	0.5343537	6.2890543	6.4897615	1.67E-08	4.42E-07	9.249519
IRF8	0.5352667	5.3467434	3.3587783	0.0013473	0.0054232	-1.601936
RNY4P10	0.5356417	6.551863	5.0118414	4.84E-06	4.88E-05	3.7527548
RNA5SP393	0.5356475	4.9482474	7.3865226	4.76E-10	2.30E-08	12.718753
OTTHUMG00(	0.536078	7.2588872	5.4893878	8.08E-07	1.09E-05	5.4811301

HEG1	0.5361529	12.455695	4.2425509	7.56E-05	0.0004855	1.1159007
MIR1972-1	0.5370317	4.3877162	5.2689752	1.86E-06	2.18E-05	4.6761738
MIR1972-2	0.5370317	4.3877162	5.2689752	1.86E-06	2.18E-05	4.6761738
HYMAI	0.5371427	4.2230693	4.6611305	1.73E-05	0.0001421	2.5248679
SNORD114-1	0.5373245	3.57103	3.1751862	0.0023392	0.0085504	-2.113916
KLRAP1	0.5374023	4.6211803	7.6736891	1.52E-10	9.18E-09	13.836083
HIGD1B	0.5374969	3.5388254	4.0018722	0.0001713	0.0009636	0.3381432
IGHV3-11	0.5375186	5.400631	3.2756577	0.0017334	0.006679	-1.836227
OTTHUMG00(	0.5380469	5.3139066	5.0074505	4.92E-06	4.94E-05	3.7371463
MIR197	0.5382807	5.7191352	6.125448	6.97E-08	1.43E-06	7.8594911
OTTHUMG00(	0.5384645	4.5009757	5.1364424	3.05E-06	3.30E-05	4.197976
ITGA5	0.5385207	6.8528861	6.2621239	4.09E-08	9.24E-07	8.3790712
SLC22A3	0.5387545	4.7179372	2.9089196	0.0050392	0.0161555	-2.819297
OTTHUMG00(	0.5390937	3.7085816	4.7930204	1.08E-05	9.54E-05	2.9819991
OTTHUMG00(	0.5393505	7.8115728	3.7119265	0.0004445	0.0021297	-0.563103
CACNA1C	0.5396538	5.4512031	7.5896876	2.12E-10	1.21E-08	13.509171
RNA5SP477	0.5397087	3.560817	4.2562396	7.21E-05	0.000467	1.1608879
SFRP1	0.539934	7.6220605	2.6367653	0.0105801	0.0298533	-3.491517
IFI16	0.5399567	10.223487	3.2507646	0.001868	0.0071	-1.905597
OTTHUMG00(	0.5402071	3.824219	5.2608997	1.92E-06	2.24E-05	4.6469059
AXL	0.5408254	8.1193326	2.4422677	0.0174801	0.0451313	-3.939676
LPP-AS1	0.5408424	3.598459	4.0315193	0.0001551	0.0008893	0.4325561
ITPKB	0.5410823	5.6462957	7.8174249	8.56E-11	5.90E-09	14.395435
OTTHUMG00(	0.5411725	4.1170354	3.260406	0.0018148	0.0069335	-1.878773
LOC541473	0.5415044	4.294502	6.5606247	1.26E-08	3.48E-07	9.5216156
TAS2R13	0.5415483	3.7736215	5.1807754	2.58E-06	2.88E-05	4.3574205
PPP1R14C	0.5419255	4.738698	3.194444	0.0022096	0.0081676	-2.061166
TCF4	0.5420311	8.9033625	5.1572748	2.82E-06	3.10E-05	4.272834
OTTHUMG00(	0.5424756	4.2758867	5.1942976	2.46E-06	2.76E-05	4.406158
MIR4482-1	0.5427469	3.8673861	3.129671	0.0026746	0.0095714	-2.237682
TAS2R3	0.5430378	4.2158226	5.9247215	1.52E-07	2.72E-06	7.1012846
CIITA	0.5433673	6.1647161	6.3104804	3.38E-08	7.92E-07	8.5634775
OTTHUMG00(	0.5438353	4.6747411	6.0241751	1.03E-07	1.99E-06	7.4761814
OTTHUMG00(	0.5440933	8.5507066	3.306057	0.0015815	0.0061861	-1.751013
MIR606	0.5449569	6.5798215	3.3581259	0.00135	0.0054307	-1.603791
S100A4	0.5457016	7.1029361	3.7710639	0.000367	0.0018157	-0.382649
MIR3619	0.5457261	6.435901	6.2348362	4.55E-08	1.02E-06	8.275139
MXRA5	0.54587	5.6722667	2.4983584	0.0151611	0.0401538	-3.813288
LINC00622	0.5460128	7.0655492	3.4961912	0.0008815	0.0037852	-1.205867
FRZB	0.5464863	11.22224	2.5411158	0.0135839	0.0367321	-3.715376
RNA5SP413	0.546557	5.477463	8.3780663	9.20E-12	9.98E-10	16.573504
BCAT1	0.5466147	4.0026233	4.0477171	0.0001468	0.0008491	0.4843085
MIR548AA1	0.5469881	4.2057572	5.2564633	1.95E-06	2.27E-05	4.6308338
OTTHUMG00(	0.5470079	3.8051485	4.5518535	2.56E-05	0.0001964	2.1506705
CRIP1	0.5470832	6.0027197	3.6863621	0.0004826	0.0022844	-0.640557
LOC100132ɛ	0.5474833	4.6412246	4.5175105	2.89E-05	0.0002179	2.0339601
ASH1L-IT1	0.5476258	5.6678093	4.6268634	1.96E-05	0.0001574	2.4070707
OTTHUMG00(	0.5476696	4.3500138	8.4037787	8.31E-12	9.30E-10	16.673146
OTTHUMG00(	0.5476833	4.9791805	7.3273623	6.03E-10	2.78E-08	12.488719
ABLIM1	0.5478721	6.7599154	3.4532305	0.0010075	0.0042344	-1.330842
OTTHUMG00(	0.5481014	4.2756385	4.2118717	8.40E-05	0.0005316	1.0153614

AGAP9	0.5493201	8.3356821	5.2571572	1.94E-06	2.26E-05	4.6333476
AHR	0.5493901	10.578348	3.7402736	0.0004056	0.001973	-0.476826
OTTHUMG00(	0.5496239	4.2299089	4.5916138	2.22E-05	0.0001743	2.2863283
SUZ12P1	0.5498185	9.4857226	7.6924376	1.41E-10	8.74E-09	13.909048
NUAK1	0.55018	5.5480951	6.5945402	1.11E-08	3.11E-07	9.6520118
MLLT11	0.5503807	5.3169785	4.6040129	2.13E-05	0.0001683	2.3287492
XIAP-AS1	0.5507813	10.993162	6.6168276	1.01E-08	2.90E-07	9.7377576
MIR1280	0.551007	5.8376239	5.5341515	6.82E-07	9.48E-06	5.6459735
RNA5SP191	0.5517104	4.9569748	4.7110184	1.45E-05	0.0001225	2.6970899
OTTHUMG00(	0.5517687	4.683753	6.0291332	1.01E-07	1.96E-06	7.4949118
OTTHUMG00(	0.5520616	4.2535303	4.1579716	0.000101	0.0006208	0.8396931
AHNAK2	0.5521655	3.9192936	4.43318	3.89E-05	0.0002798	1.7492438
CLEC10A	0.5523066	4.334031	3.8166677	0.0003163	0.0016012	-0.242287
OTTHUMG00(	0.5527187	4.1959815	3.7772524	0.0003597	0.0017858	-0.363663
OTTHUMG00(	0.5528099	4.3240167	6.5500688	1.32E-08	3.60E-07	9.4810528
OTTHUMG00(	0.553081	5.3822739	3.1458594	0.0025505	0.0091825	-2.193809
MIR1303	0.555186	13.742233	5.7996156	2.47E-07	4.06E-06	6.6320233
MIR3663	0.5558643	6.1881962	5.7223713	3.32E-07	5.20E-06	6.3436834
KITLG	0.5560488	6.0437362	3.104113	0.0028822	0.0101729	-2.306614
CCDC84	0.5562894	7.152953	6.1184327	7.17E-08	1.46E-06	7.8328905
LOC100289(	0.5564403	5.8481174	3.7292864	0.0004203	0.0020322	-0.510316
IGLJ7	0.5564706	7.9961657	4.3514266	5.18E-05	0.000354	1.4758434
OTTHUMG00(	0.5565102	4.2017298	2.8947566	0.0052432	0.0167104	-2.855527
USP17L23	0.5570961	5.4083716	3.1170291	0.0027755	0.0098729	-2.271829
ADAMTS3	0.5573527	4.3277246	3.3573937	0.001353	0.0054398	-1.605872
OTTHUMG00(	0.5574333	4.1809743	5.2507567	1.99E-06	2.31E-05	4.6101678
RNU6-31P	0.5574604	7.7265285	4.2745766	6.77E-05	0.0004431	1.2212729
OTTHUMG00(	0.5580327	3.5737892	8.6366257	3.30E-12	4.62E-10	17.574048
GOLGA8Q	0.5581119	7.9017554	5.3188143	1.54E-06	1.87E-05	4.8571684
OTTHUMG00(	0.5584796	4.3089095	6.311531	3.37E-08	7.89E-07	8.5674869
NBPF9	0.5593782	12.19267	8.2310747	1.65E-11	1.56E-09	16.003355
RNU6-15P	0.5596395	11.61987	3.0579332	0.0032958	0.0113516	-2.430122
RNA5SP282	0.5599583	3.8705664	3.8407819	0.0002923	0.0015012	-0.16765
HOXB-AS2	0.5599763	4.203708	5.0360711	4.42E-06	4.51E-05	3.8389869
OTTHUMG00(	0.5600716	4.633737	3.9513384	0.0002028	0.0011069	0.1781524
SFPQ	0.5602966	10.798733	7.0977147	1.50E-09	5.98E-08	11.596761
ANKRD50	0.56054	8.6813454	5.5974907	5.36E-07	7.70E-06	5.8799568
OTTHUMG00(	0.5608123	4.5209449	4.5572772	2.51E-05	0.0001934	2.1691416
OTTHUMG00(	0.5618973	3.7264777	4.9711245	5.62E-06	5.54E-05	3.6082323
SCUBE3	0.5619065	5.1899774	7.0167742	2.08E-09	7.70E-08	11.282884
PEAK1	0.561916	8.1934457	7.7367066	1.18E-10	7.71E-09	14.08133
RNU6-46P	0.5620899	5.3319411	4.6942355	1.54E-05	0.0001289	2.6390571
OTTHUMG00(	0.5629376	4.1017154	5.529008	6.95E-07	9.62E-06	5.62701
WFDC2	0.5632306	4.4848318	2.9377379	0.0046465	0.0151016	-2.745165
LOC643733	0.5634578	5.2024895	4.0719269	0.0001353	0.0007945	0.5618808
HERC2P9	0.5637816	7.9100111	7.5957417	2.07E-10	1.18E-08	13.532732
OTTHUMG00(	0.5641473	4.440402	5.0685055	3.92E-06	4.08E-05	3.9546827
STAG3L2	0.5647684	9.3544854	5.7949802	2.51E-07	4.11E-06	6.6146893
MIR3162	0.5649739	5.8745356	4.4806605	3.29E-05	0.0002436	1.9092168
MIR3192	0.5651753	5.3326659	3.576033	0.0006859	0.0030625	-0.970895
OTTHUMG00(	0.5654661	8.2454384	6.1973446	5.27E-08	1.14E-06	8.1324994

DMTF1	0.5657413	9.3043384	7.0899567	1.55E-09	6.09E-08	11.566663
OTTHUMG000	0.5657974	8.961028	5.6756094	3.97E-07	6.03E-06	6.1696779
IL10RB-AS1	0.5662945	7.5326187	4.8227094	9.67E-06	8.77E-05	3.0856957
LOC1005063	0.5663337	4.7652605	6.480664	1.73E-08	4.54E-07	9.214623
RNA5SP443	0.5664109	4.1692044	7.521008	2.79E-10	1.51E-08	13.241916
PECAM1	0.5666079	12.29751	3.5065748	0.0008533	0.0036808	-1.175506
MIR574	0.5667904	11.577052	7.1469795	1.24E-09	5.12E-08	11.787947
ASAP1	0.5670832	8.6342318	6.8553562	3.94E-09	1.32E-07	10.657968
SEC62-AS1	0.5672728	4.2040782	4.9593894	5.87E-06	5.75E-05	3.5666705
RBM25	0.5674147	9.0473061	8.9835809	8.41E-13	1.52E-10	18.910662
SEMA3D	0.5676128	4.2221552	4.1878941	9.12E-05	0.0005695	0.937061
HOXD10	0.5676223	6.2572241	3.1477761	0.0025361	0.0091404	-2.188604
TPSAB1	0.5686898	4.2678436	3.2328843	0.0019706	0.0074275	-1.955194
NEFM	0.5687602	4.3528325	4.9428403	6.23E-06	6.02E-05	3.5081289
NCAM1	0.5691783	5.0434034	5.7083892	3.50E-07	5.44E-06	6.2916103
OTTHUMG000	0.569498	3.9545713	6.8009935	4.89E-09	1.58E-07	10.447872
OTTHUMG000	0.5699327	11.27703	4.9991163	5.07E-06	5.06E-05	3.7075356
ANO1-AS1	0.5699987	6.1791479	2.9731124	0.0042034	0.0138878	-2.653421
PLA2G7	0.5700521	4.1546323	4.0795192	0.0001319	0.0007772	0.586262
LINC00473	0.5700682	4.8767861	2.7726221	0.0073469	0.0220507	-3.162344
GSN-AS1	0.5703697	4.8390838	5.411292	1.09E-06	1.40E-05	5.1946117
SLFN11	0.5708085	6.5033992	4.826491	9.54E-06	8.67E-05	3.0989243
UNC5B	0.5712539	7.7758961	5.8599572	1.95E-07	3.34E-06	6.8580214
MIR548AN	0.5713099	5.4276177	4.7083778	1.46E-05	0.0001234	2.6879528
NRP2	0.5713404	5.5587492	3.8394891	0.0002935	0.0015068	-0.171659
LYPD1	0.571918	5.3247579	4.0234366	0.0001593	0.0009078	0.4067762
EDN1	0.5722381	5.1203261	4.2772906	6.71E-05	0.0004396	1.2302224
TRIO	0.5725081	6.61091	7.4278838	4.04E-10	2.05E-08	12.879622
CRISPLD2	0.572615	4.7688369	3.3063936	0.0015799	0.0061821	-1.750067
MIR1228	0.5726941	6.6299361	8.1730649	2.08E-11	1.90E-09	15.77813
OTTHUMG000	0.5728296	4.4105616	4.5497134	2.58E-05	0.0001973	2.1433848
MIR1270-1	0.5728476	3.6732882	3.3795625	0.0012644	0.0051334	-1.542721
MIR1270-2	0.5728476	3.6732882	3.3795625	0.0012644	0.0051334	-1.542721
MIR566	0.5728833	15.360338	7.848062	7.57E-11	5.37E-09	14.514639
RNU7-77P	0.5730298	4.1420207	2.7946393	0.006918	0.0209896	-3.107785
OTTHUMG000	0.5731097	6.2673154	3.2644385	0.0017929	0.0068704	-1.867538
MAGI1-AS1	0.573444	4.4431536	4.5435773	2.64E-05	0.000201	2.1225051
OTTHUMG000	0.5735001	4.4343138	5.2319218	2.13E-06	2.45E-05	4.5420178
OSBPL3	0.5743657	5.0982841	5.6346493	4.65E-07	6.85E-06	6.0176145
FLNB	0.5744666	6.7867269	5.1075058	3.39E-06	3.62E-05	4.0941929
OTTHUMG000	0.5745699	4.1422993	4.563265	2.46E-05	0.0001899	2.1895465
ZBTB20-AS3	0.5748512	4.2930169	5.6562316	4.28E-07	6.39E-06	6.0976965
RNA5SP97	0.5752235	5.1011916	4.86089	8.41E-06	7.79E-05	3.2194667
RHOJ	0.5753715	6.7188656	4.7117561	1.44E-05	0.0001223	2.6996429
EMB	0.5759754	7.664018	2.6474597	0.0102849	0.0292013	-3.466078
CEP170P1	0.5764431	6.8398974	4.8957533	7.41E-06	6.98E-05	3.342016
OTTHUMG000	0.5770467	6.3266566	5.0444945	4.29E-06	4.39E-05	3.8690048
RAB32	0.578081	7.2716443	3.6500949	0.0005421	0.0025101	-0.749855
RNU7-55P	0.5781672	4.4287777	4.5942009	2.20E-05	0.0001732	2.2951751
MIR4663	0.5782	4.6163182	5.9860574	1.20E-07	2.24E-06	7.3323089
LINC00341	0.5785375	4.256237	5.0844313	3.70E-06	3.88E-05	4.0116004

GLIPR1	0.5795967	7.1753015	2.6316058	0.0107253	0.0301661	-3.503761
CEP170	0.5797062	7.0796656	7.0946689	1.52E-09	6.04E-08	11.584944
AMOTL1	0.5801058	9.2719207	5.9967707	1.15E-07	2.17E-06	7.3727224
OTTHUMG000	0.5815872	10.275179	4.7195718	1.40E-05	0.0001194	2.7267029
DIP2A-IT1	0.581937	4.1613825	5.6431337	4.50E-07	6.67E-06	6.0490849
LOC1010599	0.5821517	7.7349813	5.1812345	2.58E-06	2.88E-05	4.3590745
HRH1	0.5821779	5.0368144	4.7053582	1.48E-05	0.0001244	2.677507
OTTHUMG000	0.5822602	5.2828257	6.904134	3.25E-09	1.12E-07	10.846646
EPHB2	0.5824402	4.7343162	4.1731742	9.59E-05	0.000594	0.8891142
OTTHUMG000	0.5826465	8.2384469	6.9473661	2.73E-09	9.65E-08	11.013993
KCNT2	0.5826648	5.4203792	4.0687655	0.0001368	0.0008015	0.5517361
MIR15A	0.5830603	4.9701215	7.5108515	2.90E-10	1.56E-08	13.202397
RNA5SP439	0.5831219	3.7728154	3.9837984	0.000182	0.0010122	0.2807848
LINC00852	0.5832582	4.8343385	5.229442	2.15E-06	2.47E-05	4.5330519
SNORD114-2	0.5835692	3.738637	3.7190868	0.0004343	0.0020898	-0.541349
ITPR3	0.5843023	5.6854623	5.8348758	2.15E-07	3.62E-06	6.7640058
OTTHUMG000	0.5845745	3.9730243	5.5096795	7.48E-07	1.02E-05	5.5558003
FADS2	0.5845955	5.8777498	6.3756112	2.62E-08	6.44E-07	8.8122944
MIR1183	0.5850667	4.0247961	4.2100902	8.45E-05	0.0005342	1.0095352
OTTHUMG000	0.5852376	7.934013	4.8928264	7.49E-06	7.04E-05	3.331713
OTTHUMG000	0.5857176	3.796428	4.5365347	2.70E-05	0.0002053	2.0985576
OTTHUMG000	0.5868635	8.8534367	4.9071356	7.11E-06	6.74E-05	3.3821081
RGS17	0.5868761	4.5398567	8.5829687	4.09E-12	5.41E-10	17.366691
IGHA2	0.5869711	4.971827	4.220344	8.16E-05	0.0005184	1.0430865
SPIN4-AS1	0.588127	5.081352	4.4767499	3.34E-05	0.0002463	1.8960087
GNB4	0.5887903	7.1829138	7.3043392	6.61E-10	3.01E-08	12.399221
OTTHUMG000	0.5890094	4.816057	6.2621878	4.09E-08	9.24E-07	8.3793147
SNORA36C	0.5893638	4.1087843	3.8696344	0.0002658	0.0013835	-0.077973
RNU6-29	0.5897311	11.309702	3.4632528	0.0009767	0.0041235	-1.301779
OTTHUMG000	0.5898285	4.2447656	4.9056478	7.14E-06	6.77E-05	3.3768652
MLLT10P1	0.5901839	6.380623	3.7297199	0.0004197	0.00203	-0.508995
ZBTB20-AS1	0.5916121	4.4277954	6.0559691	9.14E-08	1.79E-06	7.5963557
ANO1	0.5920134	5.1865416	4.1790744	9.40E-05	0.0005836	0.9083217
NPIPA8	0.5924522	9.8624036	9.0344251	6.88E-13	1.28E-10	19.105858
RRM1-AS1	0.5935776	9.124221	7.1708842	1.12E-09	4.75E-08	11.880751
MIR4677	0.5937728	4.3313382	3.1220367	0.0027351	0.0097506	-2.258315
LGALS9	0.5943983	6.1254036	3.7436173	0.0004012	0.0019536	-0.466622
NNMT	0.594451	4.658438	3.2756997	0.0017332	0.006679	-1.83611
HIF1A	0.5953013	11.103166	3.8024724	0.0003313	0.0016677	-0.28609
OTTHUMG000	0.59551	4.0449608	5.1754596	2.64E-06	2.93E-05	4.3382743
P2RY14	0.5957859	4.2746438	3.4509277	0.0010148	0.0042593	-1.337512
OTTHUMG000	0.5962818	4.5298946	6.6063966	1.05E-08	2.99E-07	9.697621
RNA5SP474	0.5965545	4.2716384	4.3269222	5.65E-05	0.0003811	1.3944112
OTTHUMG000	0.5968446	4.2062874	4.8280688	9.48E-06	8.63E-05	3.1044451
MIR181A1HC	0.5969586	4.46269	6.7860376	5.18E-09	1.66E-07	10.390107
MIRLET7DHC	0.5970217	4.9103854	6.0213606	1.05E-07	2.00E-06	7.4655503
IGKV1-37	0.5971246	4.6006361	3.3727625	0.001291	0.0052297	-1.562122
SMC5	0.5978766	9.1159625	8.3082344	1.21E-11	1.23E-09	16.302747
ANKRD10	0.5978906	8.5773844	6.9925541	2.28E-09	8.29E-08	11.189025
OTTHUMG000	0.5983975	4.1641026	3.3628876	0.0013305	0.0053687	-1.590248
OTTHUMG000	0.5994231	4.4207339	7.7457561	1.14E-10	7.52E-09	14.116547

IGFBP3	0.5997315	6.3819867	4.2717607	6.84E-05	0.0004462	1.2119911
MIR1537	0.6004087	5.4945559	3.6728514	0.000504	0.0023642	-0.681354
OTTHUMG000	0.6007571	5.5237651	2.5948641	0.0118125	0.0326694	-3.590398
OTTHUMG000	0.6011333	3.8676754	4.1597846	0.0001004	0.0006171	0.8455814
OTTHUMG000	0.601424	5.3224918	3.7607471	0.0003795	0.0018674	-0.414258
RNU7-50P	0.6019231	5.1621157	5.5746149	5.85E-07	8.30E-06	5.795353
OTTHUMG000	0.60238	6.4154184	5.4168287	1.06E-06	1.37E-05	5.2148786
OTTHUMG000	0.6024226	3.8188795	5.2412721	2.06E-06	2.38E-05	4.5758385
MALL	0.6024576	6.8763875	3.2403341	0.0019272	0.0072858	-1.934553
OTTHUMG000	0.6026338	5.3813849	4.3074208	6.04E-05	0.0004028	1.3297777
RNY4P19	0.6035315	4.5387418	5.1900338	2.50E-06	2.80E-05	4.390785
MIR4440	0.6036516	6.123548	2.5469204	0.0133817	0.0362645	-3.701981
MIR575	0.6036905	3.8510977	3.6391463	0.0005613	0.0025832	-0.782715
OTTHUMG000	0.6039727	4.0573198	4.1386213	0.0001079	0.0006562	0.7769332
MYCBP2-AS2	0.6040191	4.5258456	3.4182338	0.0011227	0.0046373	-1.431883
EXT1	0.6045937	9.3241248	4.9562447	5.93E-06	5.79E-05	3.5555397
FAP	0.6047896	3.8599644	3.9321881	0.0002161	0.0011635	0.1178349
OTTHUMG000	0.6051587	4.43759	3.7930113	0.0003417	0.001711	-0.315228
INSL4	0.6052557	4.0644887	3.3600773	0.001342	0.0054074	-1.598242
BRWD1-IT1	0.6056625	4.2645016	5.8778974	1.82E-07	3.18E-06	6.925336
MACF1	0.6057998	10.987787	7.600592	2.03E-10	1.16E-08	13.551607
DAPK1-IT1	0.6062275	6.9763349	7.7714044	1.03E-10	6.88E-09	14.216357
CD84	0.6065659	3.9594498	3.3350378	0.0014484	0.0057588	-1.669267
OTTHUMG000	0.6070258	5.329752	4.3942522	4.46E-05	0.0003123	1.6187344
OGN	0.6073722	4.5759628	3.9927657	0.0001766	0.000988	0.3092242
HOXA11	0.6083894	5.3521405	4.3625858	4.99E-05	0.0003432	1.5130069
OTTHUMG000	0.6085949	4.5884766	3.9620414	0.0001957	0.0010769	0.2119387
MYH10	0.6087403	8.1702213	4.2750803	6.76E-05	0.0004425	1.2229337
MIR3125	0.6091979	4.9357879	3.9153652	0.0002285	0.0012184	0.0649911
OMG	0.609452	4.1941816	5.9426734	1.42E-07	2.56E-06	7.1688382
LINC00969	0.6096014	6.7826541	5.515839	7.31E-07	1.00E-05	5.5784843
OTTHUMG000	0.6100058	5.0140679	3.0263839	0.0036096	0.0122146	-2.51372
OTTHUMG000	0.6100417	4.2037375	3.270878	0.0017585	0.0067591	-1.849576
SERPINE1	0.6102464	5.5937741	2.7405756	0.008015	0.0237288	-3.241156
OTTHUMG000	0.610255	5.2101554	4.5421462	2.65E-05	0.0002019	2.1176373
HDAC9	0.6109382	4.7298236	7.9350315	5.36E-11	4.00E-09	14.852958
CRLF3	0.6110182	7.5281274	6.7654649	5.62E-09	1.77E-07	10.310675
RNA5SP194	0.6110712	3.6223825	6.3273792	3.16E-08	7.50E-07	8.6279875
STOX2	0.6113322	6.0428244	6.3963169	2.41E-08	5.99E-07	8.8914976
MIR1233-1	0.6122597	4.5492698	7.4386918	3.87E-10	1.98E-08	12.921663
MIR1233-2	0.6122597	4.5492698	7.4386918	3.87E-10	1.98E-08	12.921663
OTTHUMG000	0.6124496	5.1036239	4.4470231	3.71E-05	0.0002692	1.7957957
KIAA0196-A	0.6125756	5.3210007	6.3520246	2.87E-08	6.97E-07	8.7221303
TNFRSF21	0.6126861	9.3390154	2.8062104	0.0067019	0.0204677	-3.078978
MIR3200	0.6137856	2.8461549	3.313264	0.0015474	0.0060772	-1.730731
CLK1	0.6139059	10.332103	4.1924775	8.98E-05	0.000562	0.9520095
C1orf186	0.6152605	7.0324157	2.8783309	0.0054894	0.0173571	-2.897378
PTGS1	0.6160042	5.9809616	4.7622954	1.20E-05	0.0001046	2.8749862
TIA1	0.6167475	8.6253097	6.8504824	4.02E-09	1.34E-07	10.639124
PGAM1P5	0.6167549	6.3229202	5.2238057	2.20E-06	2.51E-05	4.5126796
AHSA2	0.6183347	7.730079	6.7047096	7.15E-09	2.16E-07	10.076281

LOC100996[illegible]	0.61871	5.6234923	5.1552714	2.84E-06	3.11E-05	4.2656302
HERC2P2	0.619122	7.7077292	7.8694925	6.95E-11	5.02E-09	14.598016
RNA5SP110	0.6192198	3.9015434	4.032496	0.0001546	0.0008871	0.4356732
UBASH3B	0.6192658	5.5461084	5.0234686	4.63E-06	4.70E-05	3.7941143
OTTHUMG000[illegible]	0.6200064	3.9547016	5.4989553	7.80E-07	1.05E-05	5.5163256
OTTHUMG000[illegible]	0.6201647	5.4451175	7.4787064	3.30E-10	1.73E-08	13.077329
RNU6-49	0.6202604	6.2151403	5.702065	3.59E-07	5.54E-06	6.2680698
MFGE8	0.62055	6.4670325	3.7001778	0.0004616	0.0021986	-0.598741
MAK	0.6207135	4.6974274	8.6356991	3.32E-12	4.62E-10	17.570468
MAP3K1	0.6215172	8.1032656	5.0711088	3.88E-06	4.05E-05	3.9639818
OTTHUMG000[illegible]	0.6220748	4.4294679	4.7864087	1.10E-05	9.74E-05	2.9589449
MIR4659A	0.62272	4.5010095	2.6258409	0.0108896	0.0305532	-3.517419
LOC731275	0.6232608	9.504369	5.3912309	1.17E-06	1.49E-05	5.1212374
OTTHUMG000[illegible]	0.6240581	5.9611949	6.093876	7.88E-08	1.59E-06	7.7398327
DEFB109P1	0.6243808	6.0122033	3.5329096	0.0007857	0.0034328	-1.098239
MIR3679	0.624484	5.7311207	3.5677148	0.0007042	0.0031296	-0.995537
OTTHUMG000[illegible]	0.6248968	5.0994056	3.7836907	0.0003522	0.001755	-0.34389
VCAN-AS1	0.6252471	4.5307056	3.7802325	0.0003562	0.0017714	-0.354513
LUC7L	0.6255453	7.657443	9.1144176	5.03E-13	1.04E-10	19.412575
ADAMTS6	0.6256515	4.0730598	5.467576	8.78E-07	1.17E-05	5.4009677
OTTHUMG000[illegible]	0.626203	4.6471256	7.0935769	1.53E-09	6.05E-08	11.580708
RNA5SP490	0.6269622	5.2338503	4.6598723	1.74E-05	0.0001425	2.5205353
RNA5SP297	0.6273496	4.1535987	5.1268259	3.16E-06	3.40E-05	4.1634602
DOCK10	0.6276165	6.3407151	3.4364597	0.0010612	0.0044214	-1.379349
MIR181B2	0.6276165	3.8302431	3.1483428	0.0025319	0.0091284	-2.187065
OTTHUMG000[illegible]	0.627723	4.8240807	4.9827568	5.38E-06	5.33E-05	3.6494709
RNU6-20	0.6280398	10.760454	4.732766	1.34E-05	0.0001148	2.7724318
MARVELD1	0.6283455	6.9914046	5.2774988	1.80E-06	2.13E-05	4.7070839
MTHFD1L	0.6283869	5.0002174	6.1216756	7.08E-08	1.45E-06	7.845186
CDH2	0.6285721	7.6240764	2.925452	0.0048103	0.0155343	-2.776837
OTTHUMG000[illegible]	0.6291469	4.2999967	4.7853462	1.11E-05	9.76E-05	2.9552414
AGAP5	0.6300903	8.2438113	7.5707322	2.29E-10	1.28E-08	13.435406
CD24	0.6303378	10.614328	2.9809621	0.0041105	0.0136291	-2.632951
SLITRK4	0.6305552	4.3427513	4.1841077	9.24E-05	0.0005755	0.924719
STARD4-AS1	0.6309573	5.7837856	5.390183	1.18E-06	1.49E-05	5.1174075
MIR619	0.6322288	4.6341516	5.1525046	2.87E-06	3.14E-05	4.2556829
LAMC1	0.6324238	8.7703943	4.3127461	5.93E-05	0.0003973	1.3474121
TGFB3	0.6330378	4.5743544	5.1778352	2.61E-06	2.91E-05	4.3468297
OTTHUMG000[illegible]	0.6332987	4.0229113	4.3485583	5.24E-05	0.0003572	1.4662991
SNORD16	0.6338561	3.6738644	3.9546211	0.0002006	0.001098	0.188509
MIR1255B2	0.634924	4.3485497	5.8112658	2.36E-07	3.91E-06	6.6756067
LAMC2	0.6350688	4.57251	3.8109139	0.0003223	0.0016278	-0.260054
OTTHUMG000[illegible]	0.6353721	6.9586684	6.3209383	3.25E-08	7.67E-07	8.6033958
OTTHUMG000[illegible]	0.6356764	4.7698239	3.2490542	0.0018776	0.0071278	-1.910349
FLJ31306	0.6360868	8.6382062	8.8447721	1.45E-12	2.41E-10	18.37684
RNA5SP465	0.6364403	3.1385303	4.6526517	1.79E-05	0.0001457	2.4956827
RNU6-7	0.6365291	11.291119	3.6515522	0.0005395	0.002501	-0.745476
LOC100996[illegible]	0.6366271	4.4620254	6.062577	8.91E-08	1.76E-06	7.6213511
PAPPA2	0.6368383	5.3779079	2.465197	0.0164961	0.0430476	-3.888293
SCARNA5	0.6368805	6.7371172	2.8063848	0.0066987	0.0204666	-3.078544
ADAM10	0.6373468	10.200312	5.7528559	2.95E-07	4.71E-06	6.4573447

SACS	0.6373491	5.4696272	4.3945166	4.46E-05	0.0003121	1.6196187
MATN2	0.6380789	7.0829897	4.6944915	1.54E-05	0.0001288	2.6399418
LINC00260	0.6385344	4.1541674	5.0414083	4.34E-06	4.43E-05	3.8580046
IL18	0.6388647	6.6048589	3.109603	0.0028363	0.0100467	-2.291842
OTTHUMG000	0.6393416	5.4339369	3.8943922	0.000245	0.001291	-0.000699
SNAR-D	0.6400005	5.8534228	7.0496291	1.82E-09	6.99E-08	11.410254
EBLN2	0.6401302	4.8067625	6.9627942	2.57E-09	9.15E-08	11.07374
PRO0611	0.6409186	5.6339789	3.6090972	0.0006177	0.0028032	-0.872575
CADM3	0.6414775	5.018788	3.2704735	0.0017607	0.0067654	-1.850705
OTTHUMG000	0.6420032	5.0013903	3.7265538	0.000424	0.0020483	-0.518635
LOC1001289	0.6421029	4.7995293	5.8194908	2.28E-07	3.82E-06	6.706391
RNU7-73P	0.6428897	3.6531402	5.9300942	1.49E-07	2.67E-06	7.1214966
LOC647859	0.643005	7.0777367	2.8873746	0.0053525	0.017001	-2.874358
DKK3	0.6432686	6.8396093	5.0991661	3.50E-06	3.71E-05	4.0643246
MIR4742	0.6433908	4.447779	4.9402379	6.29E-06	6.07E-05	3.4989307
RSF1-IT2	0.6450271	4.5624613	5.6024402	5.26E-07	7.58E-06	5.8982763
IGKV4-1	0.6452519	4.4610349	2.4115918	0.0188788	0.0481197	-4.007801
RNA5SP93	0.6457934	4.2073903	5.8419716	2.09E-07	3.54E-06	6.7905928
ITCH-IT1	0.6464287	4.0545689	4.7108829	1.45E-05	0.0001225	2.6966211
GPR65	0.6469015	6.5952397	2.8039342	0.0067439	0.0205725	-3.084652
C8orf4	0.6471687	14.042967	3.1489703	0.0025272	0.0091148	-2.18536
SRPX2	0.6486415	4.4675097	5.6779724	3.94E-07	5.98E-06	6.1784605
CORO1C	0.6490628	8.8643223	5.2713474	1.84E-06	2.17E-05	4.6847745
HEYL	0.6491211	5.2947775	5.2094621	2.32E-06	2.62E-05	4.4608721
FAM133CP	0.6493206	4.8812631	4.3731306	4.81E-05	0.0003329	1.5481698
RNA5SP395	0.6498612	5.0380815	6.0424475	9.63E-08	1.88E-06	7.5452284
RNA5SP440	0.6502701	5.3852818	6.4419379	2.02E-08	5.16E-07	9.0661732
ANTXR1	0.6507616	7.7539507	5.1753626	2.64E-06	2.93E-05	4.3379248
RNA5SP161	0.6507805	4.7972289	3.4927898	0.0008909	0.0038155	-1.2158
MIRLET7G	0.650872	3.5034279	5.7565664	2.91E-07	4.65E-06	6.4711913
MIR4472-2	0.6511294	13.569339	6.1395667	6.60E-08	1.37E-06	7.9130469
TRIM25	0.6512276	8.9593684	6.2751755	3.88E-08	8.86E-07	8.4288145
LIMA1	0.6516166	9.7321718	9.0007189	7.86E-13	1.44E-10	18.976477
GLIPR2	0.6520569	6.0828338	3.9983421	0.0001733	0.0009735	0.3269282
OTTHUMG000	0.6521201	4.2452762	6.091649	7.95E-08	1.60E-06	7.7313976
OTTHUMG000	0.6544273	6.0308815	8.7335796	2.25E-12	3.46E-10	17.948312
TAS2R5	0.6544555	4.2936625	6.1976993	5.26E-08	1.14E-06	8.1338481
OTTHUMG000	0.6544595	3.7232731	6.9600493	2.60E-09	9.23E-08	11.063109
NEGR1	0.6545213	4.5487307	3.9596827	0.0001972	0.0010842	0.2044883
PDGFRA	0.6552304	7.2589838	2.9747792	0.0041835	0.0138331	-2.649078
LOC728095	0.6553163	4.000202	6.7406413	6.20E-09	1.92E-07	10.214871
LOC1010606	0.6554764	9.6346926	9.138446	4.57E-13	9.75E-11	19.504613
RNA5SP88	0.656096	4.208448	4.0503104	0.0001456	0.0008428	0.4926052
OTTHUMG000	0.6577127	5.0242202	4.5486039	2.59E-05	0.000198	2.1396083
NEXN-AS1	0.6581323	3.9000162	7.7965997	9.30E-11	6.30E-09	14.314401
VASH1	0.6582985	5.4070534	9.4640725	1.28E-13	3.48E-11	20.747293
LOC388692	0.6584825	5.3616421	7.6404849	1.73E-10	1.02E-08	13.706859
OTTHUMG000	0.6591913	4.9828585	5.3428271	1.41E-06	1.73E-05	4.9445916
OTTHUMG000	0.6592128	5.910372	7.1566225	1.19E-09	4.96E-08	11.82538
PCED1B-AS1	0.659399	6.2401216	6.684949	7.73E-09	2.32E-07	10.000107
THBD	0.6595641	5.9420946	5.0551898	4.12E-06	4.25E-05	3.9071485

MIR509-1	0.6604605	4.6323446	2.5227433	0.0142425	0.038126	-3.757613
RNU6-69P	0.660565	3.8325623	5.450274	9.38E-07	1.23E-05	5.3374561
OGT	0.6619532	9.3669079	7.1229466	1.36E-09	5.50E-08	11.694667
RNU6-51	0.6621873	5.4545072	3.0834509	0.0030609	0.0106995	-2.362042
RNU6-19P	0.6628804	9.3160775	4.8747216	8.00E-06	7.44E-05	3.2680412
EPHA3	0.6631656	4.2372538	3.7086779	0.0004492	0.0021498	-0.572965
OTTHUMG000	0.6643818	5.2810782	7.0422227	1.88E-09	7.11E-08	11.381537
SH3BP5-AS1	0.6650897	6.6405495	6.1745407	5.76E-08	1.22E-06	8.045831
GOLGA6L4	0.6664417	6.2183777	7.3725542	5.04E-10	2.40E-08	12.664432
MIR4796	0.6668349	3.6740115	3.9954696	0.000175	0.0009813	0.3178066
FOLR2	0.6671497	4.8521746	3.1276849	0.0026902	0.0096179	-2.243053
RNA5SP290	0.6672358	4.786422	6.9318503	2.91E-09	1.01E-07	10.95392
MRC2	0.6675396	6.1732716	4.5201015	2.87E-05	0.0002162	2.0427502
ALG13-AS1	0.6679514	6.270932	7.1140123	1.41E-09	5.65E-08	11.659997
KCTD12	0.6679976	11.098301	4.4998324	3.08E-05	0.0002301	1.9740533
RNA5SP295	0.6685038	5.3676762	4.6004766	2.15E-05	0.0001701	2.3166451
MIR204	0.6685193	3.5774921	3.645099	0.0005508	0.0025425	-0.764857
ARHGAP15	0.6687375	5.2099938	3.3962096	0.0012014	0.0049178	-1.495113
CACNA1C-IT	0.6688594	3.6542057	6.3000369	3.52E-08	8.18E-07	8.5236271
OTTHUMG000	0.6706856	9.4792472	7.4510309	3.68E-10	1.90E-08	12.969663
SKIL	0.6708363	11.562	9.020625	7.27E-13	1.34E-10	19.052896
TCF7L1-IT1	0.6720512	5.1171785	4.5076047	3.00E-05	0.0002247	2.0003775
MILR1	0.6729658	4.8663952	4.8800166	7.85E-06	7.33E-05	3.2866522
OTTHUMG000	0.6733259	3.7958733	5.8783427	1.82E-07	3.18E-06	6.9270073
OTTHUMG000	0.6734195	4.3149548	4.6189554	2.02E-05	0.000161	2.3799445
ADAMTSL3	0.6741945	4.4111446	4.2419275	7.58E-05	0.0004864	1.1138539
RNU6-16P	0.6749969	9.6498125	3.5649597	0.0007103	0.003151	-1.003691
NBPF16	0.6755365	11.391354	9.9890393	1.68E-14	7.67E-12	22.730532
MIR126	0.6760431	3.5298111	3.3045436	0.0015888	0.0062121	-1.755269
MIR3942	0.6763485	4.4893259	3.1991306	0.002179	0.0080767	-2.048294
OTTHUMG000	0.6763536	5.7828603	7.5353867	2.63E-10	1.44E-08	13.297865
OTTHUMG000	0.6763536	5.7828603	7.5353867	2.63E-10	1.44E-08	13.297865
CPE	0.676387	5.3998257	4.6138753	2.05E-05	0.0001635	2.3625303
MIR1-2	0.6774614	4.2174649	6.0719347	8.59E-08	1.70E-06	7.6567595
RNA5SP434	0.6777059	3.8885262	6.2161296	4.89E-08	1.07E-06	8.2039454
RPL23AP64	0.6778521	6.365071	5.2604176	1.92E-06	2.24E-05	4.645159
OTTHUMG000	0.6788079	4.4102754	4.4441265	3.75E-05	0.0002713	1.7860487
RNU7-21P	0.6794343	4.252441	6.1418884	6.54E-08	1.36E-06	7.9218562
RNA5SP247	0.6795627	3.8661533	3.4930606	0.0008901	0.0038128	-1.215009
C9orf131	0.6796543	4.1831589	9.1529263	4.32E-13	9.28E-11	19.560056
OTTHUMG000	0.6802179	6.6196911	6.2686056	3.98E-08	9.06E-07	8.4037722
COL4A2-AS1	0.6810629	5.3343961	5.4373511	9.85E-07	1.28E-05	5.2900631
OTTHUMG000	0.6811345	4.4024557	5.8699381	1.88E-07	3.25E-06	6.8954644
OTTHUMG000	0.6814792	6.2049949	8.5360626	4.92E-12	6.33E-10	17.185297
XAF1	0.6828246	6.9419977	3.7874719	0.0003479	0.0017369	-0.332267
OTTHUMG000	0.6834198	5.8433448	7.2704828	7.56E-10	3.37E-08	12.267637
OTTHUMG000	0.6836889	7.9090246	5.9454743	1.40E-07	2.55E-06	7.1793827
MIR509-2	0.6838356	4.9376772	2.546618	0.0133921	0.0362865	-3.702679
CLU	0.6839418	7.6952051	3.1042463	0.002881	0.0101729	-2.306255
RNA5SP129	0.6842532	5.1392869	3.6824776	0.0004887	0.0023074	-0.652296
OTTHUMG000	0.6849688	4.6314584	4.6797519	1.62E-05	0.0001344	2.5890522

SMAD9-AS1	0.6850252	5.2118528	3.7661978	0.0003729	0.0018397	-0.397565
MYOZ2	0.6850782	7.9427344	3.0250577	0.0036234	0.0122532	-2.51722
GOLGA6L9	0.6855656	5.6239585	8.3295006	1.12E-11	1.15E-09	16.385222
CBFB	0.6865685	8.7561541	6.4171896	2.22E-08	5.57E-07	8.9713878
SNORD113-3	0.6875747	4.8697774	3.349969	0.001384	0.0055442	-1.626959
MAP4K4	0.6875929	9.5072251	7.2393692	8.56E-10	3.77E-08	12.146742
CHRM3	0.6878665	6.046699	5.9294575	1.49E-07	2.68E-06	7.1191009
TNXB	0.6886171	5.054728	6.4083185	2.30E-08	5.73E-07	8.9374277
LOC1009963	0.6889017	5.7987813	7.2964837	6.82E-10	3.10E-08	12.368688
BACH1-IT3	0.690349	4.223408	4.6491426	1.81E-05	0.0001472	2.4836114
OTTHUMG000	0.6909207	7.0843393	5.7645044	2.82E-07	4.54E-06	6.5008219
ABCA8	0.6911781	4.4482093	3.5483509	0.0007485	0.0032967	-1.052757
OTTHUMG000	0.6913294	4.36176	5.5492004	6.44E-07	9.01E-06	5.701489
PNISR	0.6919657	10.001278	8.3980141	8.50E-12	9.38E-10	16.650809
FKSG62	0.6927012	12.758736	7.6914807	1.41E-10	8.74E-09	13.905324
OTTHUMG000	0.6929685	4.4837477	4.6041967	2.13E-05	0.0001683	2.3293785
ANKRD36BP1	0.6933833	11.078879	5.4667104	8.81E-07	1.17E-05	5.3977888
ITCH-AS1	0.6937175	7.0806502	7.7050976	1.34E-10	8.39E-09	13.958317
IL32	0.6947271	7.7960452	3.2623619	0.0018041	0.0069014	-1.873325
UCHL1	0.6952986	10.339398	3.835506	0.0002974	0.0015221	-0.184004
DACT1	0.696652	4.987361	5.26621	1.88E-06	2.20E-05	4.6661502
MIR181A2HO	0.697543	5.0169579	4.3296737	5.59E-05	0.0003782	1.4035428
NPIPL1	0.6981289	7.6820802	8.0563845	3.30E-11	2.69E-09	15.324801
TAS2R43	0.6990345	5.4743269	5.8520692	2.01E-07	3.43E-06	6.8284421
OTTHUMG000	0.699828	6.0001992	5.4534144	9.27E-07	1.22E-05	5.3489784
OTTHUMG000	0.6998666	5.3748202	3.8159458	0.000317	0.0016044	-0.244517
LOC541471	0.7004919	5.8479764	7.2917116	6.95E-10	3.15E-08	12.35014
OTTHUMG000	0.7007405	5.0424905	6.0119743	1.08E-07	2.05E-06	7.4301058
ADAM15	0.7008291	6.9720851	7.1693053	1.13E-09	4.77E-08	11.87462
OTTHUMG000	0.701304	5.7520548	7.4019102	4.48E-10	2.20E-08	12.778597
NMU	0.7014594	4.1840467	3.0349766	0.0035215	0.011957	-2.491014
OTTHUMG000	0.7016495	4.784032	3.8363568	0.0002965	0.0015191	-0.181368
DDX17	0.7027111	13.365159	7.8928395	6.34E-11	4.61E-09	14.688842
OTTHUMG000	0.7030721	4.5244621	7.1641197	1.16E-09	4.83E-08	11.854487
OTTHUMG000	0.703389	4.6826287	4.3476676	5.25E-05	0.000358	1.4633359
MIR626	0.703508	4.0081721	4.4411611	3.79E-05	0.0002738	1.7760739
FKBP1AP1	0.704718	5.5220464	6.3350905	3.07E-08	7.33E-07	8.6574362
ADAM12	0.7047671	4.8185039	3.8840553	0.0002535	0.0013304	-0.032999
LOC1002879	0.7052728	5.1115998	8.6661068	2.94E-12	4.27E-10	17.687909
DLEU2L	0.705611	4.6295836	6.8371102	4.23E-09	1.40E-07	10.58743
IGHV7-81	0.7063132	3.9727451	4.042694	0.0001494	0.0008611	0.4682469
OTTHUMG000	0.7070315	7.5960474	8.5591904	4.49E-12	5.85E-10	17.274751
LOC644135	0.7074459	4.642343	7.6201943	1.88E-10	1.09E-08	13.627893
HERC2P7	0.7078302	8.5117382	5.1602193	2.79E-06	3.07E-05	4.2834244
RNA5SP70	0.7078626	4.4972795	6.8427896	4.14E-09	1.37E-07	10.609384
DCUN1D2-AS	0.7084644	3.959852	6.5859959	1.14E-08	3.20E-07	9.6191512
FKSG52	0.7084712	14.926366	8.7150001	2.42E-12	3.65E-10	17.876633
SLC4A7	0.7084781	5.0156692	4.7109239	1.45E-05	0.0001225	2.6967628
OTTHUMG000	0.7087246	3.6158374	6.320392	3.25E-08	7.68E-07	8.60131
ADH1C	0.7100659	5.7092849	2.8312505	0.0062554	0.0193014	-3.016327
CASP3	0.7102284	8.3434541	7.5149757	2.86E-10	1.54E-08	13.218444

MIR5095	0.7103712	14.674343	7.3641906	5.21E-10	2.46E-08	12.63191
MEST	0.7108255	5.421381	4.6419177	1.86E-05	0.0001505	2.4587711
CYP1B1	0.711929	11.305263	3.5232294	0.00081	0.0035222	-1.126686
COL4A1	0.7121019	7.9304764	3.3900048	0.0012245	0.0049952	-1.512876
ISLR	0.7123766	4.4854805	6.0721274	8.58E-08	1.70E-06	7.6574887
MIR1229	0.7139394	7.2766049	7.7126461	1.30E-10	8.21E-09	13.987694
EPB41L2	0.7146298	8.805002	6.7431226	6.14E-09	1.91E-07	10.224445
CCDC144B	0.7148639	5.5661728	4.8251515	9.58E-06	8.70E-05	3.0942381
OTTHUMG00(	0.7154203	3.9200023	6.013557	1.08E-07	2.05E-06	7.4360814
GOLGA6L10	0.7161349	6.3128766	6.9949504	2.26E-09	8.24E-08	11.19831
RNU6-66P	0.7161691	4.8593261	6.3275569	3.16E-08	7.50E-07	8.6286661
OTTHUMG00(	0.7163218	4.9113772	4.6606929	1.74E-05	0.0001423	2.523361
GK-AS1	0.7169736	4.1649613	3.6492474	0.0005435	0.0025154	-0.7524
BMP2KL	0.7173457	5.0928684	6.8472525	4.07E-09	1.36E-07	10.626637
NEFL	0.7175497	5.495358	3.7773157	0.0003596	0.0017857	-0.363469
OTTHUMG00(	0.71843	4.6142592	4.8309748	9.38E-06	8.56E-05	3.1146151
OTTHUMG00(	0.7193259	4.2040113	4.2345719	7.77E-05	0.000497	1.0897147
OTTHUMG00(	0.7198967	6.272049	3.4643785	0.0009733	0.0041114	-1.298511
OTTHUMG00(	0.720719	4.0255377	5.689957	3.76E-07	5.76E-06	6.2230214
MIR548I2	0.7222411	6.1023252	4.8818611	7.79E-06	7.29E-05	3.2931375
MAMDC2	0.7230104	5.0664408	4.9566044	5.93E-06	5.78E-05	3.556813
OTTHUMG00(	0.7232243	5.4225251	3.5046009	0.0008586	0.0037009	-1.181282
RNF213	0.7244417	8.4858489	7.3750902	4.99E-10	2.38E-08	12.674294
GOLGA6L5	0.7255447	6.2428182	6.4609816	1.87E-08	4.86E-07	9.1391541
TAS2R14	0.7255909	5.0102957	4.9994546	5.06E-06	5.06E-05	3.708737
OTTHUMG00(	0.7261176	4.3466662	5.4012139	1.13E-06	1.44E-05	5.1577389
FMNL3	0.7261374	7.6168375	7.9748059	4.57E-11	3.48E-09	15.007642
OTTHUMG00(	0.7262884	5.7347651	7.8919327	6.36E-11	4.61E-09	14.685314
MIR145	0.7268334	5.0271574	7.4917091	3.13E-10	1.66E-08	13.127918
EDNRA	0.7274085	7.4976744	3.274507	0.0017395	0.0066939	-1.839442
SMG1	0.7274714	8.7035597	9.881697	2.54E-14	1.02E-11	22.327205
MIR548U	0.7277056	5.9796546	5.7233889	3.31E-07	5.18E-06	6.3474747
LARGE-IT1	0.728414	4.9992648	4.8017583	1.04E-05	9.31E-05	3.012489
TRAJ38	0.7285777	3.6222128	3.9075714	0.0002345	0.001244	0.0405552
LOC389831	0.7297121	9.4595143	3.7163029	0.0004382	0.0021046	-0.54981
FABP4	0.7305535	6.4116733	2.5104991	0.0146973	0.0391434	-3.785623
LAMA2	0.7307161	5.6310385	3.9290641	0.0002184	0.0011727	0.1080117
OTTHUMG00(	0.7311186	5.2594436	6.8717342	3.69E-09	1.25E-07	10.721303
OTTHUMG00(	0.7313371	4.338753	6.3675293	2.70E-08	6.61E-07	8.7813926
HSPG2	0.731963	6.4872107	5.2602399	1.92E-06	2.24E-05	4.6445152
TTC14	0.7323934	7.6208234	8.7304337	2.28E-12	3.47E-10	17.936177
DOCK11	0.7331452	7.0961577	2.8637731	0.0057164	0.0179502	-2.934318
RNY4P17	0.7333117	5.4646421	5.0268754	4.58E-06	4.65E-05	3.8062401
LPAR1	0.7335525	5.284661	4.6180633	2.02E-05	0.0001614	2.376886
ADAMTS16	0.7343773	5.4713693	4.4900612	3.19E-05	0.0002369	1.9409915
PALMD	0.7347461	8.3197667	4.6648263	1.71E-05	0.0001406	2.5375971
MIR548Z	0.7349845	5.058932	6.6592246	8.56E-09	2.52E-07	9.9009909
NPIPL2	0.7352068	9.8276649	8.1184528	2.58E-11	2.25E-09	15.565998
OTTHUMG00(	0.7355181	4.0777231	5.3706985	1.27E-06	1.58E-05	5.0462377
MGC24103	0.7356567	4.5493348	4.8450453	8.91E-06	8.20E-05	3.1638962
LOC644450	0.7364991	6.45425	4.2994351	6.21E-05	0.0004119	1.3033555

TAB3-AS1	0.73712	6.2215503	6.7108507	6.98E-09	2.12E-07	10.09996
CAV1	0.7377291	6.0764982	4.2848435	6.53E-05	0.0004302	1.2551433
MIR1290	0.7381362	7.2313918	6.0396649	9.74E-08	1.89E-06	7.5347101
MMP14	0.7382563	7.5396066	3.1041089	0.0028822	0.0101729	-2.306625
MSR1	0.738257	4.858758	4.7030744	1.49E-05	0.0001253	2.669609
OTTHUMG000	0.7388247	4.0921946	5.254837	1.96E-06	2.28E-05	4.6249436
OTTHUMG000	0.7418072	4.9132557	3.5586329	0.0007247	0.0032017	-1.022399
RNY4P24	0.7419703	5.8006789	4.4678307	3.45E-05	0.000253	1.8659056
OTTHUMG000	0.7430923	11.69597	8.1955988	1.90E-11	1.76E-09	15.865632
RC3H1-IT1	0.7434396	5.6585864	6.6512843	8.84E-09	2.58E-07	9.8704083
ARGLU1	0.7446276	10.854994	6.9590253	2.61E-09	9.26E-08	11.059143
SNORD114-1	0.7456787	3.6941269	2.5593929	0.0129562	0.0352966	-3.673114
MS4A7	0.7473289	7.1551066	2.6454782	0.010339	0.0293307	-3.470798
MECOM	0.7476264	7.5158915	4.7146495	1.43E-05	0.0001213	2.7096581
PXDN	0.7488324	7.2408961	4.8919494	7.51E-06	7.06E-05	3.3286262
IGHV3OR16-	0.7489436	3.8191798	3.194894	0.0022066	0.0081603	-2.05993
OTTHUMG000	0.7495403	5.3568064	6.5694163	1.22E-08	3.38E-07	9.5554068
ABCA6	0.7520919	4.6362554	3.9341294	0.0002147	0.0011579	0.1239414
PDE5A	0.7529576	5.8178238	6.4555869	1.91E-08	4.94E-07	9.1184763
RNU6-35P	0.7534568	8.1956748	6.0462019	9.49E-08	1.85E-06	7.5594216
DGKH	0.7538971	6.0659548	11.117698	2.33E-16	3.00E-13	26.893945
MCAM	0.7546461	6.1355002	4.6235307	1.98E-05	0.0001589	2.3956362
SLC38A1	0.7553136	7.0390638	2.842226	0.0060685	0.01883	-2.988731
NBPF8P	0.7557464	9.1698902	10.5191	2.21E-15	1.80E-12	24.704127
TAGLN	0.7566254	6.90449	3.640351	0.0005592	0.0025755	-0.779102
SNORD109B	0.7570454	3.9979718	3.7208758	0.0004318	0.0020807	-0.53591
OTTHUMG000	0.7577271	5.7705205	5.100426	3.48E-06	3.70E-05	4.0688356
IL33	0.7593626	4.7873228	5.2744126	1.82E-06	2.15E-05	4.69589
MIR1285-1	0.7607617	8.2915707	7.6299348	1.81E-10	1.06E-08	13.665801
RARRES1	0.7609392	4.5810639	2.9976617	0.0039193	0.0131004	-2.589271
RNA5SP437	0.7614455	4.8113467	6.3356845	3.06E-08	7.31E-07	8.6597049
OTTHUMG000	0.7615379	4.5678498	5.134507	3.07E-06	3.32E-05	4.1910271
HPGDS	0.7624067	3.8677033	4.2033061	8.65E-05	0.0005449	0.9873617
MIR3140	0.7632171	4.258959	4.0009009	0.0001719	0.0009662	0.3350568
WSB1	0.7634196	12.038113	5.7699316	2.77E-07	4.46E-06	6.5210873
OTTHUMG000	0.7659188	6.2759864	7.4882733	3.18E-10	1.67E-08	13.11455
TES	0.7674625	7.287713	5.7488801	3.00E-07	4.77E-06	6.4425112
OTTHUMG000	0.7675824	4.669909	4.8053674	1.03E-05	9.22E-05	3.0250898
FILIP1L	0.7676835	4.9760385	5.9897851	1.18E-07	2.22E-06	7.3463689
MIR4516	0.7679953	8.8796775	7.9846241	4.40E-11	3.37E-09	15.045821
TLR7	0.7681982	4.7776225	3.2060551	0.0021347	0.0079465	-2.029251
MIR335	0.7690671	4.2007539	5.472178	8.63E-07	1.15E-05	5.417872
KPNA2	0.7694532	9.576141	5.3379882	1.43E-06	1.76E-05	4.9269635
BHLHE41	0.7697804	6.8079279	3.570307	0.0006985	0.0031072	-0.987862
MIR4296	0.7700561	6.6522772	6.8295643	4.36E-09	1.43E-07	10.558265
OTTHUMG000	0.7703233	3.9169043	6.080999	8.29E-08	1.65E-06	7.6910696
SLFN5	0.770626	10.767895	4.9424068	6.24E-06	6.03E-05	3.5065963
RNA5SP435	0.7728574	4.6314995	5.9627094	1.31E-07	2.40E-06	7.244296
PIK3R3	0.7736475	8.754387	7.1315512	1.31E-09	5.35E-08	11.728062
OTTHUMG000	0.7742153	8.1214352	6.429428	2.12E-08	5.37E-07	9.0182522
CLEC2D	0.774463	4.8795697	6.0601361	8.99E-08	1.77E-06	7.6121175

SMYD3-IT1	0.7749202	7.1764485	7.8518393	7.46E-11	5.31E-09	14.529335
RCN1P2	0.7751134	11.510528	6.4132874	2.26E-08	5.65E-07	8.9564483
RNU4-9P	0.7751978	5.0099236	3.6827689	0.0004882	0.0023063	-0.651416
DCDC2	0.7752191	10.013025	3.2746149	0.0017389	0.0066939	-1.839141
JAG1	0.776215	7.0851513	4.7299413	1.35E-05	0.0001157	2.7626369
ABCA9-AS1	0.7768422	4.0819889	7.5365344	2.62E-10	1.44E-08	13.302331
MICAL2	0.7769696	7.5853715	4.636938	1.89E-05	0.0001527	2.4416605
RNU7-41P	0.7775853	5.6239938	5.5984133	5.34E-07	7.68E-06	5.8833711
OTTHUMG00(	0.77818	4.3118295	4.7520069	1.25E-05	0.000108	2.8392217
CPA4	0.7794174	3.9758557	4.4773238	3.33E-05	0.000246	1.8979468
MIR365A	0.7814808	5.6791751	7.1356207	1.29E-09	5.31E-08	11.743857
LOC643648	0.7827084	5.5903582	5.6724053	4.02E-07	6.09E-06	6.1577707
OTTHUMG00(	0.7827209	4.5547851	5.9707758	1.27E-07	2.35E-06	7.2746935
ITIH2	0.7835344	4.9175989	3.657413	0.0005295	0.0024616	-0.727856
OTTHUMG00(	0.7856121	7.4077374	4.4842926	3.25E-05	0.000241	1.9214895
OTTHUMG00(	0.7856537	7.1203523	8.6395489	3.27E-12	4.59E-10	17.58534
PIEZO2	0.785744	6.862757	5.2291888	2.16E-06	2.47E-05	4.5321367
LIX1	0.7865883	5.5665915	3.4649274	0.0009716	0.0041066	-1.296918
ELMO1-AS1	0.7882891	4.6533607	6.7362405	6.31E-09	1.95E-07	10.197892
ULK4P1	0.7888324	5.6111693	6.3389088	3.02E-08	7.25E-07	8.6720206
OTTHUMG00(	0.7893767	6.0674807	4.7145187	1.43E-05	0.0001213	2.7092056
RNU6-28P	0.7903785	7.340608	4.9497957	6.08E-06	5.91E-05	3.5327232
RAPGEF5	0.7907814	6.2426923	7.2373421	8.63E-10	3.79E-08	12.138866
PCCA-AS1	0.7917587	4.5810387	8.4005402	8.42E-12	9.36E-10	16.660597
OTTHUMG00(	0.7931698	5.0959136	5.2834583	1.76E-06	2.09E-05	4.7287064
OTTHUMG00(	0.7962135	4.4975549	5.2495428	2.00E-06	2.32E-05	4.6057729
OTTHUMG00(	0.7966592	4.4741105	6.4328888	2.09E-08	5.31E-07	9.0315077
OTTHUMG00(	0.7993711	4.8582982	4.4827403	3.27E-05	0.0002422	1.9162436
FAR1-IT1	0.8006782	5.7896762	7.7631455	1.06E-10	7.06E-09	14.184218
MIR3173	0.8009858	5.534609	4.1878038	9.12E-05	0.0005696	0.9367666
OTTHUMG00(	0.8012192	4.1381789	4.3399969	5.40E-05	0.0003663	1.4378307
MPEG1	0.8026349	7.7157034	2.5499987	0.0132755	0.03604	-3.694867
IGHV1-69	0.8027575	4.3461028	2.6027505	0.0115711	0.0321124	-3.571884
OTTHUMG00(	0.8033227	4.1064992	5.3234207	1.51E-06	1.84E-05	4.8739279
OSMR	0.8033843	8.2523146	5.4547693	9.22E-07	1.22E-05	5.3539506
MIR548F2	0.8046341	7.0054202	3.3903524	0.0012232	0.0049918	-1.511881
MN1	0.805573	5.7446482	7.3922881	4.66E-10	2.27E-08	12.741175
RNU7-20P	0.80564	5.6251477	7.373755	5.01E-10	2.39E-08	12.669102
OTTHUMG00(	0.806725	4.7728695	4.1064446	0.0001204	0.00072	0.6729353
OTTHUMG00(	0.8072502	8.561501	5.5211887	7.16E-07	9.87E-06	5.5981928
IGHV6-1	0.8080332	6.5137633	2.9240925	0.0048288	0.0155808	-2.780335
MS4A4E	0.808568	4.3476172	4.857833	8.51E-06	7.87E-05	3.2087388
CRIP1P4	0.8086744	8.3078438	3.019151	0.0036854	0.012421	-2.532796
GXYLT2	0.811406	5.9721467	3.9339552	0.0002149	0.0011583	0.1233932
OTTHUMG00(	0.8123345	4.7431898	7.3396053	5.74E-10	2.68E-08	12.536317
RELN	0.8148817	5.2560879	3.5673503	0.000705	0.0031323	-0.996616
ACTN1	0.8150008	6.8615549	4.3169773	5.84E-05	0.0003922	1.3614315
OTTHUMG00(	0.8156005	8.7910395	4.918053	6.83E-06	6.50E-05	3.4205999
ACTA2	0.8158646	8.0367985	3.6171322	0.0006021	0.0027451	-0.848594
HTR2B	0.8163546	7.3346379	3.4584976	0.0009912	0.0041763	-1.315575
OTTHUMG00(	0.8164036	4.0376118	9.2147898	3.39E-13	7.76E-11	19.796741

OTTHUMG00(	0.8189821	3.5204969	6.3069026	3.43E-08	8.00E-07	8.5498237
PRSS23	0.8192573	6.9498574	7.3337946	5.88E-10	2.72E-08	12.513726
CD163	0.8194201	5.4465533	2.5694483	0.0126222	0.0345396	-3.649758
TOP2A	0.8195346	4.4782148	4.6182361	2.02E-05	0.0001613	2.3774782
OTTHUMG00(	0.8197431	9.0181864	9.1681364	4.07E-13	8.86E-11	19.618277
COL1A1	0.8202243	4.6406808	5.1738422	2.65E-06	2.95E-05	4.3324502
HGF	0.8205649	4.4935657	4.2367316	7.71E-05	0.0004943	1.0968
COL12A1	0.8206007	7.6278738	3.9843127	0.0001817	0.0010107	0.2824149
OTTHUMG00(	0.8207223	4.858399	8.0415283	3.51E-11	2.82E-09	15.267055
CCNA2	0.8213064	4.5866926	6.4482692	1.97E-08	5.06E-07	9.0904321
NEXN	0.8218485	6.463031	5.5508983	6.40E-07	8.97E-06	5.7077557
LINC00152	0.824822	6.4836646	7.0156571	2.08E-09	7.72E-08	11.278555
FABP5	0.825447	7.0025202	4.4306784	3.93E-05	0.0002819	1.7408395
MIR548M	0.8258002	5.4088395	4.7209588	1.40E-05	0.000119	2.7315074
BTLA	0.8299895	4.6760818	5.3152101	1.56E-06	1.89E-05	4.8440588
SNORA37	0.8302974	4.3550272	5.9214502	1.54E-07	2.75E-06	7.0889802
WWC3-AS1	0.831928	4.5057987	4.7970605	1.06E-05	9.43E-05	2.9960934
OTTHUMG00(	0.8326841	7.1888164	7.4113064	4.32E-10	2.14E-08	12.815142
AGAP4	0.832783	8.8560444	8.0025337	4.09E-11	3.18E-09	15.115458
MIR4323	0.8328692	8.8749693	8.0588613	3.27E-11	2.69E-09	15.334428
OTTHUMG00(	0.8337169	5.6335456	4.7478233	1.27E-05	0.0001094	2.8246891
OTTHUMG00(	0.8337441	4.2130769	5.0482065	4.23E-06	4.35E-05	3.8822396
SNORD114-3	0.8341577	3.4818459	3.3808753	0.0012593	0.0051168	-1.538972
OTTHUMG00(	0.8343049	6.7735628	4.1944292	8.92E-05	0.0005585	0.9583777
RNY1P4	0.8362761	4.7520352	4.2216171	8.13E-05	0.0005164	1.0472552
RNU7-16P	0.8381291	5.4265987	5.1309951	3.11E-06	3.36E-05	4.1784213
IGHV3-66	0.8397297	4.0980625	2.8194873	0.0064616	0.0198334	-3.045812
RNU6-24	0.8402321	9.6076116	5.5208263	7.17E-07	9.87E-06	5.5968574
NR2F2-AS1	0.8410914	5.4140482	6.348546	2.91E-08	7.05E-07	8.708838
OTTHUMG00(	0.8412534	8.2882351	9.1065149	5.19E-13	1.05E-10	19.382294
OTTHUMG00(	0.8417436	4.5623426	4.3569142	5.09E-05	0.0003488	1.4941124
NKTR	0.8439046	7.0463946	8.2771305	1.37E-11	1.35E-09	16.182086
CFLAR-AS1	0.8449513	5.0245856	7.7947624	9.37E-11	6.33E-09	14.307251
RNA5SP493	0.844982	5.3644848	5.717559	3.38E-07	5.27E-06	6.325757
NPIPA3	0.849135	12.508295	10.055316	1.30E-14	6.86E-12	22.978965
NCKAP1L	0.8500871	6.552188	3.2174604	0.0020634	0.0077251	-1.997822
OTTHUMG00(	0.8506402	5.2432748	7.4010399	4.50E-10	2.20E-08	12.775212
TSHZ2	0.8509857	6.583942	4.7057554	1.48E-05	0.0001244	2.6788811
OTTHUMG00(	0.851151	4.8665836	4.7623752	1.20E-05	0.0001046	2.8752637
OTTHUMG00(	0.8513587	6.8555682	3.8891101	0.0002493	0.0013113	-0.017211
TM6SF1	0.8519115	5.8520333	5.8926293	1.72E-07	3.03E-06	6.9806534
PSMD6-AS2	0.8528451	6.5652452	6.2269982	4.69E-08	1.04E-06	8.2453035
IFI44	0.8548312	8.8794677	4.7682044	1.18E-05	0.0001028	2.895543
MIR548H4	0.8552334	4.359249	6.3370649	3.05E-08	7.28E-07	8.6649773
SNORD14A	0.8552672	5.3955962	3.6732875	0.0005033	0.0023624	-0.680039
MIR181B1	0.8555194	4.5387343	3.2605088	0.0018142	0.0069322	-1.878487
LOC100505§	0.8556697	6.6694648	5.8157789	2.32E-07	3.86E-06	6.6924966
NPIPB6	0.856609	7.8312336	9.18488	3.81E-13	8.48E-11	19.682347
MYOF	0.8570062	9.8565566	4.4361956	3.85E-05	0.0002774	1.7593785
OTTHUMG00(	0.8573933	4.0079757	4.6660982	1.70E-05	0.0001401	2.5419787
MIR548AL	0.8586548	7.2771802	5.2869317	1.74E-06	2.07E-05	4.7413127

PAG1	0.8596644	5.8302452	6.4649674	1.84E-08	4.80E-07	9.1544338
IGHV3-35	0.8608178	3.7887844	2.6536318	0.010118	0.0288014	-3.451359
VCAM1	0.8612474	12.264196	2.5164494	0.0144747	0.038637	-3.772025
TMEM14E	0.8613505	4.653438	6.0030087	1.12E-07	2.12E-06	7.3962624
ULK4P3	0.8632193	5.8104846	5.7203407	3.35E-07	5.23E-06	6.3361187
FGF7	0.863898	4.6101352	3.3583614	0.0013491	0.0054287	-1.603121
TMEM130	0.8639225	9.3195887	3.1343748	0.002638	0.0094567	-2.224951
OTTHUMG00(	0.8640594	7.3662977	5.574578	5.85E-07	8.30E-06	5.7952166
RNA5SP157	0.8657183	3.8381643	8.0598926	3.26E-11	2.69E-09	15.338436
GPR52	0.8665657	4.7206628	7.0423825	1.87E-09	7.11E-08	11.382156
OTTHUMG00(	0.8667157	5.6111611	8.0141748	3.91E-11	3.07E-09	15.160718
IGKV2-29	0.8675939	3.2622459	2.7283697	0.0082839	0.0243636	-3.270987
PP12708	0.8680941	4.4211436	5.5519796	6.37E-07	8.94E-06	5.711747
LAMA4	0.868394	5.6008916	5.3624743	1.31E-06	1.63E-05	5.016225
BMP5	0.8704115	4.5488111	4.0338894	0.0001538	0.0008834	0.4401211
HLA-DQB1-A	0.8722676	7.0135052	2.6434544	0.0103946	0.0294423	-3.475615
IGKV3D-11	0.8749746	5.1937102	2.7610558	0.0075819	0.0226571	-3.190871
CDC42-IT1	0.8765982	4.554462	7.5040018	2.98E-10	1.60E-08	13.175746
MIR590	0.8777974	4.3557734	4.6842177	1.60E-05	0.0001329	2.6044628
LAMP5	0.879745	5.985728	2.774063	0.0073181	0.0219878	-3.158783
MIR571	0.8810362	5.6560964	6.4244254	2.16E-08	5.45E-07	8.9990939
RNU7-76P	0.8822059	3.9338156	8.7010093	2.56E-12	3.79E-10	17.822644
SCARNA3	0.8823529	6.3413074	4.7390657	1.31E-05	0.0001125	2.7942861
LOC399753	0.8835318	10.394764	6.2991482	3.53E-08	8.20E-07	8.5202368
OTTHUMG00(	0.8836443	6.3665374	7.9328726	5.40E-11	4.02E-09	14.844562
CTSK	0.8842688	6.0718646	5.5718673	5.91E-07	8.37E-06	5.7851987
MIR29B2	0.884845	5.6691969	8.3700378	9.50E-12	1.02E-09	16.542386
OTTHUMG00(	0.8850981	4.8866615	5.8432479	2.08E-07	3.53E-06	6.7953758
RNA5SP53	0.8862257	4.6590374	3.6081971	0.0006195	0.0028109	-0.875259
MIR597	0.886612	4.2525766	5.8823521	1.79E-07	3.13E-06	6.9420591
SOX4	0.886733	7.5216089	4.6377951	1.88E-05	0.0001523	2.444605
RAB3B	0.8885131	4.7220413	3.6436614	0.0005533	0.0025527	-0.769171
LPAR6	0.8885972	6.5027169	10.531595	2.11E-15	1.76E-12	24.75027
TRAJ14	0.8901693	3.928798	3.8013768	0.0003325	0.001672	-0.289466
FTX	0.8924817	7.7979162	6.7360527	6.32E-09	1.95E-07	10.197167
IGHV3-48	0.8928241	4.6588459	3.4317344	0.0010769	0.004478	-1.392987
FMOD	0.8930328	5.7848816	3.8518024	0.0002819	0.0014556	-0.133445
MIR603	0.8934443	5.3807552	6.2321117	4.60E-08	1.02E-06	8.2647673
NPIPA5	0.8948855	12.469475	10.462647	2.74E-15	2.07E-12	24.49542
OTTHUMG00(	0.8955325	4.8970828	6.258268	4.15E-08	9.36E-07	8.3643793
TNFRSF12A	0.8962028	9.6369608	3.388407	0.0012305	0.0050158	-1.517447
AKAP11-IT1	0.8965678	5.1171121	4.6941797	1.54E-05	0.0001289	2.6388642
SNORD114-1	0.8974371	4.479013	3.077857	0.003111	0.0108357	-2.377001
OTTHUMG00(	0.9009618	5.849259	5.860242	1.95E-07	3.34E-06	6.8590898
CP	0.9017809	4.4598575	4.6717197	1.67E-05	0.0001378	2.5613522
MRC1	0.9031139	6.6786195	2.5065946	0.014845	0.0394519	-3.794532
LINC00685	0.9035384	6.1750913	8.5340659	4.96E-12	6.36E-10	17.177573
IGHV2-5	0.9043237	6.6936407	2.8754282	0.005534	0.0174713	-2.904755
FLJ45340	0.9049188	10.161113	6.3738884	2.64E-08	6.48E-07	8.8057063
AGAP6	0.9059565	10.189007	6.9682729	2.52E-09	8.99E-08	11.09496
PMP22	0.9069344	9.8307284	3.8514882	0.0002822	0.0014563	-0.134421

OTTHUMG00(	0.9087639	4.7947085	5.5962587	5.38E-07	7.73E-06	5.8753974
CCDC80	0.9097895	8.0316098	3.0111701	0.0037707	0.0126747	-2.553804
MIR580	0.9099267	4.4152166	5.221017	2.22E-06	2.53E-05	4.5026031
OTTHUMG00(	0.9125746	4.3276513	4.550258	2.58E-05	0.000197	2.1452387
RNY5	0.9138094	7.6824233	3.4917672	0.0008937	0.0038224	-1.218784
BACH1-IT2	0.9140565	5.1738813	6.2448991	4.37E-08	9.81E-07	8.313455
FCER1A	0.9146404	4.9610323	3.0668723	0.0032117	0.0111225	-2.40632
RNU7-6P	0.9148626	7.5336916	6.0243257	1.03E-07	1.99E-06	7.4767501
SNORA50	0.9186683	6.6554966	3.6567033	0.0005307	0.0024663	-0.72999
RNU7-69P	0.9195697	7.998323	7.3394328	5.75E-10	2.68E-08	12.535647
OTTHUMG00(	0.920369	8.2085407	4.3947472	4.46E-05	0.0003119	1.62039
OTTHUMG00(	0.9218263	7.5525502	9.4634298	1.28E-13	3.48E-11	20.74485
CD93	0.9220135	7.7417705	4.6338031	1.91E-05	0.000154	2.4308936
RNU7-57P	0.9228215	9.2378846	6.5420262	1.36E-08	3.69E-07	9.4501553
ARL4C	0.9230357	4.8834756	3.8391446	0.0002938	0.0015075	-0.172727
OTTHUMG00(	0.9240106	6.5254528	4.2806467	6.63E-05	0.0004353	1.2412931
OTTHUMG00(	0.9241749	4.8239177	5.6250787	4.82E-07	7.06E-06	5.9821322
RNU6-73	0.9255775	7.166892	6.2657811	4.03E-08	9.15E-07	8.3930074
ANXA1	0.925635	11.790655	7.0575481	1.76E-09	6.78E-08	11.440961
MIR320D1	0.9257225	5.5864934	3.9347743	0.0002143	0.0011562	0.1259703
RNU6-44	0.9268242	5.7566339	6.8083527	4.75E-09	1.54E-07	10.476301
AGAP7	0.9269024	10.329442	6.8704198	3.71E-09	1.25E-07	10.716219
MIR4531	0.9276766	5.6587808	4.3482233	5.24E-05	0.0003574	1.4651845
LOC728734	0.9290182	8.6704741	9.5473642	9.26E-14	2.73E-11	21.063697
CTSC	0.9301722	10.534289	3.1196206	0.0027545	0.0098119	-2.264838
RNA5SP390	0.930821	4.7999228	4.7186115	1.41E-05	0.0001198	2.7233771
PCDH17	0.9325331	7.3095956	4.6854205	1.59E-05	0.0001325	2.6086145
MIR4295	0.9331786	4.2175854	3.9253934	0.0002211	0.0011841	0.0964751
MIR553	0.935361	4.8009252	3.8006003	0.0003334	0.001676	-0.291859
SNORD69	0.9360854	6.7690321	2.9356982	0.0046734	0.0151713	-2.75043
RNU7-23P	0.9370057	4.5976767	6.4746357	1.77E-08	4.64E-07	9.1915041
SAMHD1	0.9373725	9.2527528	4.1240057	0.0001134	0.0006848	0.7296378
RNA5SP212	0.9390501	4.6056418	4.7300343	1.35E-05	0.0001157	2.7629594
RNU6-43	0.9397468	8.5973461	6.7141875	6.89E-09	2.10E-07	10.112828
MIR99A	0.9415215	3.5900425	4.6792131	1.62E-05	0.0001345	2.5871936
CD1C	0.9435298	4.7252143	3.6490804	0.0005438	0.0025156	-0.752902
COL10A1	0.9474516	4.6112967	6.4177497	2.22E-08	5.57E-07	8.973532
NAV2-IT1	0.9485446	3.9872069	5.345454	1.39E-06	1.72E-05	4.9541638
LOC100506(	0.9506634	4.5952505	8.2831372	1.34E-11	1.33E-09	16.20539
RNA5SP68	0.9512577	4.4874044	4.8019433	1.04E-05	9.31E-05	3.0131348
LOC101060(	0.951865	8.0683751	9.8407711	2.97E-14	1.11E-11	22.173122
KCNQ1OT1	0.9544883	5.3849403	5.2552708	1.96E-06	2.27E-05	4.6265146
SNORD93	0.9554575	3.9776043	4.4721769	3.40E-05	0.0002496	1.8805705
MIR548H1	0.9560855	5.5565374	4.1080361	0.0001198	0.0007166	0.6780684
HAVCR1P1	0.9576817	5.6360987	5.3787808	1.23E-06	1.54E-05	5.0757487
LINC00597	0.9593336	4.5510095	5.7181641	3.37E-07	5.26E-06	6.3280106
GFRA1	0.9602441	5.5635034	4.7416962	1.30E-05	0.0001116	2.8034156
SNORD4B	0.9602998	6.1377869	3.6389866	0.0005616	0.0025841	-0.783193
RNA5SP123	0.9607037	5.3489074	6.1620877	6.04E-08	1.27E-06	7.9985322
IGHV3-33	0.9636576	6.4234428	2.991529	0.0039886	0.0132863	-2.605333
DOCK9	0.9642356	8.2015838	8.2904798	1.30E-11	1.30E-09	16.233876

OTTHUMG00(	0.9647436	4.9094748	6.8175722	4.58E-09	1.50E-07	10.511922
OTTHUMG00(	0.965203	7.6507505	4.3209	5.77E-05	0.0003878	1.3744354
ENAH	0.9664034	7.6953708	5.8612938	1.94E-07	3.34E-06	6.8630346
C10orf113	0.9667849	5.0514057	4.602194	2.14E-05	0.0001693	2.3225228
KRT19	0.9703748	6.9077726	2.855498	0.0058493	0.0182748	-2.955252
MIR558	0.9718076	4.6223587	5.1319693	3.10E-06	3.35E-05	4.1819177
RNU6-45P	0.977637	10.307472	5.3900508	1.18E-06	1.49E-05	5.1169243
PRELP	0.9792825	7.8250057	4.0839926	0.0001299	0.0007665	0.6006397
MIR30C2	0.9803207	6.4030256	4.679331	1.62E-05	0.0001345	2.5876001
RNU1-16P	0.9808259	5.9071502	5.6731832	4.01E-07	6.08E-06	6.1606614
RNU7-25P	0.981742	7.1099174	7.8645101	7.09E-11	5.10E-09	14.578632
SCARNA2	0.9842699	8.4749244	3.7901791	0.0003449	0.0017247	-0.323942
PROM1	0.9855616	7.2795916	4.0133881	0.0001648	0.0009336	0.3747683
MIR21	0.9856985	5.8534882	3.0053905	0.0038336	0.012853	-2.568993
OTTHUMG00(	0.9863106	4.7416161	6.6691057	8.23E-09	2.44E-07	9.9390562
PPP1R3C	0.9872735	10.622293	3.4532912	0.0010073	0.0042344	-1.330666
SNRK-AS1	0.9881809	7.1665397	8.1049333	2.72E-11	2.34E-09	15.513471
ANKRD44-I1	0.9890994	5.6298075	6.020501	1.05E-07	2.01E-06	7.4623039
RNU7-11P	0.9920138	7.1144774	7.8005751	9.15E-11	6.26E-09	14.32987
PLN	0.9937963	8.3731344	3.2637737	0.0017965	0.0068799	-1.869391
LINC00924	0.994116	5.3959408	4.6295301	1.94E-05	0.0001561	2.416223
EDNRB	0.9942949	8.3431451	3.7253888	0.0004256	0.0020544	-0.522181
OTTHUMG00(	0.9964663	4.095368	6.6446088	9.07E-09	2.64E-07	9.8447009
RNU7-28P	0.9969398	6.7507723	6.2952726	3.59E-08	8.29E-07	8.5054519
OTTHUMG00(	0.9973165	5.030163	6.0238606	1.04E-07	1.99E-06	7.4749932
OTTHUMG00(	0.9997699	4.59412	5.8414393	2.10E-07	3.55E-06	6.788598
N4BP2L2-I1	0.9999117	5.7589703	5.8603917	1.95E-07	3.34E-06	6.8596514
LOC642799	1.0007129	12.58098	10.207878	7.24E-15	4.30E-12	23.549069
LOC100996	1.0007765	5.987288	8.2908048	1.30E-11	1.30E-09	16.235137
SFRP4	1.0009526	4.6878949	3.4603293	0.0009856	0.004156	-1.310263
OTTHUMG00(	1.0022485	4.7877925	4.716989	1.42E-05	0.0001204	2.7177586
CCND2	1.0030436	7.0825289	5.6375756	4.60E-07	6.78E-06	6.028467
PDE1A	1.0032764	6.5845908	4.9581415	5.89E-06	5.77E-05	3.5622532
OTTHUMG00(	1.0069267	5.6128303	6.3956558	2.42E-08	6.00E-07	8.8889679
LOC100216	1.007564	4.7933292	7.5768625	2.23E-10	1.25E-08	13.459262
TPM1	1.0138177	10.776374	5.8474586	2.05E-07	3.49E-06	6.8111576
CDH11	1.0145408	6.922192	4.3513814	5.19E-05	0.000354	1.4756929
GUSBP2	1.0168471	8.1867641	7.7505547	1.12E-10	7.39E-09	14.135221
MS4A4A	1.0168581	5.5128895	3.1829724	0.002286	0.0083963	-2.092616
OTTHUMG00(	1.0168902	6.7458836	6.6294773	9.63E-09	2.77E-07	9.786444
SLC12A2	1.0184777	7.4701631	4.8625886	8.36E-06	7.75E-05	3.2254285
CD44	1.0204914	7.1324166	3.4828953	0.0009188	0.0039107	-1.244656
CTHRC1	1.0214427	5.299323	6.489242	1.67E-08	4.42E-07	9.2475258
NDUFB2	1.021586	5.3203543	4.4518344	3.65E-05	0.0002652	1.8119925
RNU6-21P	1.0216686	6.4360823	6.7486487	6.01E-09	1.87E-07	10.24577
RNU6-80	1.02669	6.3417108	7.1285229	1.33E-09	5.40E-08	11.716309
RNA5SP450	1.0298768	7.2766543	5.7254077	3.28E-07	5.15E-06	6.3549966
RNA5SP166	1.0309621	6.2865152	7.2636871	7.77E-10	3.46E-08	12.241229
CD248	1.0324115	11.860546	3.6591077	0.0005267	0.0024536	-0.722757
IGKV3-7	1.0354415	5.2851215	3.4425619	0.0010414	0.0043528	-1.361718
MIR4263	1.0411795	6.4453877	5.0690323	3.91E-06	4.07E-05	3.9565643

ANGPT2	1.0424732	5.7081292	6.5739369	1.20E-08	3.33E-07	9.5727849
BCL6B	1.0451412	7.195693	5.8653617	1.91E-07	3.30E-06	6.8782943
OR51E1	1.0456867	4.8142936	2.7868497	0.007067	0.0213517	-3.127126
ADAMTS5	1.0467926	7.7408549	5.0836121	3.71E-06	3.89E-05	4.0086707
OTTHUMG00(	1.0477009	5.9075349	8.2441784	1.57E-11	1.50E-09	16.054215
TAS2R31	1.0489172	4.9679249	8.0747758	3.07E-11	2.56E-09	15.39628
RNA5SP260	1.0491138	5.076978	5.3460768	1.39E-06	1.71E-05	4.9564335
CLDN1	1.0517342	7.8627023	2.9253094	0.0048123	0.0155373	-2.777204
MIR4275	1.0543479	6.9453056	4.0488001	0.0001463	0.0008466	0.4877729
SYT11	1.0557091	8.1614534	5.0289711	4.54E-06	4.62E-05	3.8137009
IGHV3-21	1.0558556	3.7958854	3.2711962	0.0017569	0.0067549	-1.848687
PLVAP	1.0562009	5.3281885	3.6065614	0.0006227	0.002823	-0.880136
GUCY1A3	1.0564228	8.0512787	7.256649	7.99E-10	3.54E-08	12.21388
SPDYE5	1.0583775	7.1317005	8.2373216	1.61E-11	1.53E-09	16.027602
SNORD99	1.0609082	7.2638634	3.7813292	0.000355	0.001766	-0.351145
IGKV2D-29	1.0632331	4.0668351	2.4773213	0.0159962	0.0419877	-3.860965
GOLGA8B	1.0637445	9.2460418	6.9220003	3.02E-09	1.05E-07	10.915791
RGS4	1.0639086	4.6623416	4.3754209	4.77E-05	0.0003306	1.555813
THBS2	1.0650621	5.1047634	4.5045566	3.03E-05	0.0002267	1.990051
RNU6-79P	1.0681973	5.9565911	3.7137377	0.0004419	0.0021183	-0.557603
SNORA45	1.0686754	6.1412305	4.8114719	1.01E-05	9.06E-05	3.0464124
RNA5SP160	1.0688943	4.1911921	5.321678	1.52E-06	1.85E-05	4.8675867
ANKRD10-I1	1.0697982	6.9771297	4.5562928	2.52E-05	0.000194	2.1657883
RNU7-75P	1.0705569	5.2271116	4.3644187	4.95E-05	0.0003413	1.5191158
OTTHUMG00(	1.0711059	4.4948116	7.3837456	4.82E-10	2.32E-08	12.707953
GPR18	1.0714757	5.3820482	6.9197393	3.05E-09	1.06E-07	10.90704
TGFB2	1.0721081	7.1796733	4.8339341	9.28E-06	8.48E-05	3.1249749
ANKRD36B	1.0745933	7.5876326	8.1808396	2.01E-11	1.85E-09	15.808322
SNORD78	1.0795731	5.7656082	3.3613471	0.0013368	0.00539	-1.594631
NID2	1.0795988	7.8117225	4.941488	6.26E-06	6.05E-05	3.5033489
SNORD75	1.0814321	4.2258767	5.263243	1.90E-06	2.22E-05	4.655397
OTTHUMG00(	1.082307	4.7377933	8.275063	1.39E-11	1.35E-09	16.174064
RNA5SP343	1.0833472	5.9217749	6.5231094	1.47E-08	3.94E-07	9.3775055
OTTHUMG00(	1.0844982	4.4098575	7.0763814	1.64E-09	6.37E-08	11.514003
MIR548C	1.0865273	4.9451789	7.4348625	3.93E-10	2.00E-08	12.906767
RNU7-13P	1.0882431	9.4398951	7.1665854	1.14E-09	4.81E-08	11.86406
MIR30E	1.0891911	3.7447723	5.9217818	1.54E-07	2.75E-06	7.0902273
IGLV6-57	1.0893908	5.1216115	2.8555927	0.0058478	0.0182735	-2.955013
RNA5SP268	1.0934069	5.9198984	4.9179402	6.83E-06	6.50E-05	3.4202021
OTTHUMG00(	1.0935123	5.9846587	7.6332313	1.78E-10	1.05E-08	13.67863
IGKV1-5	1.0950277	6.0592062	2.8195172	0.0064611	0.0198334	-3.045737
LOC645638	1.095928	5.1083203	5.1904508	2.49E-06	2.80E-05	4.3922882
OTTHUMG00(	1.1031506	6.6143089	7.3212847	6.18E-10	2.84E-08	12.465092
DAPL1	1.1034389	9.0204821	3.291242	0.0016539	0.0064262	-1.79261
LOC100272	1.1035184	5.9218536	7.697578	1.38E-10	8.60E-09	13.929053
RNU7-62P	1.1041869	6.9842239	6.9506759	2.70E-09	9.54E-08	11.026809
RNU7-47P	1.1049719	9.03495	8.198745	1.88E-11	1.75E-09	15.877848
F2RL2	1.1097247	6.8516895	4.6813884	1.61E-05	0.0001339	2.5946989
SPDYE8P	1.1116729	6.6171725	9.2773376	2.66E-13	6.36E-11	20.035733
LOC100131	1.1120111	4.8941603	3.7714376	0.0003666	0.0018144	-0.381503
PLK2	1.1129914	7.8192672	6.125562	6.97E-08	1.43E-06	7.8599231

GOLGA8A	1.1164905	9.1887766	7.6109079	1.95E-10	1.12E-08	13.591753
COL15A1	1.1168892	5.3096185	5.7440367	3.05E-07	4.84E-06	6.4244446
OTTHUMG000	1.1171202	8.7265928	3.0955546	0.002955	0.0103894	-2.329605
PCDH18	1.1200069	6.096419	5.666594	4.11E-07	6.18E-06	6.1361798
RASSF9	1.1239346	5.3406825	5.1881899	2.51E-06	2.81E-05	4.3841383
LOC1002874	1.1249232	6.6324854	5.9670786	1.29E-07	2.38E-06	7.2607596
RNU7-24P	1.1283138	8.7809593	7.401389	4.49E-10	2.20E-08	12.77657
RNY3P6	1.128641	7.4353348	4.6850167	1.59E-05	0.0001326	2.6072207
IGFBP6	1.1297055	6.7663452	5.0141798	4.79E-06	4.84E-05	3.7610697
CACNA1C-AS	1.132398	5.339771	7.3125629	6.40E-10	2.93E-08	12.431188
TAS2R30	1.1332511	5.0183179	6.998531	2.23E-09	8.16E-08	11.212184
KLF7-IT1	1.1340588	7.2517457	6.0575133	9.08E-08	1.79E-06	7.6021965
OTTHUMG000	1.1404466	6.2090772	6.8675504	3.75E-09	1.26E-07	10.705123
MIR1285-2	1.1405463	11.831797	8.7341849	2.25E-12	3.46E-10	17.950647
MIR3671	1.1417398	7.2574164	6.5151556	1.51E-08	4.05E-07	9.3469693
PCDHGA10	1.1426307	8.9030507	7.6511012	1.66E-10	9.92E-09	13.748175
CFH	1.1430475	11.514882	3.6250896	0.0005871	0.0026843	-0.82481
LTBP1	1.1443192	7.5912551	6.2828342	3.77E-08	8.63E-07	8.458014
ANKRD36	1.1452484	8.0171221	8.2439103	1.57E-11	1.50E-09	16.053175
MIR186	1.1460906	6.6217082	5.9834847	1.21E-07	2.26E-06	7.3226067
OTTHUMG000	1.1503292	5.4051041	5.5460493	6.52E-07	9.10E-06	5.6898605
RNU6-82P	1.1513573	5.1753631	5.5983102	5.34E-07	7.68E-06	5.8829898
LOC1009968	1.1524086	8.3159757	7.7671474	1.05E-10	6.96E-09	14.199792
IGKV1-9	1.1543662	6.052932	3.0197407	0.0036792	0.0124077	-2.531241
OLFML2B	1.1558523	5.4172084	4.1877277	9.13E-05	0.0005696	0.9365185
SVEP1	1.1571432	4.494811	4.4042871	4.31E-05	0.0003036	1.6523209
PER3	1.158347	7.6431936	7.081244	1.61E-09	6.27E-08	11.532865
IGKV1-17	1.1601461	5.9857418	2.8621646	0.005742	0.0180069	-2.93839
ITGB6	1.1612974	5.4370807	3.4682768	0.0009616	0.0040691	-1.287189
IGKV1D-42	1.1623446	6.3415585	2.8632652	0.0057245	0.0179701	-2.935604
ZNF638-IT1	1.1633458	6.7222125	6.1391991	6.61E-08	1.37E-06	7.911652
NAIP	1.1638922	6.7814272	7.6434531	1.71E-10	1.02E-08	13.718411
RNU6-83P	1.1645362	5.1163956	8.1402433	2.37E-11	2.11E-09	15.650651
SPDYE1	1.1651719	6.9956498	8.787164	1.82E-12	2.95E-10	18.154919
COL14A1	1.1653475	5.6667908	4.1614077	9.99E-05	0.0006144	0.8508546
CPA3	1.1710426	4.3032067	3.6898803	0.0004772	0.0022639	-0.629918
OTTHUMG000	1.1711454	5.4313508	6.7771139	5.37E-09	1.71E-07	10.355648
RNU7-35P	1.1724397	7.9933672	7.7346412	1.19E-10	7.73E-09	14.073292
ADAMTS1	1.1734314	8.7987643	5.6719642	4.03E-07	6.10E-06	6.1561316
SPDYE3	1.1738299	6.5916821	9.9817584	1.72E-14	7.67E-12	22.703212
MIR548I4	1.17587	14.797559	3.8263074	0.0003065	0.00156	-0.212485
LRP2BP	1.1792319	6.850052	6.6850597	7.73E-09	2.32E-07	10.000533
COL3A1	1.1794649	6.1016661	4.0067272	0.0001685	0.0009514	0.3535765
TGFBI	1.1822861	5.7970508	5.8161852	2.31E-07	3.86E-06	6.6940175
OTTHUMG000	1.186176	6.9496603	6.1537877	6.24E-08	1.31E-06	7.9670186
RNU6-3	1.187658	10.202505	7.2047213	9.83E-10	4.22E-08	12.012151
MIR548AC	1.1909628	15.475708	7.4120883	4.30E-10	2.14E-08	12.818183
OTTHUMG000	1.1962739	5.4888215	6.4424742	2.01E-08	5.15E-07	9.0682277
MIR3120	1.2009898	5.7680511	6.1687223	5.89E-08	1.25E-06	8.023729
NPIPB3	1.2024434	14.63052	9.4247838	1.49E-13	3.98E-11	20.597832
RNU6-60	1.2032495	7.74416	6.148398	6.37E-08	1.33E-06	7.9465602

SYTL2	1.2069115	7.5935223	4.5932377	2.21E-05	0.0001736	2.2918811
OTTHUMG00(	1.2071168	6.5579513	4.8971471	7.37E-06	6.96E-05	3.3469232
OTTHUMG00(	1.2097499	4.7096266	6.3203105	3.25E-08	7.68E-07	8.6009989
IGHJ1	1.2101364	4.6817174	5.8171564	2.31E-07	3.85E-06	6.6976525
MIR570	1.2113674	5.9304633	6.1659997	5.95E-08	1.26E-06	8.0133884
LOC613037	1.215051	14.480484	9.186535	3.79E-13	8.48E-11	19.688678
FBN1	1.2190779	6.6093072	6.0293988	1.01E-07	1.96E-06	7.4959153
SNORD63	1.2205566	6.8197951	4.8975307	7.36E-06	6.95E-05	3.348274
POSTN	1.222471	11.789412	4.0209457	0.0001607	0.0009139	0.3988376
CCL11	1.2231401	5.4081146	3.8637352	0.000271	0.0014068	-0.096341
OTTHUMG00(	1.2239385	6.4763572	4.7930698	1.08E-05	9.54E-05	2.9821715
MIR1299	1.2286047	9.237212	6.2235496	4.75E-08	1.05E-06	8.2321791
LOC1003037	1.231825	5.4438239	6.2844615	3.74E-08	8.60E-07	8.4642191
SMA4	1.2340179	8.0511893	7.1737586	1.11E-09	4.71E-08	11.891911
OTTHUMG00(	1.234234	7.5391946	6.5776515	1.18E-08	3.29E-07	9.587066
NT5E	1.2346646	9.1798395	4.9096922	7.04E-06	6.68E-05	3.3911188
FMO3	1.2369025	5.8459092	4.0435924	0.0001489	0.0008591	0.4711186
NPIPL3	1.23716	12.902574	9.103265	5.25E-13	1.05E-10	19.369841
OTTHUMG00(	1.2373423	8.8300508	7.0190415	2.06E-09	7.65E-08	11.291673
OTTHUMG00(	1.2382908	4.3992813	6.4214606	2.19E-08	5.50E-07	8.9877406
NPIPB5	1.2389362	14.547143	9.6710243	5.73E-14	1.84E-11	21.532279
ATP13A3	1.2407372	11.263572	7.0464615	1.84E-09	7.04E-08	11.397972
GPR34	1.2417137	5.2842234	4.2118545	8.40E-05	0.0005316	1.015305
OTTHUMG00(	1.244964	7.4099136	8.0952775	2.83E-11	2.41E-09	15.475951
MIR30A	1.2512101	3.4992643	8.329126	1.12E-11	1.15E-09	16.38377
SERPINF1	1.2522421	5.4895664	3.9505335	0.0002033	0.0011082	0.1756135
SLC6A6	1.2621207	8.9844721	7.2119569	9.55E-10	4.13E-08	12.040255
VEGFC	1.2637386	7.3976736	5.9343439	1.47E-07	2.63E-06	7.1374873
VSIG4	1.2643568	5.4234036	4.0425113	0.0001494	0.0008615	0.4676629
MIR548D1	1.2674874	18.589733	9.9798771	1.74E-14	7.67E-12	22.696152
RNU7-61P	1.2699616	6.5251357	7.0195845	2.05E-09	7.64E-08	11.293777
MIR548K	1.2784389	5.1524231	5.8352596	2.15E-07	3.62E-06	6.7654437
LOC100506(	1.2793905	7.3044697	10.053286	1.31E-14	6.86E-12	22.971363
HNRNPU-AS1	1.2799168	8.1058615	7.9880145	4.34E-11	3.35E-09	15.059004
IGKV3-11	1.2822032	6.1230872	2.8625179	0.0057364	0.018002	-2.937496
PRINS	1.2887599	4.7942382	8.9120301	1.11E-12	1.95E-10	18.63566
RPL23AP32	1.2908859	7.7314331	7.048209	1.83E-09	7.02E-08	11.404747
OTTHUMG00(	1.2918914	9.5541239	12.67113	8.27E-19	3.20E-15	32.363139
AEBP1	1.2919694	7.0388174	4.7564876	1.23E-05	0.0001066	2.8547932
OTTHUMG00(	1.2955766	6.3015584	7.6440472	1.71E-10	1.01E-08	13.720723
GUSBP3	1.2956656	7.2666487	8.2695922	1.42E-11	1.37E-09	16.152837
OTTHUMG00(	1.2999351	6.1211279	10.560574	1.89E-15	1.62E-12	24.857224
LOC1005061	1.3019688	7.6441218	7.6306821	1.80E-10	1.06E-08	13.668709
MIR95	1.3025339	4.6323836	5.7386778	3.12E-07	4.93E-06	6.4044602
RNU7-7P	1.3040424	9.4918105	8.115476	2.61E-11	2.27E-09	15.554433
THRB-IT1	1.309312	5.5441069	7.2765585	7.38E-10	3.31E-08	12.291248
RNA5SP217	1.3110121	6.2758356	3.8845235	0.0002531	0.001329	-0.031537
RERG-IT1	1.3195092	4.6787348	6.6511655	8.84E-09	2.58E-07	9.8699507
ABI3BP	1.3213397	8.7080456	3.9531025	0.0002016	0.0011014	0.1837174
LOC440354	1.3220631	10.015421	10.025546	1.46E-14	7.26E-12	22.867433
GUSBP9	1.3347079	7.8818267	7.7248242	1.24E-10	7.92E-09	14.035088

IGHV3-30	1.3348439	5.7742297	2.7788223	0.0072237	0.0217523	-3.147013
RPL36AP33	1.3366894	6.1164336	7.3067944	6.55E-10	2.99E-08	12.408765
SLIT3	1.3374946	8.3022923	6.8963342	3.35E-09	1.14E-07	10.816465
MIR548AJ2	1.3449267	16.536972	9.3202237	2.25E-13	5.55E-11	20.199414
LOC595101	1.3454001	9.9442303	9.9152401	2.23E-14	9.56E-12	22.453366
ABCC9	1.3454009	5.0599503	4.6495128	1.81E-05	0.0001471	2.4848846
LINC00342	1.3530118	7.8726693	8.2819907	1.35E-11	1.33E-09	16.200942
MFAP4	1.3589579	7.4006049	4.6084163	2.09E-05	0.0001663	2.3438277
RNU6-38	1.3597292	8.3373479	8.4079832	8.17E-12	9.22E-10	16.689437
MIR612	1.361839	10.332556	6.0213498	1.05E-07	2.00E-06	7.4655097
IGKV1D-33	1.3619955	5.3261021	3.5909766	0.0006542	0.0029389	-0.926531
RNU7-53P	1.3669418	9.0638497	8.770139	1.95E-12	3.11E-10	18.089294
RNA5SP82	1.3705163	6.3071577	5.6028254	5.25E-07	7.58E-06	5.8997021
SNORD7	1.377933	5.4451697	6.4443941	2.00E-08	5.12E-07	9.0755838
IGKC	1.3804615	6.0810297	3.2617296	0.0018076	0.006912	-1.875086
MIR548H3	1.3832518	9.5077331	7.7324497	1.20E-10	7.78E-09	14.064764
LUST	1.3834852	8.030469	8.3562582	1.00E-11	1.05E-09	16.488969
SPDYE2	1.3856496	7.4424262	8.6823095	2.76E-12	4.02E-10	17.750466
MIR503	1.3897996	4.0877626	7.8217894	8.41E-11	5.83E-09	14.412417
SMA5	1.3917636	9.1331516	8.01813	3.85E-11	3.03E-09	15.176095
ACAP2-IT1	1.3941079	4.4083889	8.4816284	6.10E-12	7.34E-10	16.974651
RNF138P1	1.3963693	5.4954723	6.1246424	6.99E-08	1.43E-06	7.8564358
RNU7-10P	1.3976703	5.9973672	6.5608909	1.26E-08	3.48E-07	9.5226388
MIR421	1.3981842	6.6845716	5.7056424	3.54E-07	5.48E-06	6.2813851
TNC	1.3997174	7.5136821	4.4382218	3.83E-05	0.0002759	1.76619
MS4A6A	1.4020442	6.2706361	3.8992963	0.000241	0.0012733	0.0146418
COL6A3	1.4061813	4.9700562	4.824246	9.61E-06	8.72E-05	3.0910706
MIR548G	1.4120571	16.256548	9.9846215	1.71E-14	7.67E-12	22.713956
LYVE1	1.4165542	6.3864867	3.5293483	0.0007946	0.003465	-1.10871
KIRREL-IT1	1.4209349	6.8815536	8.1467293	2.31E-11	2.08E-09	15.675845
MIR548F1	1.4244816	15.460793	9.7910939	3.60E-14	1.26E-11	21.985868
MMP2	1.4290135	5.971621	5.6072233	5.16E-07	7.47E-06	5.9159846
OTTHUMG00(	1.4295339	7.1621962	6.0937303	7.89E-08	1.59E-06	7.7392805
RNU6-8	1.434984	8.2504125	8.0217933	3.79E-11	3.00E-09	15.190337
RGS5	1.4420048	7.4371984	4.6360834	1.90E-05	0.000153	2.4387251
MIR4454	1.4433672	12.170077	8.6967486	2.60E-12	3.83E-10	17.8062
MIR3916	1.4456828	7.5289649	5.2973839	1.67E-06	2.00E-05	4.7792665
MIR548AI	1.4464124	16.546569	9.477192	1.22E-13	3.38E-11	20.797172
OCLM	1.4480625	5.0833669	7.8267279	8.25E-11	5.73E-09	14.431632
RNA5SP229	1.4490526	4.8938874	7.7972327	9.27E-11	6.30E-09	14.316864
ITGA11	1.4614906	6.4964298	4.6680846	1.69E-05	0.0001394	2.5488232
OTTHUMG00(	1.46406	5.4097523	8.1888791	1.95E-11	1.80E-09	15.83954
RNU7-48P	1.4665153	8.6936746	8.3735491	9.37E-12	1.01E-09	16.555996
NELL1	1.4681447	8.2065323	2.8899305	0.0053144	0.0169061	-2.867842
MIR450B	1.469556	4.8369579	7.7991419	9.20E-11	6.28E-09	14.324293
IGKV3D-15	1.4706214	5.3597839	3.0465272	0.0034062	0.0116449	-2.460419
PTN	1.4765989	7.2849526	4.5728573	2.38E-05	0.0001844	2.2222617
MIR548AD	1.4793178	17.900466	9.5370307	9.64E-14	2.78E-11	21.024477
RNA5SP187	1.4807416	9.2346075	8.1598814	2.19E-11	1.99E-09	15.726929
MIR548Q	1.4914021	12.865333	9.0422439	6.67E-13	1.26E-10	19.135859
CCL2	1.4915324	13.268936	3.9780562	0.0001855	0.0010291	0.262593

ZBTB20-AS2	1.4931004	6.5225648	5.9487235	1.39E-07	2.52E-06	7.1916168
IGKV1-6	1.494061	6.5896549	3.133812	0.0026423	0.0094712	-2.226475
SLC7A5P2	1.5045815	10.346285	10.019015	1.49E-14	7.33E-12	22.84295
MIR548AA2	1.5095685	15.354218	8.7559981	2.06E-12	3.22E-10	18.034773
RNU7-29P	1.5141126	8.0682638	9.0905367	5.52E-13	1.09E-10	19.321058
RNU7-45P	1.5170934	6.6581948	8.436198	7.31E-12	8.52E-10	16.798737
INHBA	1.5249517	5.871408	4.8367247	9.19E-06	8.40E-05	3.1347464
IGJ	1.5305456	7.2464193	2.8213133	0.0064292	0.0197529	-3.041241
MARCKS	1.5315616	9.7594328	4.6086324	2.09E-05	0.0001662	2.3445677
MIR548A2	1.5356677	10.955784	5.7777806	2.68E-07	4.35E-06	6.5504053
SPDYE6	1.5433684	8.156943	8.5327569	4.98E-12	6.36E-10	17.172509
SMG1P1	1.5475648	10.68188	9.8928891	2.43E-14	1.00E-11	22.369313
MIR548D2	1.5523966	16.084854	9.8763003	2.59E-14	1.03E-11	22.306896
MIR548W	1.5554058	11.732138	6.7347089	6.35E-09	1.96E-07	10.191983
NPIPB11	1.5571562	12.159969	9.3315873	2.15E-13	5.39E-11	20.242759
C1QC	1.5662532	7.6615556	3.7178161	0.0004361	0.0020966	-0.545212
COL1A2	1.5684545	5.4819607	5.2147693	2.28E-06	2.58E-05	4.4800349
APOC1	1.5716781	7.6039433	2.8088062	0.0066543	0.0203554	-3.072503
MIR3975	1.5732506	5.2665367	4.9526082	6.01E-06	5.86E-05	3.5426723
IGKV1D-16	1.5758406	6.3505556	3.562846	0.0007151	0.0031666	-1.009944
MIR548A3	1.5836639	10.094731	8.8729887	1.30E-12	2.21E-10	18.485459
MIR103B2	1.5929875	7.15454	7.9080507	5.96E-11	4.39E-09	14.748013
MIR297	1.5937702	5.4977431	9.1059113	5.20E-13	1.05E-10	19.379982
MIR1254-1	1.5957279	9.3501444	10.567633	1.84E-15	1.62E-12	24.88326
EMP1	1.6020243	9.506623	7.428186	4.04E-10	2.05E-08	12.880797
FBLN5	1.6036731	8.4646531	4.3312881	5.56E-05	0.0003765	1.4089021
MIR548H2	1.6075185	13.794603	7.5511838	2.47E-10	1.37E-08	13.359336
SNORA70G	1.6091761	7.6054069	6.4466805	1.98E-08	5.08E-07	9.0843444
CDH6	1.6120388	8.3158515	3.992883	0.0001765	0.000988	0.3095963
RNA5SP20	1.613778	5.493623	7.1616119	1.17E-09	4.87E-08	11.84475
COLEC12	1.6375545	6.6614379	3.4312178	0.0010786	0.0044833	-1.394477
MIR103A2	1.6423253	6.577929	8.5140595	5.37E-12	6.69E-10	17.100169
ADH1B	1.6430805	6.1165436	4.5407219	2.66E-05	0.0002027	2.1127935
RNU7-40P	1.6547488	6.8764592	7.0465925	1.84E-09	7.04E-08	11.398479
SCARNA7	1.658867	10.474747	5.5128324	7.40E-07	1.01E-05	5.5674106
COL8A1	1.6594165	5.6584005	4.8188961	9.80E-06	8.86E-05	3.0723608
RNA5SP320	1.6822207	4.8427854	7.6892646	1.43E-10	8.78E-09	13.896699
FABP5P1	1.6854836	10.747757	4.8022457	1.04E-05	9.30E-05	3.0141903
ECM1	1.7046763	8.7356285	5.9284677	1.50E-07	2.69E-06	7.1153773
VTRNA2-1	1.7129892	6.5705954	3.9976536	0.0001737	0.0009755	0.3247416
ASPN	1.7197864	6.2422	6.3102291	3.38E-08	7.92E-07	8.5625183
FABP5P7	1.7384547	10.916867	4.6683169	1.69E-05	0.0001393	2.5496238
MIR54802	1.7414216	12.218318	9.3541595	1.97E-13	5.06E-11	20.328826
IGKV3D-7	1.7436765	6.9428408	2.7708441	0.0073826	0.0221471	-3.166735
PFN1P2	1.7593908	8.1774984	10.570212	1.82E-15	1.62E-12	24.892773
MIR644A	1.7640407	6.8976377	8.0010193	4.12E-11	3.19E-09	15.10957
OTTHUMG000	1.7822063	7.2261498	7.5792729	2.21E-10	1.25E-08	13.468642
CCL19	1.7901551	6.5255715	3.7251524	0.0004259	0.0020554	-0.5229
FPR3	1.8076302	5.4570666	4.7199382	1.40E-05	0.0001193	2.7279723
MAGI2-AS1	1.8167125	8.446168	6.0223597	1.04E-07	2.00E-06	7.4693242
MIR548X	1.8327225	12.404066	7.6272175	1.83E-10	1.07E-08	13.655226

MIR3911	1.8331282	7.167987	6.7968826	4.97E-09	1.60E-07	10.431992
OTTHUMG00(	1.8361437	5.9546993	7.2005563	9.99E-10	4.28E-08	11.995975
IGKV1D-39	1.8466344	6.8633215	3.1121948	0.002815	0.0099835	-2.284861
IGKV1D-27	1.8615313	6.5861787	3.0079582	0.0038056	0.0127769	-2.562248
MIR5047	1.896104	11.033967	6.8871956	3.47E-09	1.18E-07	10.781109
IGHJ5	1.9490893	4.0243416	3.1573115	0.0024659	0.0089354	-2.162675
C3	1.9642729	6.46047	3.7841803	0.0003517	0.0017528	-0.342385
C7	1.9749617	10.350154	3.9634828	0.0001948	0.0010725	0.2164929
CCL18	1.9775644	4.5903711	3.6255103	0.0005863	0.0026811	-0.823552
MOXD1	2.0920948	5.912727	5.0883198	3.64E-06	3.83E-05	4.0255084
VCAN	2.0956402	7.817848	3.9029058	0.0002382	0.0012612	0.0259411
MIR604	2.1031258	7.0159884	5.8344461	2.16E-07	3.62E-06	6.7623958
MMP7	2.2947783	10.47772	3.6845985	0.0004853	0.0022956	-0.645888
LOC100190(	2.3429597	9.2193797	9.9829109	1.72E-14	7.67E-12	22.707537
RNA5SP310	2.4309625	7.5820321	7.9595051	4.86E-11	3.68E-09	14.948141
RNA5SP316	2.4309625	7.5820321	7.9595051	4.86E-11	3.68E-09	14.948141
MIR4256	2.6723823	6.8521789	12.750569	6.25E-19	3.20E-15	32.634117
VTRNA1-3	2.6785663	6.1756898	11.402088	8.11E-17	1.47E-13	27.918846
CCL21	2.6875278	7.1688911	4.9826696	5.39E-06	5.33E-05	3.6491615
IGHG4	2.8535668	7.9357116	4.6972694	1.52E-05	0.0001277	2.6495409
IGLC7	2.853605	8.3701138	4.0438802	0.0001488	0.0008587	0.4720386
RNA5SP311	2.8731177	7.9474749	8.5836065	4.07E-12	5.41E-10	17.369157
RNA5SP314	2.8731177	7.9474749	8.5836065	4.07E-12	5.41E-10	17.369157
RNA5SP317	2.8731177	7.9474749	8.5836065	4.07E-12	5.41E-10	17.369157
IGHA1	2.9702454	10.138071	3.0528399	0.0033447	0.0114841	-2.443661
LUM	3.0339623	8.1817275	5.7946612	2.51E-07	4.12E-06	6.6134964
RNA5SP312	3.0548325	7.8506875	9.0396826	6.74E-13	1.26E-10	19.126032
RNA5SP315	3.0548325	7.8506875	9.0396826	6.74E-13	1.26E-10	19.126032
RNA5SP313	3.0548325	7.8506875	9.0396826	6.74E-13	1.26E-10	19.126032
RNA5SP195	3.0795511	9.4724482	10.31289	4.85E-15	3.40E-12	23.940016
MIR4521	3.3107969	6.4405777	15.721484	3.12E-23	4.82E-19	42.145499
SFRP2	3.6086786	7.5960143	5.1052011	3.42E-06	3.65E-05	4.0859368
FN1	3.9115869	9.3391849	6.9474619	2.73E-09	9.65E-08	11.014364
