# Supplementary material for: To Develop Biomarkers for Diabetic Nephropathy Based on Genes Related to Fibrosis and Propionate Metabolism and Their Functional Validation
Source: J Diabetes Res. 2024 Oct 16;2024:9066326. doi: 10.1155/2024/9066326 (PMC11498995; doi:10.1155/2024/9066326)
Supplement: Supporting Information 2 — Table S2: 515 DEGs between high- and low-propionate metabolism-related genes (PMRGs) score groups. [file 9066326.f2.pdf]

	logFC	AveExpr	t	P.Value	adj.P.Val	B
MAGI2-AS1	-1.165183	9.0418115	-3.34435	0.0017566	0.0439233	-1.41326
MAGI2-IT1	-1.110721	6.3805149	-3.74078	0.0005556	0.0207128	-0.357005
OTTHUMG00(	-1.086935	6.5567137	-3.886192	0.0003596	0.0149781	0.0447834
MIR3911	-0.991566	7.7690127	-3.409888	0.0014578	0.0387391	-1.242951
OTTHUMG00(	-0.988562	7.6308959	-3.580598	0.0008904	0.0282522	-0.791094
OTTHUMG00(	-0.913449	5.7822612	-4.147538	0.0001621	0.0084922	0.7833876
OTTHUMG00(	-0.883164	4.2890883	-4.404565	7.29E-05	0.0045963	1.5276118
LOC100190(	-0.873153	9.9875632	-3.338409	0.0017864	0.0444169	-1.428607
RNU6-45P	-0.802861	10.628009	-4.523355	5.01E-05	0.0034425	1.8766835
OTTHUMG00(	-0.792567	4.4008737	-3.977028	0.0002732	0.0123255	0.2992201
MIR612	-0.788682	10.779061	-3.636946	0.000755	0.0253347	-0.639461
OTTHUMG00(	-0.780448	5.2140698	-3.504363	0.0011111	0.0329229	-0.994314
LRP2BP	-0.768466	7.2366854	-3.738614	0.0005592	0.0207463	-0.362937
PLCE1-AS1	-0.752552	5.6506988	-3.783258	0.0004896	0.0187763	-0.240365
OTTHUMG00(	-0.711398	4.0647683	-4.825061	1.92E-05	0.001673	2.7753545
RNU7-45P	-0.705617	7.1556024	-3.841514	0.0004113	0.0164445	-0.07941
OTTHUMG00(	-0.705549	6.7263376	-4.667736	3.17E-05	0.0024174	2.3047373
MIR3679	-0.697854	5.9358695	-3.718961	0.0005928	0.0216026	-0.416677
RNY4P26	-0.688354	6.2184395	-4.2166	0.000131	0.0071772	0.9817514
RNU6-3	-0.688114	10.591901	-3.985321	0.0002664	0.0121289	0.3225726
SRGAP2-AS1	-0.681655	4.7639622	-3.508295	0.0010985	0.032676	-0.98389
RNU7-28P	-0.679147	7.0776378	-4.660257	3.25E-05	0.0024529	2.2824712
MIR3682	-0.67123	5.7721722	-3.308123	0.001946	0.0468391	-1.506607
MIR2355	-0.669433	4.34841	-4.765736	2.32E-05	0.0019153	2.597417
MIR2276	-0.665872	4.5449088	-3.347385	0.0017416	0.0437579	-1.405412
RNU7-29P	-0.662202	8.5646941	-3.695934	0.0006346	0.0225811	-0.47947
OTTHUMG00(	-0.658603	9.2357368	-3.38322	0.001573	0.0408526	-1.31247
OTTHUMG00(	-0.65854	6.4969637	-4.119179	0.0001769	0.0091124	0.7022962
LOC1005061	-0.656103	8.0709968	-3.365632	0.0016537	0.0421339	-1.358156
OTTHUMG00(	-0.65422	6.5829941	-4.081857	0.0001984	0.0098485	0.5959137
RNA5SP88	-0.646839	4.4235615	-3.583082	0.000884	0.0281637	-0.784434
KIAA1024L	-0.642914	4.5276893	-3.963287	0.0002848	0.0125933	0.2605674
FTX	-0.637291	8.0905332	-4.531295	4.89E-05	0.0033721	1.9001198
LINC00342	-0.632522	8.3162798	-3.484417	0.001177	0.0341228	-1.04711
RNU6-38	-0.630663	8.7831607	-3.502488	0.0011171	0.033007	-0.999284
OTTHUMG00(	-0.62988	4.7880159	-3.501778	0.0011194	0.0330175	-1.001166
LOC100288(	-0.615799	10.636536	-3.640689	0.0007467	0.0251395	-0.629345
OTTHUMG00(	-0.615426	4.7586085	-3.483061	0.0011816	0.0341284	-1.050693
OTTHUMG00(	-0.610026	5.3154439	-3.376213	0.0016047	0.0412831	-1.330686
CACNA1C-AS	-0.60696	5.711049	-3.427131	0.0013876	0.0376516	-1.197844
RNU7-6P	-0.596126	7.8336466	-3.76444	0.0005179	0.0196856	-0.292113
SMG1P1	-0.595949	11.189278	-3.712365	0.0006044	0.0219254	-0.434684
SCARNA8	-0.591416	3.716181	-4.017446	0.0002416	0.0112946	0.4132342
RNU7-7P	-0.591378	9.9193654	-3.319677	0.0018836	0.0459235	-1.476899
SYNE1-AS1	-0.587142	5.7306651	-3.675822	0.0006734	0.0234884	-0.534162
OTTHUMG00(	-0.583614	5.3927432	-4.049638	0.000219	0.0105567	0.5043864
NPIPB11	-0.582	12.670512	-3.569967	0.0009184	0.0287553	-0.819568
RNU7-25P	-0.577952	7.4318	-4.537358	4.80E-05	0.0033229	1.9180215
RNU7-11P	-0.576806	7.4397278	-4.198081	0.0001387	0.0075068	0.9284396
RNU6-73	-0.575823	7.47036	-3.469215	0.0012297	0.0348747	-1.08724

SLC7A5P2	-0.573374	10.83959	-3.702868	0.0006217	0.0222888	-0.460583
DPYD-IT1	-0.573029	4.519712	-5.396661	3.00E-06	0.000483	4.5128252
GOLGA8B	-0.572111	9.5948105	-3.858398	0.000391	0.0157744	-0.032552
MIR626	-0.567109	4.2388305	-3.321769	0.0018725	0.045783	-1.471511
OTTHUMG00(	-0.565865	6.5145049	-3.99723	0.0002569	0.0118154	0.3561475
RNU6-64P	-0.563073	4.3082602	-3.382474	0.0015764	0.0408708	-1.314411
MIR2116	-0.562138	3.2123944	-3.632021	0.000766	0.0254822	-0.65276
OTTHUMG00(	-0.55638	5.0690041	-3.539911	0.0010023	0.0306746	-0.899836
OTTHUMG00(	-0.55537	6.3431873	-3.575632	0.0009034	0.0284889	-0.804401
LOC595101	-0.550295	10.385345	-3.956931	0.0002904	0.0127292	0.2427075
OTTHUMG00(	-0.547576	5.8481063	-3.281103	0.0020997	0.04931	-1.575859
OTTHUMG00(	-0.545209	4.0435976	-3.969835	0.0002792	0.0124165	0.2789797
RNU6-44	-0.533662	6.0605107	-3.722142	0.0005872	0.0215013	-0.407988
LINC00260	-0.531238	4.3635229	-3.707566	0.0006131	0.0220862	-0.447773
GOLGA8A	-0.52643	9.554839	-3.620235	0.0007929	0.0261813	-0.684554
LOC440354	-0.519563	10.448884	-3.81184	0.0004495	0.0175421	-0.161536
LOC100216[illegible]	-0.513807	5.123678	-3.363171	0.0016653	0.0423419	-1.364539
OTTHUMG00(	-0.509248	5.1989868	-3.589245	0.0008682	0.0278915	-0.767903
NPIPL3	-0.508595	13.3082	-4.071446	0.0002048	0.0100543	0.566306
RNU7-47P	-0.508423	9.3972359	-3.301851	0.0019807	0.0472395	-1.522711
LOC100132[illegible]	-0.505981	8.746301	-3.580643	0.0008903	0.0282522	-0.790973
OTTHUMG00(	-0.503367	5.2476095	-3.593369	0.0008578	0.0276726	-0.756831
OTTHUMG00(	-0.502703	5.1003015	-3.802543	0.0004622	0.0179234	-0.187207
ANKRD36B	-0.501121	7.9399583	-3.362739	0.0016674	0.0423419	-1.365657
SNORD1C	0.5023868	7.600528	4.0836674	0.0001973	0.0098485	0.6010645
AGA	0.5028816	8.62139	3.4139184	0.0014411	0.0384554	-1.23242
NIPSNAP1	0.5034547	6.5633385	5.67546	1.20E-06	0.0003072	5.3705674
ADI1	0.50375	8.2416273	4.7700975	2.29E-05	0.0019033	2.6104794
GPR89C	0.5047766	7.6906451	3.7543451	0.0005336	0.0200946	-0.319825
MCCC2	0.5049383	6.648232	6.8696024	2.36E-08	4.05E-05	9.0547288
ACOX1	0.5056772	8.9431249	4.723439	2.65E-05	0.0021301	2.4708972
LIN7A	0.5061277	6.0246546	3.6972105	0.0006322	0.0225811	-0.475995
DMRTA1	0.506156	4.9657756	4.0823978	0.0001981	0.0098485	0.5974517
ETFB	0.5082524	9.1563149	3.4199007	0.0014167	0.0380383	-1.216774
CLEC18C	0.512661	5.3523905	5.0634323	8.88E-06	0.0009879	3.4954125
LOC1001301	0.5129368	5.1067302	4.1056518	0.0001844	0.0094207	0.663694
TECRP1	0.5153136	12.752356	4.8919083	1.55E-05	0.0014218	2.9764962
DDAH1	0.5154026	5.6701317	5.7190268	1.04E-06	0.0002746	5.5049552
ASPDH	0.5178885	5.618191	4.6181324	3.71E-05	0.0026792	2.1572344
ERBB3	0.5193087	6.0541049	3.5922892	0.0008605	0.0277311	-0.759731
RAB3IP	0.523194	7.4888034	5.0332058	9.80E-06	0.0010403	3.4036958
PROZ	0.523346	4.5116783	4.5350276	4.83E-05	0.00334	1.9111401
WWC1	0.5245293	5.6289763	4.5612411	4.45E-05	0.0031229	1.9886213
FN3K	0.5251851	5.2236393	4.6664248	3.18E-05	0.0024174	2.3008333
MIPEP	0.5258167	6.7243507	7.4581407	3.46E-09	1.41E-05	10.846757
TNFSF15	0.5259063	4.0905637	3.4868407	0.0011688	0.0339486	-1.040702
PSAT1P4	0.5266531	3.2479037	5.295306	4.18E-06	0.0005897	4.2022832
RDH10	0.5277608	6.8465995	5.5135013	2.05E-06	0.0003798	4.8717421
MICU1	0.527894	8.1351417	6.7660539	3.32E-08	4.46E-05	8.7369019
CES3	0.5278965	4.7334905	4.0758602	0.0002021	0.0099601	0.5788554
SLC25A5	0.5280351	9.629669	4.181857	0.0001458	0.007829	0.8818048

PFKFB2	0.528068	6.4609763	4.9503534	1.28E-05	0.0012443	3.1528784
TOM1L1	0.5295417	10.534073	4.9275488	1.38E-05	0.001312	3.0840001
SH3YL1	0.5298953	5.7912693	5.2257752	5.24E-06	0.000675	3.9897547
ETNK2	0.5305919	5.2632066	5.5947397	1.57E-06	0.0003461	5.1217878
CDHR2	0.531068	4.6929515	5.9706648	4.55E-07	0.0001759	6.2822492
STX3	0.5326862	6.9347783	4.3993431	7.41E-05	0.004662	1.5123362
MT1CP	0.5359258	7.9216876	3.4984421	0.0011303	0.0332048	-1.010003
RNF128	0.5363407	5.2788971	3.7132818	0.0006028	0.0219174	-0.432182
GPR89A	0.5365439	9.0796432	4.2497878	0.0001182	0.0067327	1.077512
TREH	0.5378679	4.6212673	3.6171991	0.0008	0.0263871	-0.692735
ZDHHC9	0.5385507	9.3226076	5.8457695	6.87E-07	0.0002081	5.8962722
ACSM5	0.538623	5.1811471	4.1447737	0.0001635	0.0085503	0.7754726
GPR155	0.5396163	6.9691759	3.9838391	0.0002676	0.0121289	0.3183989
ARG2	0.543045	7.1988995	4.90843	1.47E-05	0.0013726	3.0263092
CCNG1	0.5435063	10.16842	6.912342	2.05E-08	3.97E-05	9.1857244
EBP	0.5440922	8.1857659	4.7407841	2.51E-05	0.0020417	2.5227445
MAOB	0.5456955	7.4030566	4.8302194	1.88E-05	0.0016596	2.7908525
SSTR2	0.5458703	4.7289766	3.9656184	0.0002828	0.0125584	0.2671217
MTCH2	0.5460421	12.010195	5.4063378	2.91E-06	0.0004754	4.542514
SUCLG2	0.5462968	10.747547	5.0644582	8.85E-06	0.0009879	3.4985273
GLB1L2	0.5467569	5.9505917	4.9254813	1.39E-05	0.001312	3.0777589
TACO1	0.5486387	8.3114871	5.111169	7.61E-06	0.000891	3.6404778
MYOM3	0.5488212	4.5660776	6.5491979	6.77E-08	6.34E-05	8.0694957
DLST	0.5510691	9.4994639	5.6649437	1.25E-06	0.0003105	5.3381397
ACOX2	0.5519441	6.3167161	3.745871	0.0005473	0.0205257	-0.34306
SLC9A3R1	0.5522259	7.7212929	4.1617304	0.0001552	0.0082121	0.824049
MTHFD1	0.553631	8.4216466	5.9522246	4.84E-07	0.0001759	6.2252466
TM7SF2	0.5538666	6.3621098	6.9243974	1.97E-08	3.97E-05	9.222653
PHYH	0.5578307	7.6415627	4.5034523	5.34E-05	0.003594	1.817992
TSPAN33	0.5610392	8.1772776	3.3143329	0.0019122	0.0461337	-1.490647
POLDIP2	0.56213	9.2514593	6.6415043	4.99E-08	5.72E-05	8.3538496
NCEH1	0.5631687	6.6341093	3.5696496	0.0009193	0.0287553	-0.820417
CTAGE5	0.5666247	8.1768844	5.5976088	1.55E-06	0.0003454	5.130625
XYLB	0.5678922	4.9339246	4.9407802	1.32E-05	0.0012753	3.1239553
QPRT	0.5688071	7.1377312	4.3147021	9.65E-05	0.0057597	1.2656001
FTCD	0.5707454	5.0128795	4.7152481	2.72E-05	0.0021809	2.4464308
STRIP2	0.5709102	4.2308339	4.9791134	1.17E-05	0.0011862	3.2398433
WLS	0.5716329	8.2435676	3.4423801	0.0013283	0.0363605	-1.157851
NQO2	0.5735208	5.9608571	3.5410625	0.000999	0.0306595	-0.896766
NR1H4	0.5767011	5.875261	4.8308954	1.88E-05	0.0016596	2.7928839
PRDX3	0.5777014	12.148466	5.561663	1.75E-06	0.000362	5.0199367
ECI2	0.5794869	7.3165749	5.2177687	5.38E-06	0.0006899	3.9653105
HAVCR2	0.5835897	4.9257678	4.4453819	6.41E-05	0.0041706	1.6472174
C1orf210	0.5861759	5.709732	4.6265924	3.61E-05	0.0026453	2.1823598
KIAA1191	0.5867519	11.344541	5.0453259	9.42E-06	0.0010188	3.4404588
PPARGC1A	0.5894413	5.316921	3.9571128	0.0002902	0.0127292	0.2432193
IDH2	0.5898096	8.0695051	5.0734646	8.60E-06	0.0009733	3.5258773
PNPO	0.5898435	7.2214415	5.6680919	1.23E-06	0.0003105	5.3478469
ACAD11	0.5898817	6.8846985	3.7963466	0.0004709	0.0182355	-0.204301
AHCY	0.5899055	5.5978917	5.8528963	6.71E-07	0.0002079	5.918289
AKR7A2	0.5900606	6.9788027	5.1067007	7.72E-06	0.0008935	3.6268882

UPB1	0.59023	4.6973834	4.5978407	3.96E-05	0.0028321	2.0970241
LOC1004227	0.5940152	5.1579322	3.5813837	0.0008884	0.0282454	-0.788988
GGT6	0.5956229	5.7295841	4.335597	9.04E-05	0.0054806	1.3263571
FAH	0.5969461	6.6572349	4.7463012	2.47E-05	0.0020273	2.5392463
LOC401164	0.5973648	4.1205473	5.0900759	8.15E-06	0.0009291	3.5763466
HSPA4L	0.5986904	6.2375783	4.4217907	6.90E-05	0.0043901	1.5780441
OTTHUMG000	0.5991589	8.3809588	3.4429209	0.0013262	0.0363363	-1.15643
VIL1	0.59993	4.0398702	5.54691	1.83E-06	0.0003666	4.9745275
BNIP3	0.6018727	9.8872302	5.2990467	4.13E-06	0.0005897	4.2137294
AKR1C3	0.6033132	5.9613371	4.2968209	0.0001021	0.0060202	1.2136879
PLS1	0.6036785	5.6382937	4.78099	2.21E-05	0.0018537	2.6431162
SERINC2	0.610616	6.884749	3.9728737	0.0002767	0.0123402	0.2875278
SLC31A1	0.6106838	9.6068693	5.3828682	3.14E-06	0.0004907	4.4705167
UPK1B	0.6129089	5.6087215	3.5778195	0.0008976	0.0283366	-0.79854
NIT2	0.6164925	6.276551	5.2449391	4.92E-06	0.0006449	4.048287
PP7080	0.620571	5.904852	5.580729	1.64E-06	0.0003552	5.0786385
UGT2B28	0.6222996	3.8414673	5.3116226	3.96E-06	0.0005803	4.2522188
PHYHIPL	0.6224436	6.0975261	4.3744976	8.01E-05	0.0049695	1.43974
MAOA	0.6228721	7.4038476	4.2240374	0.000128	0.0070757	1.0031878
RGN	0.6238496	5.4629612	3.6168029	0.000801	0.0263896	-0.693802
HNF4A	0.6248384	5.9056632	5.5388668	1.88E-06	0.0003684	4.9497759
TTC38	0.6283554	6.4159976	5.4522862	2.50E-06	0.0004295	4.6835836
PGPEP1	0.6295375	8.333709	5.4033377	2.94E-06	0.0004776	4.5333082
AKR1A1	0.6324752	7.8203212	5.9089871	5.58E-07	0.0001894	6.0916074
AMACR	0.634649	6.5563746	5.1110398	7.61E-06	0.000891	3.6400846
SERPINE2	0.6352638	5.64778	3.6465823	0.0007339	0.0249525	-0.613411
MAP7	0.6353869	6.7178676	6.120638	2.78E-07	0.0001247	6.745929
SLC37A4	0.6377974	6.3689522	5.2973561	4.15E-06	0.0005897	4.2085561
ISOC2	0.6381101	6.9690754	5.9629064	4.67E-07	0.0001759	6.2582659
ALDH8A1	0.6387506	5.472572	4.2523322	0.0001172	0.006709	1.0848649
COTL1	0.640284	9.5057354	4.8231496	1.93E-05	0.0016786	2.7696133
HNF4G	0.6406129	5.4752173	3.6735906	0.0006778	0.0235933	-0.54022
HSD17B14	0.6411617	5.6523239	4.0103438	0.0002469	0.0114551	0.3931655
ALDH3A2	0.64248	8.2162156	4.9608145	1.24E-05	0.0012222	3.184498
GJB1	0.6445158	6.5370754	5.7873745	8.32E-07	0.000236	5.7159194
CYFIP2	0.6445971	7.548651	4.3687872	8.15E-05	0.0050455	1.4230743
GOT1	0.6446624	7.8557824	4.8486585	1.78E-05	0.0015943	2.8462834
SMIM2-AS1	0.6465517	6.6067051	3.362795	0.0016671	0.0423419	-1.365513
CNDP2	0.649629	9.6157788	5.7500435	9.41E-07	0.000256	5.6006733
BDH2	0.6519341	8.4868205	5.0144453	1.04E-05	0.0010797	3.3468265
GSTA5	0.6521046	4.6613078	3.6093385	0.0008187	0.0268014	-0.713901
HNF1B	0.6531302	8.0612956	3.9847266	0.0002669	0.0121289	0.320899
GGTLC1	0.6554771	5.2028532	6.210859	2.06E-07	0.0001087	7.0248518
PXMP2	0.6557593	7.7635907	3.5720266	0.0009129	0.0287304	-0.814055
SUSD2	0.6576543	5.6469368	5.2636559	4.63E-06	0.0006172	4.1054866
C9orf66	0.6611991	4.1833115	4.5649071	4.39E-05	0.0031009	1.9994678
EPB41L3	0.66199	6.232598	6.1915775	2.20E-07	0.0001114	6.9652477
OTTHUMG000	0.662259	13.676366	5.063275	8.89E-06	0.0009879	3.4949347
ACSL1	0.6635968	8.4385176	3.8923415	0.000353	0.0147716	0.0619276
MSRA	0.6640563	5.0259534	5.5459302	1.84E-06	0.0003666	4.9715121
METTL7A	0.6672337	8.3824254	5.335649	3.66E-06	0.0005549	4.3257905

SNORA59B	0.6734133	5.5932295	4.3987406	7.42E-05	0.004662	1.5105742
OTTHUMG00(	0.6742882	6.5936556	3.8281656	0.0004281	0.0168758	-0.116389
AGMO	0.6744831	4.516352	5.4855731	2.24E-06	0.0003983	4.7858689
SLC51B	0.6746358	5.5215441	6.2090919	2.07E-07	0.0001087	7.0193891
LYG1	0.676474	5.1198017	3.6552624	0.0007154	0.0245829	-0.589917
GALNT11	0.6773485	8.2642356	5.0806935	8.40E-06	0.0009543	3.5478365
SLC25A10	0.6789094	4.7476571	5.9540819	4.81E-07	0.0001759	6.2309877
MYO5B	0.6793286	5.9705278	5.0439389	9.46E-06	0.0010188	3.4362509
SMPDL3A	0.6796315	6.9505729	4.7937358	2.12E-05	0.0017991	2.6813297
EMX2	0.6808187	5.915359	3.8055431	0.0004581	0.0178305	-0.178927
LINC00671	0.6814631	6.0048261	3.3465386	0.0017458	0.0437579	-1.407602
C12orf39	0.6862405	5.3494285	3.2961329	0.0020129	0.0478885	-1.537377
PCCA	0.6901571	8.7606151	5.1689233	6.31E-06	0.0007765	3.8163217
C19orf69	0.6904246	5.4428861	4.3582098	8.43E-05	0.0051824	1.3922241
BPHL	0.6916837	7.60036	4.5142954	5.16E-05	0.0035202	1.849957
SLIT2	0.69376	6.28971	4.0161927	0.0002425	0.0113036	0.4096923
ACADL	0.6937867	4.7138027	6.1470485	2.55E-07	0.0001192	6.8275844
LGALS2	0.694629	8.1682661	3.7859649	0.0004857	0.0186691	-0.232911
GGT3P	0.6953732	5.6435002	4.933344	1.35E-05	0.001302	3.1014972
IDH1	0.6957866	7.5432805	4.8805501	1.60E-05	0.0014574	2.9422736
ANKS4B	0.7000884	3.7961359	4.4763365	5.81E-05	0.0038645	1.7381618
LOC100506(	0.7016613	5.145511	3.6781321	0.0006688	0.0233552	-0.527886
MGST1	0.7020117	4.720852	5.0986381	7.92E-06	0.0009138	3.602373
ABCC6P1	0.7026921	5.376452	4.065871	0.0002083	0.0101561	0.5504645
SLC9A3	0.7031102	5.2509161	5.830228	7.23E-07	0.0002148	5.8482636
TOX3	0.7032447	5.722559	4.3683712	8.16E-05	0.0050455	1.4218604
SLC28A1	0.7043075	5.4655463	5.0568693	9.07E-06	0.001005	3.4754889
CIRH1A	0.7067337	8.463818	7.6424852	1.91E-09	1.21E-05	11.401896
HADH	0.7068223	6.7096929	5.9594188	4.72E-07	0.0001759	6.2474848
TMEM176B	0.7084007	7.5797554	6.0823067	3.15E-07	0.0001352	6.6274135
DHTKD1	0.7094054	7.1648015	5.5804006	1.64E-06	0.0003552	5.077627
LDHB	0.7097871	12.25792	5.9164835	5.44E-07	0.0001879	6.1147754
ANK2	0.7132167	6.8799456	3.7094595	0.0006097	0.0220372	-0.44261
CYB5A	0.718041	13.474617	3.7105678	0.0006077	0.0219907	-0.439587
STRADB	0.718468	8.1240173	5.0536578	9.17E-06	0.0010083	3.4657415
LPPR1	0.7292384	6.2136954	3.5566691	0.0009547	0.0296828	-0.855124
ALPL	0.7306567	5.4889983	4.4346517	6.63E-05	0.0042778	1.6157398
SAT2	0.7316094	9.5207093	4.0110089	0.0002464	0.0114491	0.3950443
MIA2	0.733808	5.7325854	3.9754297	0.0002745	0.012338	0.2947205
NPY6R	0.7343812	6.5659405	3.480325	0.001191	0.0343345	-1.05792
ABP1	0.7416558	6.120971	4.2918033	0.0001037	0.0060918	1.1991348
HOGA1	0.7440902	5.4543649	5.1413759	6.90E-06	0.0008294	3.7324038
CAPN3	0.7457286	6.1335876	5.3814296	3.15E-06	0.0004907	4.4661049
KMO	0.7478641	5.5875963	4.7599164	2.36E-05	0.0019461	2.5799914
UGT2B11	0.7480493	4.0493412	5.4074124	2.90E-06	0.0004754	4.5458116
SLC15A1	0.7492009	5.3389885	4.1374161	0.0001673	0.0087168	0.754419
DNAJC12	0.7518665	5.6558663	4.9764529	1.18E-05	0.0011914	3.2317939
EPHX2	0.7527784	7.1241724	3.6927532	0.0006405	0.0226759	-0.48813
APOE	0.7531106	5.5686766	6.8126442	2.85E-08	4.46E-05	8.8799803
ENPP3	0.7543638	4.840292	4.9720997	1.19E-05	0.001196	3.218625
TMEM106A	0.7558459	8.4858776	4.6905751	2.95E-05	0.0022942	2.372801

AKR7A3	0.7561609	5.3363439	4.7381473	2.53E-05	0.0020482	2.5148594
OTTHUMG000	0.7565957	4.1052051	4.0227105	0.0002377	0.0112165	0.428121
CPN2	0.7600936	5.5006702	8.6730753	7.22E-11	2.23E-06	14.43543
RAB7L1	0.7608703	7.0718671	7.0320615	1.39E-08	3.57E-05	9.5520251
SLC22A7	0.7637036	5.5330193	3.6788958	0.0006673	0.0233552	-0.525812
MRO	0.7649173	4.9118344	3.3969183	0.0015128	0.0397224	-1.276799
CTSB	0.7663248	10.229967	6.0621809	3.37E-07	0.0001402	6.5651866
EPS8	0.7760811	8.794881	3.7081914	0.000612	0.0220862	-0.446069
TRHDE	0.7787596	5.2863754	6.0548871	3.45E-07	0.0001402	6.5426352
SLC39A5	0.7791247	5.3325449	6.0037424	4.08E-07	0.0001624	6.3845079
GPR56	0.7793457	9.9389466	3.4521716	0.0012915	0.0358229	-1.13212
SLC22A12	0.780058	5.6259929	5.1243527	7.29E-06	0.0008632	3.6805864
ACADM	0.7820041	9.7467817	4.8334404	1.87E-05	0.0016519	2.8005317
CAMK2N1	0.7857035	10.48229	4.8648719	1.69E-05	0.0015283	2.8950654
CLYBL	0.7896551	6.819381	5.2077896	5.56E-06	0.0007011	3.9348532
HABP2	0.7899081	5.0013161	3.4883149	0.0011638	0.0339001	-1.036805
TMEM176A	0.7910813	5.5572	7.8878113	8.67E-10	8.93E-06	12.135279
FTLP3	0.7949955	16.793988	5.9148452	5.47E-07	0.0001879	6.1097121
ACSF2	0.7965817	6.1201788	5.7134178	1.06E-06	0.0002757	5.4876494
MTTP	0.8003963	4.4845871	6.0752349	3.23E-07	0.0001365	6.6055483
OTTHUMG000	0.8058385	7.3932576	4.6668295	3.18E-05	0.0024174	2.3020386
ALDH2	0.8082324	8.9427532	6.1019262	2.95E-07	0.0001304	6.6880748
PRLR	0.8113182	5.6921612	5.6220548	1.43E-06	0.0003321	5.2059388
ENPP6	0.81292	4.4170827	5.663808	1.25E-06	0.0003105	5.3346378
OTTHUMG000	0.8131858	6.0785088	3.8186232	0.0004405	0.0172769	-0.142789
USH1C	0.8146553	5.546272	5.2762094	4.45E-06	0.0006108	4.1438689
PCK2	0.8212069	6.5087532	5.5072653	2.09E-06	0.0003798	4.8525635
MCCD1	0.8246229	5.3950273	5.3809059	3.16E-06	0.0004907	4.4644988
AGPAT9	0.8268531	6.0344741	5.0248829	1.01E-05	0.0010507	3.3784611
ACO2	0.8279104	8.2139027	6.0962197	3.01E-07	0.000131	6.6704311
ASPA	0.8290979	7.6178449	3.6354165	0.0007584	0.0253457	-0.643592
PTER	0.8324601	7.0141495	4.3878112	7.68E-05	0.0047953	1.478624
SLC5A10	0.8349213	6.6795761	4.7087078	2.78E-05	0.0022053	2.4269028
CRYZ	0.8353337	9.6887459	6.2909212	1.58E-07	9.24E-05	7.2722906
SLC10A2	0.8367882	4.5749073	3.5659122	0.0009293	0.0289822	-0.830417
GPR137B	0.8371263	9.0974305	6.7368548	3.65E-08	4.70E-05	8.6471707
PARM1	0.837173	9.9774934	4.8033651	2.05E-05	0.0017589	2.7102165
IYD	0.8406015	5.2581615	5.791909	8.20E-07	0.0002347	5.7299209
CMBL	0.8433445	5.3021534	5.131313	7.13E-06	0.0008471	3.7017693
PNP	0.8435791	7.2525656	6.1637428	2.41E-07	0.0001145	6.8791975
TCN2	0.8477829	6.9577393	7.4059137	4.10E-09	1.41E-05	10.68889
C7orf10	0.8483799	5.615089	5.3544396	3.44E-06	0.0005263	4.3833623
UGT2B10	0.8498246	4.5020285	6.1638912	2.41E-07	0.0001145	6.8796561
KCNK5	0.8506476	5.050458	5.4987935	2.15E-06	0.0003837	4.8265127
TM7SF3	0.8533423	10.077859	5.818514	7.51E-07	0.0002191	5.8120825
OCIAD2	0.8695695	10.24682	5.1416542	6.89E-06	0.0008294	3.7332513
GPD1	0.8737029	5.8310663	4.9587174	1.25E-05	0.0012266	3.1781584
OGDHL	0.8746112	5.9549966	6.1707522	2.35E-07	0.0001145	6.9008675
DHRS4-AS1	0.874652	7.32047	6.3209714	1.43E-07	8.90E-05	7.3651351
OTTHUMG000	0.8747574	5.037102	4.6663716	3.18E-05	0.0024174	2.300675
UGT3A1	0.875336	4.8968173	6.3015098	1.53E-07	9.09E-05	7.3050077

CLDN10	0.8772386	7.9395559	3.9855494	0.0002662	0.0121289	0.323217
TPMT	0.8802349	9.0050685	6.3267736	1.41E-07	8.90E-05	7.3830597
LACTB2	0.8807576	8.1567907	5.5747519	1.67E-06	0.0003593	5.0602335
SUCLG1	0.8828023	9.1542795	5.4173309	2.80E-06	0.000466	4.5762512
FOLR1	0.8838484	6.8761359	4.7013205	2.85E-05	0.0022452	2.4048548
HRASLS2	0.8875393	4.0023288	3.8746891	0.0003723	0.0153226	0.0127472
SLC22A11	0.8908295	5.4698095	6.5491671	6.77E-08	6.34E-05	8.0694007
PDZK1IP1	0.8909238	10.976408	4.5141849	5.16E-05	0.0035202	1.8496309
PAIP2B	0.8931225	6.6058205	5.3786448	3.18E-06	0.0004918	4.4575648
FRK	0.8932564	7.484238	5.5520056	1.80E-06	0.0003661	4.99021
OTTHUMG000	0.8956066	5.4234268	4.2714581	0.0001105	0.0064169	1.1401874
SLC1A1	0.8963525	6.3379039	5.5112524	2.06E-06	0.0003798	4.8648255
AGT	0.9016615	5.6014946	5.1085536	7.67E-06	0.0008935	3.6325233
GALM	0.9048262	7.1058366	5.0260973	1.00E-05	0.0010507	3.3821425
TFCP2L1	0.9078427	6.4143261	3.644336	0.0007388	0.0249994	-0.619486
CLCN5	0.9091384	6.4195102	5.5134128	2.05E-06	0.0003798	4.8714697
ABCC6P2	0.9152246	5.4796671	5.1074684	7.70E-06	0.0008935	3.6292227
SERPINF2	0.9166406	6.0677554	5.6851397	1.17E-06	0.0003001	5.4004193
SULT1C2	0.9168015	7.2987798	4.8204063	1.95E-05	0.0016887	2.7613737
NAPSA	0.9221907	5.4690476	5.0449447	9.43E-06	0.0010188	3.4393021
C11orf54	0.9239964	11.724461	3.9001701	0.0003448	0.0145238	0.0837701
SLC5A2	0.9271886	5.7163415	6.7860266	3.11E-08	4.46E-05	8.7982527
LOC727944	0.9301013	5.5716554	3.7116327	0.0006058	0.0219472	-0.436682
SLC47A2	0.9326805	5.7912649	5.6326601	1.38E-06	0.0003321	5.2386209
ARSE	0.9426342	6.7213851	4.8136619	1.99E-05	0.001716	2.7411218
ALDH1A1	0.9483521	10.099288	4.494588	5.49E-05	0.003672	1.7918785
CCL14	0.9523768	5.8923593	5.6251741	1.42E-06	0.0003321	5.2155511
CYP24A1	0.9634042	4.871562	3.9222568	0.0003225	0.0138055	0.1454974
SERPINA6	0.9680382	5.9209824	5.6344285	1.38E-06	0.0003321	5.2440711
ACSS3	0.9685856	7.0397263	6.057853	3.42E-07	0.0001402	6.5518051
FUT6	0.968737	6.0716644	5.5580076	1.77E-06	0.000362	5.0086841
GGH	0.9696832	6.42092	6.4075329	1.08E-07	8.33E-05	7.6324651
CDHR5	0.9728508	6.0178329	5.8597943	6.56E-07	0.0002079	5.9396
OTTHUMG000	0.9761537	6.1226327	3.9510785	0.0002956	0.0129022	0.2262749
SLC23A1	0.9764286	6.564082	4.0626776	0.0002104	0.0102172	0.5413941
NPL	0.9768061	6.7787468	6.2322078	1.92E-07	0.0001059	7.090841
AIFM1	0.9824338	9.2550473	6.6603227	4.69E-08	5.58E-05	8.4117745
GGTLC2	0.9831615	7.0903195	5.7614796	9.06E-07	0.0002524	5.6359734
CRYL1	0.9839443	7.2649605	5.8216157	7.44E-07	0.0002189	5.8216625
FOLH1	0.9847099	6.0192361	5.2777361	4.43E-06	0.0006108	4.1485375
IMPA2	0.9874936	7.6195634	6.2274945	1.95E-07	0.0001059	7.0762728
ALDH1B1	0.9890369	6.7821059	6.3613088	1.26E-07	8.90E-05	7.489733
PLA2G12B	0.9915766	4.8372422	6.5986374	5.75E-08	5.92E-05	8.2218414
CYP2B6	0.9919729	5.4047232	4.357908	8.43E-05	0.0051824	1.3913441
HHLA2	0.9924258	5.0003737	4.6928532	2.93E-05	0.0022891	2.3795951
SHMT1	0.9942125	7.5403893	5.8527788	6.71E-07	0.0002079	5.9179259
FLJ22763	0.9983581	4.6721332	5.2979524	4.14E-06	0.0005897	4.2103807
LGMN	0.9999214	12.191405	6.1198017	2.78E-07	0.0001247	6.7433435
ACAT1	1.0129191	8.6966317	5.1845326	5.99E-06	0.000744	3.8639074
A1CF	1.0145181	4.9330017	5.5830794	1.63E-06	0.0003552	5.0858762
CRYAA	1.0185791	7.0529693	5.2658078	4.60E-06	0.0006155	4.112065

AZGP1	1.0209536	6.0533424	4.769369	2.29E-05	0.0019033	2.6082972
MIR509-2	1.0211003	5.1618856	3.5121534	0.0010863	0.0324111	-0.973652
FREM2	1.0218964	5.8585463	5.6623223	1.26E-06	0.0003105	5.3300572
SLC16A4	1.0226972	11.480119	6.8071844	2.90E-08	4.46E-05	8.8632197
ACY3	1.0260963	6.4595195	4.655243	3.30E-05	0.0024739	2.2675466
OTTHUMG00(	1.031915	5.7536459	4.7096185	2.77E-05	0.0022053	2.4296216
OTTHUMG00(	1.0340362	6.6584683	3.9619046	0.000286	0.0126101	0.2566825
DPEP1	1.0357952	6.1570807	5.5652179	1.73E-06	0.000362	5.0308802
SLC16A12	1.0392922	6.2592344	4.1120626	0.0001808	0.009283	0.6819825
KHK	1.0461201	5.4512451	5.6282055	1.40E-06	0.0003321	5.2248929
CES2	1.0496286	7.3547846	6.3137553	1.47E-07	8.90E-05	7.3428413
C4B	1.0521848	7.8829429	3.5488844	0.0009766	0.0301711	-0.875907
GPX3	1.0615638	15.106788	5.3278334	3.76E-06	0.0005638	4.301853
PEPD	1.0663609	10.330877	5.0635214	8.88E-06	0.0009879	3.4956829
SLC44A4	1.0698426	6.6042232	5.542274	1.86E-06	0.0003666	4.9602604
GGTLC3	1.0774103	5.9627473	5.5140885	2.04E-06	0.0003798	4.873548
CTSH	1.0789236	7.8718834	6.272986	1.68E-07	9.44E-05	7.2168692
EHHADH	1.0853458	6.8113378	4.7432661	2.49E-05	0.0020363	2.5301675
CYP4F3	1.0859854	5.8840439	5.6131243	1.48E-06	0.0003354	5.1784222
FCAMR	1.0899322	5.5179824	7.5606523	2.49E-09	1.28E-05	11.155873
ECHS1	1.090323	9.9553041	5.1805663	6.07E-06	0.0007507	3.8518136
AOX1	1.0909225	7.8332017	3.3957811	0.0015177	0.0397377	-1.279764
DPP4	1.0922054	8.9994693	4.9072311	1.47E-05	0.0013738	3.0226932
CA12	1.0929516	9.6445705	4.0863536	0.0001957	0.0097852	0.6087099
KL	1.0933293	10.422403	4.9575498	1.25E-05	0.0012273	3.1746286
DSP	1.0997405	7.7440612	3.3311169	0.0018237	0.0450448	-1.447424
ABCB1	1.1023659	6.9299834	4.7137102	2.74E-05	0.002186	2.4418383
SLC7A8	1.110127	12.090875	4.0064887	0.0002498	0.011553	0.3822777
UGT1A5	1.1156401	5.9523239	6.4968334	8.04E-08	7.31E-05	7.9080333
ENTPD5	1.1160629	8.177331	5.3987811	2.98E-06	0.0004822	4.519328
GK	1.119719	7.9649556	4.1108979	0.0001815	0.0093008	0.6786589
ACO1	1.1202024	10.136832	6.8898645	2.21E-08	4.02E-05	9.1168456
SLC6A13	1.1203472	6.4774749	4.9715097	1.20E-05	0.001196	3.2168405
EPCAM	1.1295614	8.9690459	5.050788	9.25E-06	0.0010141	3.4570324
ADH6	1.1331142	5.5650176	5.1536215	6.63E-06	0.0008065	3.7696979
USP2	1.1351679	6.3755424	4.7981427	2.09E-05	0.0017787	2.6945481
SLC13A2	1.1464813	5.8415541	5.9183665	5.41E-07	0.0001879	6.1205951
SLC22A2	1.1489954	8.5835659	4.7675015	2.30E-05	0.0019096	2.6027039
IL17RB	1.1583676	6.1576807	5.3819255	3.15E-06	0.0004907	4.4676255
SORD	1.159104	6.4756944	5.4601407	2.44E-06	0.0004233	4.7077127
SLC7A7	1.1619643	7.9529515	5.0250462	1.01E-05	0.0010507	3.378956
CFI	1.1636124	10.416226	4.210209	0.0001336	0.0072813	0.9633432
ASS1	1.176295	12.359098	5.6248342	1.42E-06	0.0003321	5.2145037
CDH16	1.1767959	6.8625461	5.0900648	8.15E-06	0.0009291	3.5763129
CTXN3	1.1808712	6.4683215	3.2934525	0.0020281	0.0481398	-1.544247
GLDC	1.1810581	5.6795827	6.168261	2.37E-07	0.0001145	6.8931657
CLRN3	1.1841941	5.4763315	5.3133398	3.94E-06	0.0005798	4.2574756
PRODH2	1.190039	7.2248427	3.7358455	0.0005638	0.0208674	-0.370516
PRAP1	1.1920455	7.5982434	6.3331278	1.38E-07	8.90E-05	7.4026885
DCXR	1.1992296	11.253039	4.7085347	2.78E-05	0.0022053	2.426386
ACAA2	1.2000418	11.070728	4.9552281	1.26E-05	0.0012326	3.167611

ABCC2	1.2222105	5.3281698	5.5506145	1.81E-06	0.0003661	4.9859285
DPYS	1.2332332	5.9908485	5.3913104	3.05E-06	0.0004865	4.49641
NOX4	1.2338476	8.2935017	7.4380409	3.70E-09	1.41E-05	10.786031
FABP3	1.2469079	8.7141437	4.8942078	1.53E-05	0.0014163	2.983427
ALDH4A1	1.2586772	6.9217078	6.7243939	3.80E-08	4.70E-05	8.6088637
PBLD	1.2611644	7.9308198	5.4529322	2.50E-06	0.0004295	4.6855678
ABAT	1.2614794	6.9013171	5.5083679	2.08E-06	0.0003798	4.8559545
AK4	1.266677	8.7303298	5.2884889	4.27E-06	0.0005948	4.1814268
PIPOX	1.2770767	6.837972	5.3054796	4.04E-06	0.0005851	4.2334159
MGAM	1.2807581	5.8480171	4.5282855	4.93E-05	0.0033968	1.8912338
PAQR5	1.2871184	7.5892488	6.9138395	2.04E-08	3.97E-05	9.1903123
LOC728290	1.2902671	4.766659	6.2834642	1.62E-07	9.29E-05	7.2492483
PLGLA	1.2934025	7.132201	4.4566202	6.19E-05	0.0040512	1.680212
ACY1	1.323322	7.3306366	5.2702518	4.53E-06	0.0006146	4.1256517
FABP1	1.325206	8.3162676	4.0996624	0.0001878	0.0095169	0.6466181
CLDN2	1.3269369	8.1477461	5.9635899	4.66E-07	0.0001759	6.2603788
FMO4	1.3303497	7.9026956	4.3969964	7.46E-05	0.0046686	1.5054735
FOLH1B	1.3334245	5.607559	5.8526117	6.72E-07	0.0002079	5.9174098
SPP1	1.3409719	15.490829	3.6215077	0.00079	0.0261397	-0.681123
OTTHUMG00(	1.3493263	8.2721254	5.483921	2.26E-06	0.0003983	4.7807906
ACMSD	1.3571816	6.8178573	4.9655928	1.22E-05	0.0012112	3.1989458
GBA3	1.4023988	6.6059763	4.2273963	0.0001267	0.0070276	1.012873
PDZK1P1	1.4049465	11.22997	4.4955107	5.47E-05	0.0036693	1.794596
ACADSB	1.4062237	9.9252124	5.619166	1.45E-06	0.0003321	5.1970377
DMGDH	1.4076267	7.4119685	5.5155203	2.03E-06	0.0003798	4.8779518
HRSP12	1.4147226	9.8289395	4.9664555	1.22E-05	0.0012112	3.2015547
CYP17A1	1.416194	6.2499837	3.4214063	0.0014106	0.0380383	-1.212834
GDA	1.4401698	7.1763529	6.3204147	1.44E-07	8.90E-05	7.363415
PDZK1	1.445952	12.014758	4.6429193	3.43E-05	0.0025363	2.2308863
TM4SF5	1.4790584	7.7658095	4.6545726	3.31E-05	0.0024739	2.2655516
PCK1	1.4802679	7.1061105	4.0863614	0.0001957	0.0097852	0.608732
DAO	1.4849288	6.6555293	4.5174119	5.11E-05	0.0035	1.8591485
SLC2A2	1.5045444	6.3297178	6.1407668	2.60E-07	0.0001199	6.8081631
VNN1	1.5052288	5.9429359	4.6230781	3.65E-05	0.0026508	2.1719212
LOC1009962	1.5142042	6.3431395	4.9638224	1.23E-05	0.0012143	3.1935925
GIPC2	1.5213864	7.5130322	5.9166035	5.44E-07	0.0001879	6.1151463
FBP1	1.522704	7.2060885	5.2474528	4.88E-06	0.0006423	4.0559671
PLG	1.5271656	6.7416993	5.047255	9.36E-06	0.0010186	3.4463117
DDC	1.5397043	7.446999	4.9252799	1.39E-05	0.001312	3.077151
WDR72	1.5898765	8.2364395	5.8491656	6.79E-07	0.0002079	5.9067636
ALDH6A1	1.5953031	9.8008515	5.2963925	4.16E-06	0.0005897	4.2056077
SLC3A1	1.5982129	9.7781559	5.0925754	8.08E-06	0.0009285	3.5839434
LRP2	1.6035411	11.026623	4.9271408	1.38E-05	0.001312	3.0827684
HGD	1.6084833	7.6932383	5.3533632	3.46E-06	0.0005263	4.3800638
BHMT2	1.6221586	10.906332	4.9978857	1.10E-05	0.0011313	3.296665
SLCO4C1	1.6229479	7.1221405	5.5020319	2.13E-06	0.0003819	4.8364705
RNF152	1.6378726	8.5204654	6.611323	5.52E-08	5.88E-05	8.2609149
CXCL14	1.6457258	11.221021	4.6674466	3.17E-05	0.0024174	2.3038763
TINAG	1.7091644	7.7959332	6.4378489	9.76E-08	7.90E-05	7.7260437
AQP2	1.729896	5.4129593	3.7286278	0.000576	0.0212175	-0.390261
TSPAN1	1.7334781	10.549238	5.3240498	3.80E-06	0.0005653	4.2902665

KCNJ15	1.7429265	8.508731	5.8664822	6.42E-07	0.0002079	5.9602633
BBOX1	1.7486613	10.582039	4.9191576	1.42E-05	0.00133	3.0586736
DIO1	1.7638739	6.5551951	5.8341503	7.14E-07	0.0002141	5.8603792
SLC36A2	1.7693389	7.4566498	4.6290049	3.59E-05	0.0026384	2.1895272
SLC16A9	1.8033801	8.125581	5.3314154	3.71E-06	0.0005599	4.3128232
SLC47A1	1.8226559	7.8592424	6.2006137	2.13E-07	0.0001099	6.9931812
G6PC	1.8239613	7.3775866	4.6246372	3.64E-05	0.0026453	2.176552
LRRC19	1.8602428	7.8593378	6.3145904	1.47E-07	8.90E-05	7.3454213
GLYATL1	1.8606182	7.9634476	4.2165683	0.000131	0.0071772	0.9816606
SLC6A19	1.8815999	7.4174388	5.0316667	9.84E-06	0.0010419	3.3990286
AK4P3	1.890724	8.6452366	5.304787	4.05E-06	0.0005851	4.2312963
RBP5	1.9011306	11.637667	5.4443941	2.57E-06	0.0004365	4.6593429
NAT8B	1.9160234	10.732386	5.1920293	5.85E-06	0.000729	3.8867704
LOC1005059	1.9555058	6.3018185	4.3116435	9.75E-05	0.0057995	1.256715
CYP4F2	1.9745285	7.2465249	4.053568	0.0002163	0.0104632	0.5155358
SLC27A2	1.9828697	7.8292017	5.0546755	9.14E-06	0.0010083	3.4688303
FAM151A	1.9941285	7.9232649	4.8363963	1.85E-05	0.001641	2.8094157
METTL7B	2.009705	7.0992056	7.1259362	1.02E-08	3.16E-05	9.838542
PSAT1	2.0328154	8.3205746	5.3244625	3.80E-06	0.0005653	4.2915303
GATM	2.0640581	12.764471	4.5094375	5.24E-05	0.0035542	1.835633
GSTA2	2.0665119	10.770648	3.3398775	0.001779	0.0443396	-1.424815
ANGPTL3	2.0780636	6.6475576	6.4466135	9.48E-08	7.90E-05	7.7530929
SLC7A9	2.0796803	7.2146656	6.6178481	5.40E-08	5.88E-05	8.2810106
XPNPEP2	2.1157442	8.2456749	5.4326602	2.67E-06	0.0004456	4.6233102
SLC22A6	2.1302725	9.0418527	4.9898687	1.13E-05	0.0011533	3.2723929
UGT2A3	2.1612929	8.7146532	5.5038881	2.11E-06	0.0003818	4.8421781
ACE2	2.1613278	7.4386485	5.9895537	4.28E-07	0.0001673	6.3406425
BHMT	2.1731359	11.053596	4.648417	3.37E-05	0.0025103	2.2472373
ACSM2A	2.1773645	10.10532	5.4439399	2.57E-06	0.0004365	4.6579482
SLC17A3	2.1853736	8.1130527	6.483923	8.39E-08	7.41E-05	7.8682096
NAT8	2.1859866	9.6446032	5.0702309	8.69E-06	0.00098	3.5160563
FMO1	2.1890233	8.8131576	5.5236052	1.98E-06	0.0003798	4.902821
APOM	2.1925019	9.1370393	5.6237601	1.43E-06	0.0003321	5.2111937
SLC22A8	2.1999405	9.1186078	5.2662209	4.59E-06	0.0006155	4.113328
CYP4A11	2.206774	8.4136739	3.7424306	0.0005529	0.0206364	-0.352486
SLC17A1	2.2273108	8.7499802	6.4314045	9.97E-08	7.90E-05	7.7061536
ALDOB	2.2499526	15.85531	4.6277775	3.60E-05	0.0026425	2.1858807
ANPEP	2.311264	12.330554	6.5583345	6.57E-08	6.34E-05	8.0976574
ACSM2B	2.3284284	11.449194	5.2885288	4.27E-06	0.0005948	4.1815488
TMEM27	2.3338073	9.8290722	5.7265764	1.02E-06	0.0002709	5.5282504
PAH	2.3544109	10.475608	4.8427799	1.81E-05	0.0016149	2.8286061
SLC13A3	2.3686135	9.3496688	5.8728783	6.28E-07	0.0002076	5.9800255
HPD	2.3925722	8.4115468	4.9435513	1.31E-05	0.0012679	3.1323261
UGT2B7	2.4046997	12.179871	5.8490752	6.79E-07	0.0002079	5.9064843
AGXT2	2.4092968	8.7227066	5.9478841	4.91E-07	0.0001763	6.2118297
HAO2	2.4142943	7.8216498	4.6094102	3.82E-05	0.0027363	2.1313441
FXYD4	2.4170546	6.9156271	3.4768773	0.0012029	0.0345485	-1.067024
SLC4A4	2.4616226	9.2213271	6.3924331	1.13E-07	8.46E-05	7.5858456
SLC34A1	2.4968848	8.8035007	6.4391294	9.72E-08	7.90E-05	7.7299958
C19orf77	2.5090485	12.264155	5.1625644	6.44E-06	0.0007896	3.7969436
MIOX	2.5180978	9.0070134	4.8002273	2.08E-05	0.0017717	2.700802

CUBN	2.6034539	11.137305	4.9757335	1.18E-05	0.0011914	3.2296173
SLC13A1	2.6096951	8.7432256	6.9384833	1.88E-08	3.97E-05	9.2657897
GLYAT	2.6189255	10.301585	5.3214097	3.84E-06	0.0005674	4.2821824
TMEM174	2.6469771	8.7218593	5.2082635	5.55E-06	0.0007011	3.9362992
AGMAT	2.6553113	8.8536295	5.226242	5.23E-06	0.000675	3.99118
SLC5A12	2.6554098	10.692532	5.2094164	5.53E-06	0.0007011	3.9398175
