# Supplementary figures and images for: To Develop Biomarkers for Diabetic Nephropathy Based on Genes Related to Fibrosis and Propionate Metabolism and Their Functional Validation
Source: J Diabetes Res. 2024 Oct 16;2024:9066326. doi: 10.1155/2024/9066326 (PMC11498995; doi:10.1155/2024/9066326)

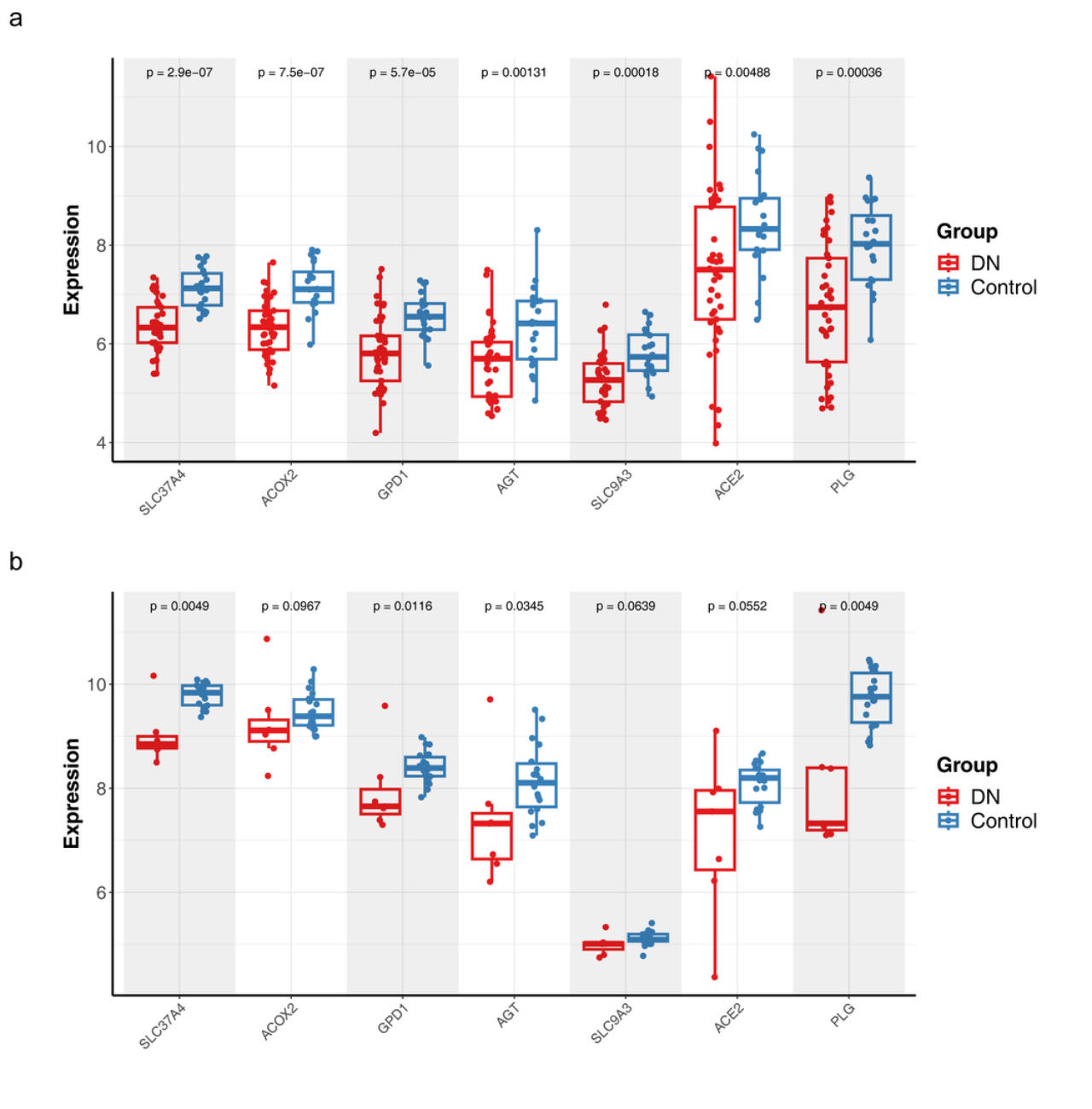

Supplement: Supporting Information 3 — Figure S1: the expression of biomarkers in DN and control samples. a: GSE96804; b: GSE104948. [file 9066326.f3.jpg]

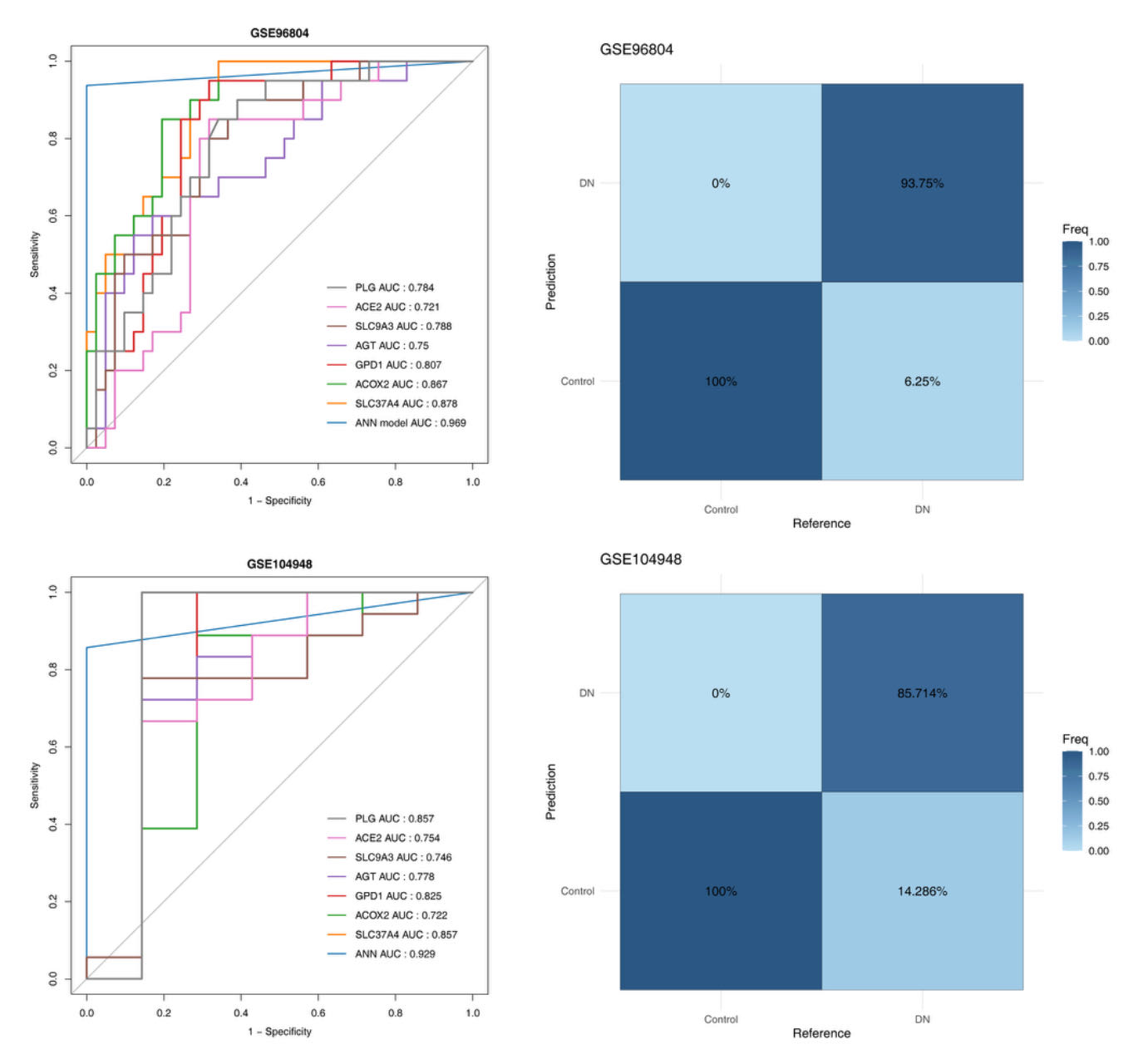

Supplement: Supporting Information 4 — Figure S2: the ROC curves and confusion matrix of biomarkers in GSE96804 and GSE104948 datasets. [file 9066326.f4.jpg]

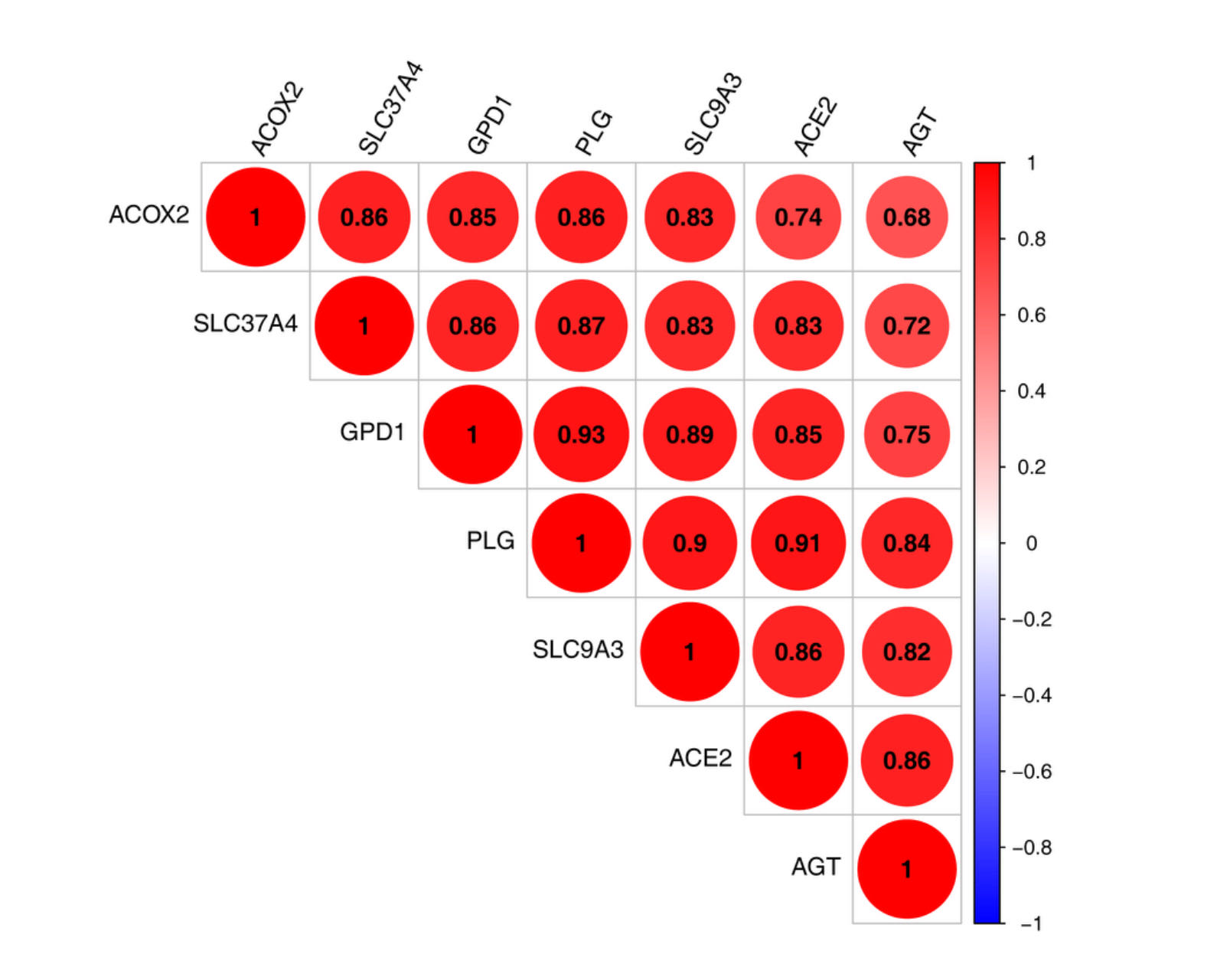

Supplement: Supporting Information 5 — Figure S3: the correlation of 7 biomarkers. [file 9066326.f5.jpg]

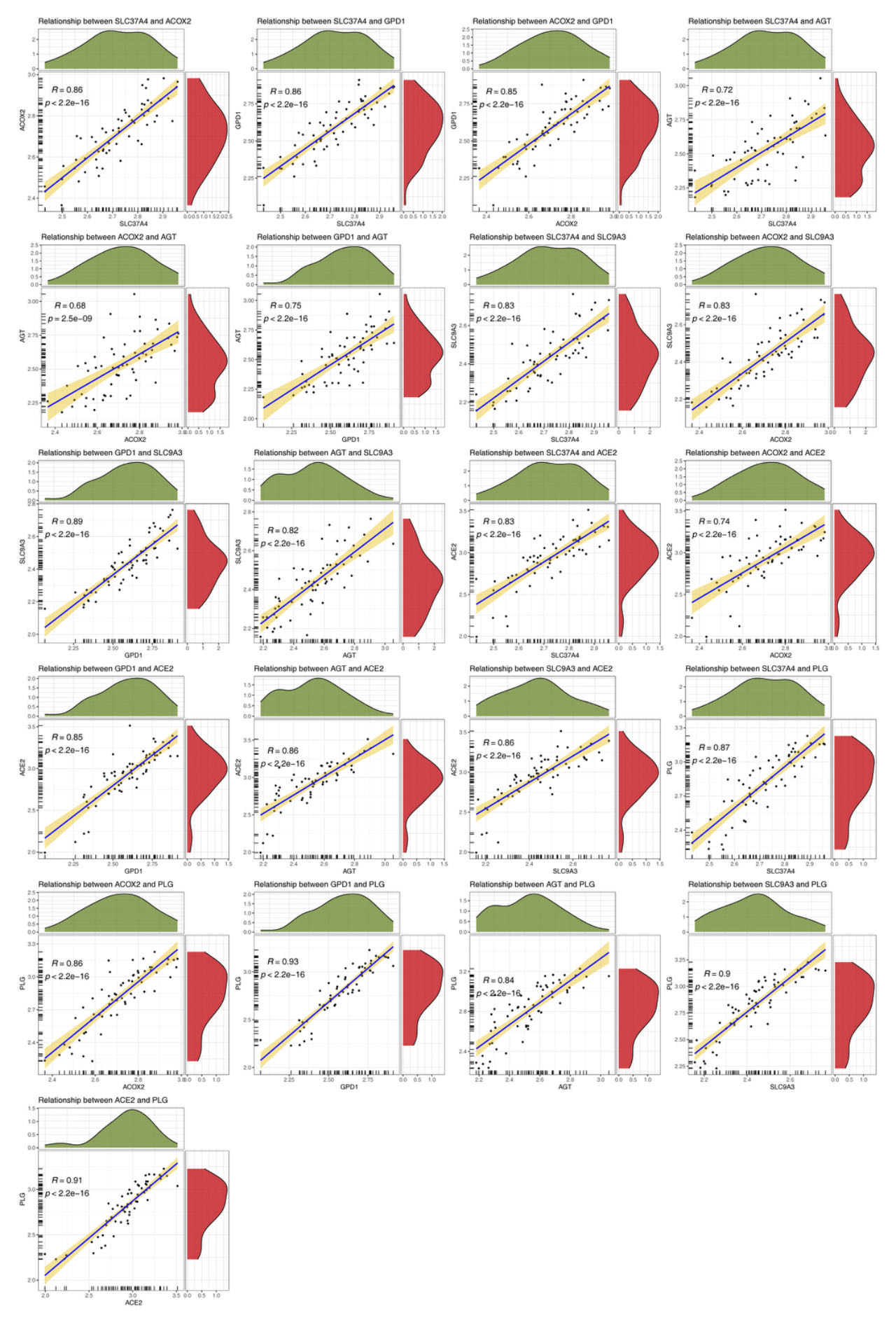

Supplement: Supporting Information 6 — Figure S4: scatter plot of correlation analysis for biomarkers. [file 9066326.f6.jpg]
